# Response of Aboveground Net Primary Production, Species and Phylogenetic Diversity to Warming and Increased Precipitation in an Alpine Meadow

**DOI:** 10.3390/plants12173017

**Published:** 2023-08-22

**Authors:** Jianyu Xiao, Chengqun Yu, Gang Fu

**Affiliations:** 1 Lhasa Plateau Ecosystem Research Station, Key Laboratory of Ecosystem Network Observation and Modeling, Institute of Geographic Sciences and Natural Resources Research, Chinese Academy of Sciences, Beijing 100101, China; xiaojianyu0096@igsnrr.ac.cn (J.X.); yucq@igsnrr.ac.cn (C.Y.); 2 University of Chinese Academy of Sciences, Beijing 100049, China

**Keywords:** biodiversity, α–diversity, β–diversity, climate warming, phylogenetic diversity, species composition

## Abstract

The uncertain responses of aboveground net primary productivity (ANPP) and plant diversity to climate warming and increased precipitation will limit our ability to predict changes in vegetation productivity and plant diversity under future climate change and further constrain our ability to protect biodiversity and ecosystems. A long-term experiment was conducted to explore the responses of ANPP, plant species, phylogenetic α–diversity, and community composition to warming and increased precipitation in an alpine meadow of the Northern Tibet from 2014 to 2019. Coverage, height, and species name were obtained by conventional community investigation methods, and ANPP was obtained using observed height and coverage. Open–top chambers with two different heights were used to simulate low- and high-level climate warming. The low- and high-level increased precipitation treatments were achieved by using two kinds of surface area funnel devices. The high-level warming reduced sedge ANPP (ANPP_sedge_) by 62.81%, species richness (SR) by 21.05%, Shannon by 13.06%, and phylogenetic diversity (PD) by 14.48%, but increased forb ANPP (ANPP_forb_) by 56.65% and mean nearest taxon distance (MNTD) by 33.88%. Species richness, Shannon, and PD of the high-level warming were 19.64%, 9.67%, and 14.66% lower than those of the low-level warming, respectively. The high-level warming-induced dissimilarity magnitudes of species and phylogenetic composition were greater than those caused by low-level warming. The low- rather than high-level increased precipitation altered species and phylogenetic composition. There were significant inter-annual variations of ANPP, plant species, phylogenetic α–diversity and community composition. Therefore, climate warming and increased precipitation had non-linear effects on ANPP and plant diversity, which were due to non-linear changes in temperature, water availability, and/or soil nutrition caused by warming and increased precipitation. The inter-annual variations of ANPP and plant diversity were stronger than the effects of warming and especially increased precipitation on ANPP and plant diversity. In terms of plant diversity conservation and related policy formulation, we should pay more attention to regions with greater warming, at least for the northern Tibet grasslands. Besides paying attention to the responses of ANPP and plant diversity to climate change, the large inter-annual changes of ANPP and plant diversity should be given great attention because the large inter-annual variation indicates the low temporal stability of ANPP and plant diversity and thus produces great uncertainty for the development of animal husbandry.

## 1. Introduction

Aboveground net primary productivity (ANPP) of plants is an important material basis for human survival, and biodiversity is an important guarantee to maintain a high and stable yield of ANPP [1,2,3]. Temperature and precipitation jointly regulate ANPP and plant diversity [4,5]. In the context of global warming and increased precipitation, a growing number of studies have explored the effects of climate warming [6,7], increased precipitation [8,9], or climate warming plus increased precipitation [10] on ANPP and plant diversity. However, two non-completely mutually nonexclusive debates remain. First, does experimental warming or increased precipitation have consistent effects on ANPP and plant diversity? Experimental warming and/or increased precipitation have negligible [10,11], positive [12,13], or negative effects on ANPP and/or plant diversity [14,15]. These diverse effects of warming and increased precipitation on ANPP and plant diversity are related to their distinct vegetation types, soil nutrition conditions, climatic conditions, and the magnitudes of warming or increased precipitation [5,13,16,17,18,19,20,21]. Second, do ANPP and plant diversity respond nonlinearly to experimental warming and/or increased precipitation? ANPP and plant diversity show linear relationships with air temperature and precipitation [12,22,23], indicating ANPP and plant diversity may respond linearly to experiment warming and increased precipitation. In contrast, the magnitude of warming and increased precipitation can not be significantly correlated with the response of ANPP and/or plant diversity to experimental warming and increased precipitation, respectively [24,25], implying that experimental warming and increased precipitation may have nonlinear effects on ANPP and plant diversity. Therefore, it is necessary to further explore the effects of climate warming and increased precipitation on ANPP and plant diversity.

As a sensitive region to climate change, the responses of ANPP and plant diversity to climate change on the Tibetan Plateau are important indicators of the global responses of ANPP and plant diversity to climate change [26,27,28]. Under the background of the Qinghai–Tibet Plateau as a whole tending to be warmer and wetter [1,29,30], a great deal of studies have investigated the effects of climate warming and increased precipitation on ANPP and plant diversity [4,5], which can provide important basic data and scientific theories for the conservation of global plant diversity, the high-quality development of animal husbandry, etc. However, besides the above debates, two other problems remain unresolved. First, several studies have shown a single-level experimental warming [15,31,32] or a multi-level experimental warming effect on ANPP and plant diversity [24,25]. No studies have reported whether there is an optimum warming magnitude for ANPP and plant diversity. Second, compared with α–diversity of plant species, there are few studies on the responses of plant phylogenetic α–diversity to climate change, and plant species and phylogenetic α–diversity have significant differences in reflecting plant α–diversity [1,33,34]. At the same time, compared with plant α–diversity, few studies have examined the responses of plant community composition to climate change, and changes in plant community composition can reflect plant β–diversity, which is essentially different from plant α–diversity [15,19]. Thus, it is not yet clear how ANPP and plant diversity will respond to future climatic change in alpine grasslands on the Qinghai–Tibetan Plateau.

Here, we reported a multi-level warming and increased precipitation experiment in an alpine meadow. The main objectives of this study were to examine (1) whether there was an optimum warming magnitude for the responses of ANPP and plant diversity to warming and whether the low- and high-level warming had different influences on ANPP and plant diversity; (2) whether the responses of ANPP and plant diversity to increased precipitation were related to the magnitude of increased precipitation; and (3) whether the inter-annual variations of ANPP and plant diversity were stronger than the effects of warming and increased precipitation on ANPP and plant diversity in the alpine meadow of Northern Tibet.

## 2. Results

### 2.1. Effects of Experimental Warming and Increased Precipitation on ANPP_community_, ANPP_sedge_, ANPP_graminoid_, ANPP_forb_, Species and Phylogenetic Diversity, and Enviromental Variables

There were significant main effects of experimental warming on ANPP_sedge_, ANPP_forb_, species richness, Shannon, PD, MNTD, species composition, and phylogenetic composition; significant or marginally significant main effects of increased precipitation on ANPP_community_, species composition, and phylogenetic composition; and significant interactive effects of experimental warming and increased precipitation on species composition and phylogenetic composition, respectively (Table 1 and Table 2). There were significant inter-annual variations of ANPP_community_, ANPP_sedge_, ANPP_graminoid_, ANPP_forb_, species richness, Shannon, Simpson, Pielou, PD, MNTD, species composition, and phylogenetic composition (Table 1 and Table 2).

The differences in species richness, Shannon, Simpson, Pielou, PD, MNTD, ANPP_community_, ANPP_sedge_, ANPP_graminoid_, and ANPP_forb_ among the nine treatments varied with years (Figures S1–S3). The low- and high-level experimental warming-induced and the low- and high-level increased precipitation-induced dissimilarity of species composition and phylogenetic composition varied with years (Figures S4 and S5).

The high-level experimental warming reduced ANPP_sedge_ by 62.81% (−2.64 g m^−2^), species richness by 21.05% (−1.56), Shannon by 13.06% (−0.22), PD by 14.48% (−115.29), but increased ANPP_forb_ by 56.65% (5.41 g m^−2^) and MNTD by 33.88% (40.93) across the six growing seasons regardless of increased precipitation. Species richness, Shannon, and PD of the high-level experimental warming was 19.64% (−1.43), 9.67% (−0.15), and 14.66% (−117.02) lower than those of the low-level experimental warming across the six growing seasons regardless of increased precipitation, respectively. The low-level (species composition: *F* = 2.90, *p* = 0.013; phylogenetic composition: *F* = 4.16, *p* = 0.041) and high-level (species composition: *F* = 9.00, *p* = 0.001; phylogenetic composition: *F* = 16.16, *p* = 0.001) experimental warming altered species composition and phylogenetic composition across the six growing seasons regardless of increased precipitation. The high-level experimental warming-induced dissimilarity magnitude of species composition and phylogenetic composition was greater than that caused by low-level experimental warming across the six growing seasons regardless of increased precipitation, respectively (species composition: *F* = 11.10, *p* = 0.002; phylogenetic composition: *F* = 12.13, *p* = 0.001).

The low-level (species composition: *F* = 3.61, *p* = 0.006; phylogenetic composition: *F* = 14.49, *p* = 0.001) rather than the high-level (species composition: *F* = 0.80, *p* = 0.573; phylogenetic composition: *F* = 0.97, *p* = 0.433) increased precipitation altered species composition and phylogenetic composition across the six growing seasons, regardless of experimental warming. However, there were few differences between the low- and high-level increased precipitation-induced dissimilarity magnitudes of species composition (*F* = 1.50, *p* = 0.226) and phylogenetic composition (*F* = 3.15, *p* = 0.082) across the six growing seasons, regardless of experimental warming.

Significant or marginally significant main effects of experimental warming on SM, VPD, GSP/AccT, and *T*_s_ were observed (Table S1, Figure S6). Significant main effects of increased precipitation on *T*_s_, SM, *T*_a_, VPD, AccT, GSP/AccT, NH_4_^+^–N, and AP were detected (Table S1, Figure S6). There were significant or marginally significant interactive effects of experimental warming and increased precipitation on *T*_s_, SM, *T*_a_, VPD, AccT, GSP/AccT, and AP (Table S1, Figure S6). Inter-annual variations of *T*_s_, SM, *T*_a_, VPD, AccT, GSP/AccT, NH_4_^+^–N, NO_3_^−^–N, AP, and pH were observed (Table S1, Figure S6).

**Figure 1:**
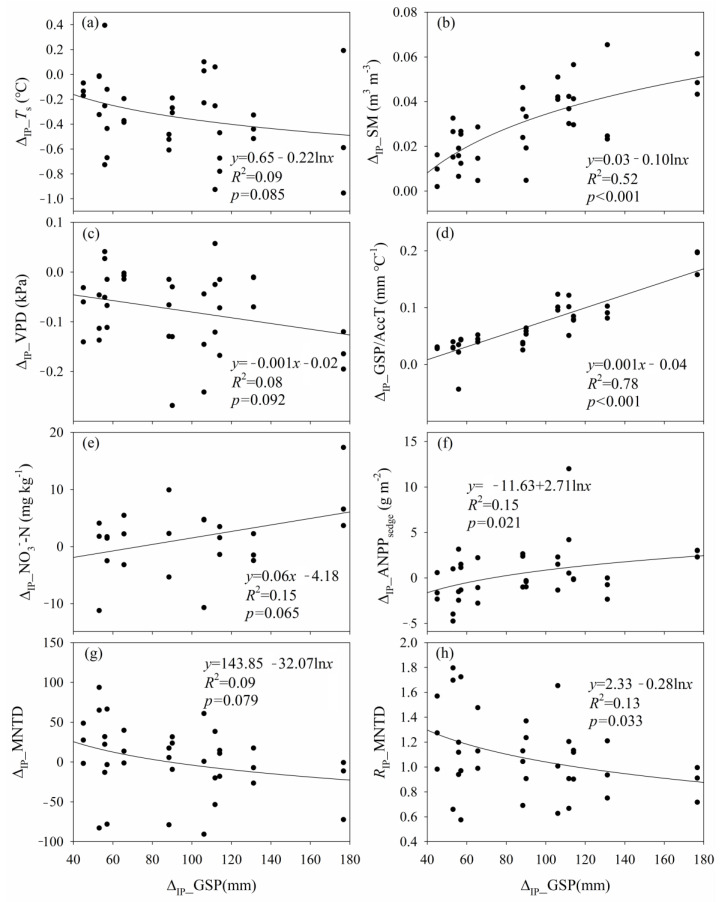
Relationships (**a**) between the change magnitude of soil temperature caused by increased precipitation (Δ_IP__*T*_s_) and increased magnitude of growing season precipitation (Δ_IP__GSP); (**b**) between the increased magnitude of soil moisture caused by increased precipitation (Δ_IP__SM) and Δ_IP__GSP; (**c**) between the change magnitude of vapor pressure deficit caused by increased precipitation (Δ_IP__VPD) and Δ_IP__GSP; (**d**) between the change magnitude of ratio of growing season precipitation to accumulated ≥5 °C daily air temperature caused by increased precipitation (Δ_IP__GSP/AccT) and Δ_IP__GSP; (**e**) between the change magnitude of nitrate nitrogen caused by increased precipitation (Δ_IP__NO_3_^−^–N) and Δ_IP__GSP; (**f**) between the change magnitude of sedge aboveground net primary production caused by increased precipitation (Δ_IP__ANPP_sedge_) and Δ_IP__GSP; (**g**) between the change magnitude of mean nearest taxon distance caused by increased precipitation (Δ_IP__MNTD) and Δ_IP__GSP; and (**h**) between the response ratio of mean nearest taxon distance to increased precipitation (R_IP__MNTD) and Δ_IP__GSP.

The change magnitude of SM caused by experimental warming (Δ_W__SM) was negatively correlated with that of *T*_s_ (Δ_W__*T*_s_) and *T*_a_ (Δ_W__*T*_a_), while that of VPD (Δ_W__VPD) was positively correlated with Δ_W__*T*_s_ and Δ_W__*T*_a_ (Figure S7). The magnitude of the change in GSP/AccT caused by experimental warming (Δ_W__GSP/AccT) decreased with increasing Δ_W__*T*_a_ (Figure S7). Regardless of increased precipitation, the low- and high-level experimental warming increased *T*_s_ by 1.13 °C and 2.59 °C, *T*_a_ by 2.39 °C and 3.86 °C, AccT by 296.26 °C and 476.16 °C, and VPD by 0.20 kPa and 0.35 kPa, but decreased SM by 0.02 m^3^ m^−3^ and 0.05 m^3^ m^−3^, and GSP/AccT by 0.06 °C mm^−1^ and 0.09 °C mm^−1^ across the six growing seasons, respectively. The *T*_s_, *T*_a_, AccT, and VPD of the high-level experimental warming were greater than those of the low-level experimental warming, whereas the SM and GSP/AccT of the high-level experimental warming were lower than those of the low-level experimental warming, respectively. Increased precipitation-induced change magnitudes of *T*_s_ (Δ_IP__*T*_s_) and VPD (Δ_IP__VPD) decreased with increasing that of growing season precipitation (Δ_IP__GSP), but that of SM (Δ_IP__SM) and GSP/AccT (Δ_IP__GSP/AccT) increased with increasing Δ_IP__GSP (Figure 1). Regardless of experimental warming, the low- and high-level increased precipitation increased SM by 0.02 m^3^ m^−3^ and 0.04 m^3^ m^−3^, and GSP/AccT by 0.03 °C mm^−1^ and 0.10 °C mm^−1^, but decreased *T*_s_ by 0.28 °C and 0.36 °C, and VPD by 0.05 kPa and 0.10 kPa across the six growing seasons, respectively. The SM and GSP/AccT of the high-level increased precipitation were greater than those of the low-level increased precipitation, respectively.

The comparison of soil NH_4_^+^–N, NO_3_^−^–N, AP, and pH among the nine treatments were illustrated in Figure S8. The change magnitude of NO_3_^−^–N caused by increased precipitation (Δ_IP__NO_3_^−^–N) increased with increasing Δ_IP__GSP (Figure 1). Regardless of increased precipitation, the low- and high-level experimental warming increased soil NH_4_^+^–N by 30.93% (3.02 mg kg^−1^) and 32.74% (3.20 mg kg^−1^), and soil AP by 28.60% (2.49 mg kg^−1^) and 28.81% (2.51 mg kg^−1^) across the four growing seasons, respectively. The high-level experimental warming increased soil NO_3_^−^–N by 74.47% (9.51 mg kg^−1^), and the soil NO_3_^−^–N of the high-level experimental warming was 43.92% greater than that of the low-level experimental warming across the four growing seasons.

### 2.2. Relationships between Experimental Warming–Induced Change Magnitude and Response Ratio of Biotic Variables, βBray_W_ and βMNTD_W_, and Experimental Warming–Induced Change Magnitude of Abiotic Variables and Warming Duration

**Figure 2:**
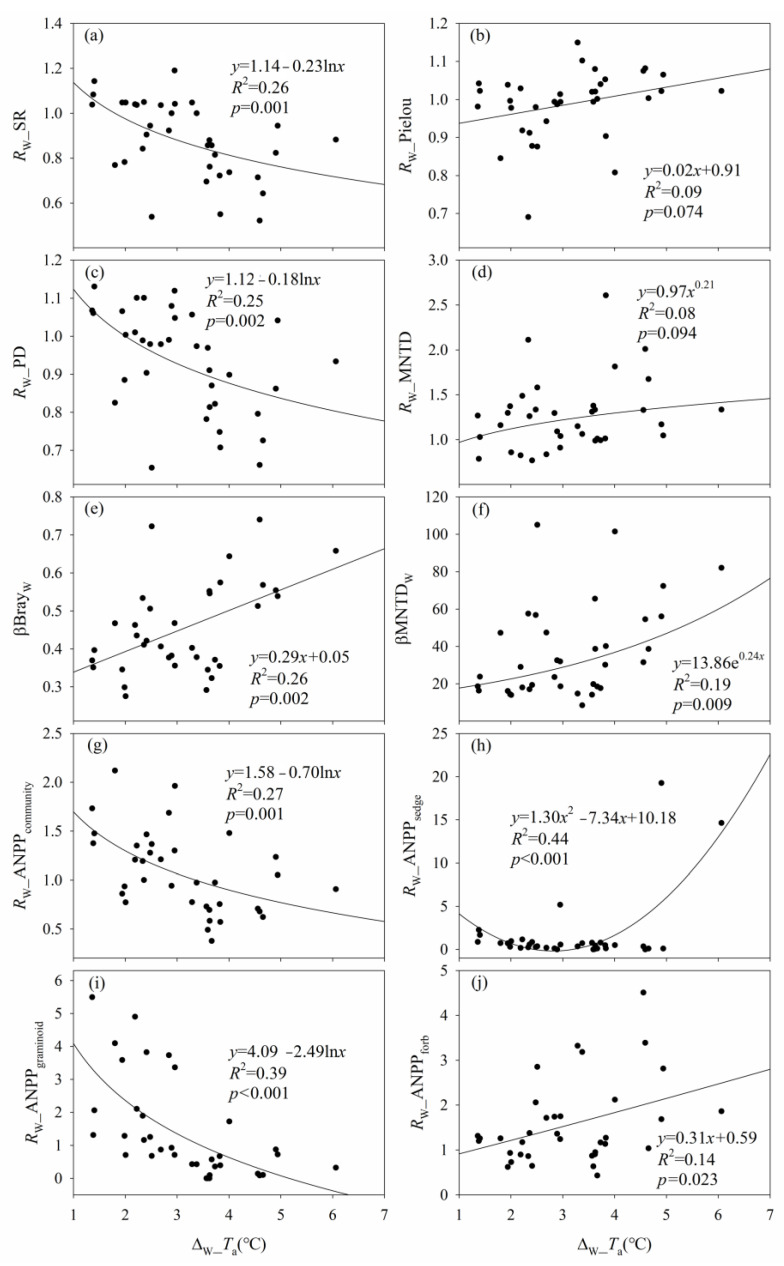
Relationships (**a**) between the response ratio of species richness to experimental warming (*R*_W__SR) and increased magnitude of air temperature caused by experimental warming (Δ_W__*T*_a_); (**b**) between the response ratio of Pielou to experimental warming (*R*_W__Pielou) and Δ_W__*T*_a_; (**c**) between the response ratio of Faith’s phylogenetic diversity to experimental warming (*R*_W__PD) and Δ_W__*T*_a_; (**d**) between the response ratio of mean nearest taxon distance to experimental warming (*R*_W__MNTD) and Δ_W__*T*_a_; (**e**) between species β–diversity (βBray_w_) and Δ_W__*T*_a_; (**f**) between phylogenetic β–diversity (βMNTD_W_) and Δ_W__*T*_a_; (**g**) between the response ratio of community aboveground net primary production to experimental warming (*R*_W__ANPP_community_) and Δ_W__*T*_a_; (**h**) between the response ratio of sedge aboveground net primary production to experimental warming (*R*_W__ANPP_sedge_) and Δ_W__*T*_a_; (**i**) between the response ratio of graminoid aboveground net primary production to experimental warming (*R*_W__ANPP_graminoid_) and Δ_W__*T*_a_; and (**j**) between the response ratio of forb aboveground net primary production to experimental warming (*R*_W__ANPP_forb_) and Δ_W__*T*_a_.

The effects of warming on species richness, PD, ANPP_community_, and ANPP_graminoid_ decreased with warming magnitude (Figure 2 and Figure S9–S11). The effects of warming on Pielou, MNTD, and ANPP_forb_ increased with Δ_W__*T*_a_ (Figure 2 and Figure S9). The effects of warming on Pielou and ANPP_forb_ showed quadratic relationships with Δ_W__*T*_s_ (Figures S10 and S11). The response ratio of ANPP_sedge_ to experimental warming (*R*_W__ANPP_sedge_) and the change magnitude of ANPP_sedge_ caused by experimental warming (Δ_W__ANPP_sedge_) both showed quadratic relationships with Δ_W__*T*_a_, and *R*_W__ANPP_sedge_ and Δ_W__ANPP_sedge_ reached their minimum values when Δ_W__*T*_a_ was about 2.82 °C and 3.57 °C, respectively (Figure 2 and Figure S9). The higher was Δ_W__*T*_a_, the greater were the warming-induced differences in species composition (βBray_W_) and phylogenetic composition (βMNTD_W_) (Figure 2). The βBray_W_ showed a quadratic relationship with Δ_W__*T*_s_, and the βBray_W_ reached its maximum value when Δ_W__*T*_s_ was about 2.08 °C (Figure S10).

The greater the experimental warming-induced soil and/or air drying, the greater the experimental warming-induced reductions in species and phylogenetic α–diversity, ANPP_community_, ANPP_sedge_, and ANPP_graminoid_ (Figures S12 and S13). The effect of warming on ANPP_forb_ showed opposite relationships with experimental warming-induced soil and air drying (Figures S12 and S13). The reduction in GSP/AccT caused by experimental warming can cause species loss, ANPP_graminoid_ reduction, and ANPP_forb_ increase (Figures S12 and S13). The greater the experimental warming-induced reduction in GSP/AccT, the greater the warming-induced differences in species composition (βBray_W_) and phylogenetic composition (βMNTD_W_) (Figure S12).

The effects of experimental warming on species richness Shannon, Simpson, and PD showed quadratic relationships with the magnitude of the change in soil pH caused by experimental warming (Δ_W__pH), and βMNTD_W_ decreased with Δ_W__pH (Figures S14 and S15). The effects of experimental warming on species α–diversity, ANPP_community_, PD, ANPP_sedge_, and ANPP_graminoid_ decreased with increasing Δ_W__NO_3_^−^–N (the change magnitude of NO_3_^−^–N caused by experimental warming), but the effect of experimental warming on MNTD increased with increasing Δ_W__NO_3_^−^–N (Figures S14 and S15). Both βBray_W_ and βMNTD_W_ increased with increasing Δ_W__NO_3_^−^–N (Figure S14). The effect of experimental warming on ANPP_forb_ showed quadratic relationships with the change magnitude of NH_4_^+^–N caused by experimental warming (Δ_W__NH_4_^+^–N) (Figures S14 and S15).

**Figure 3:**
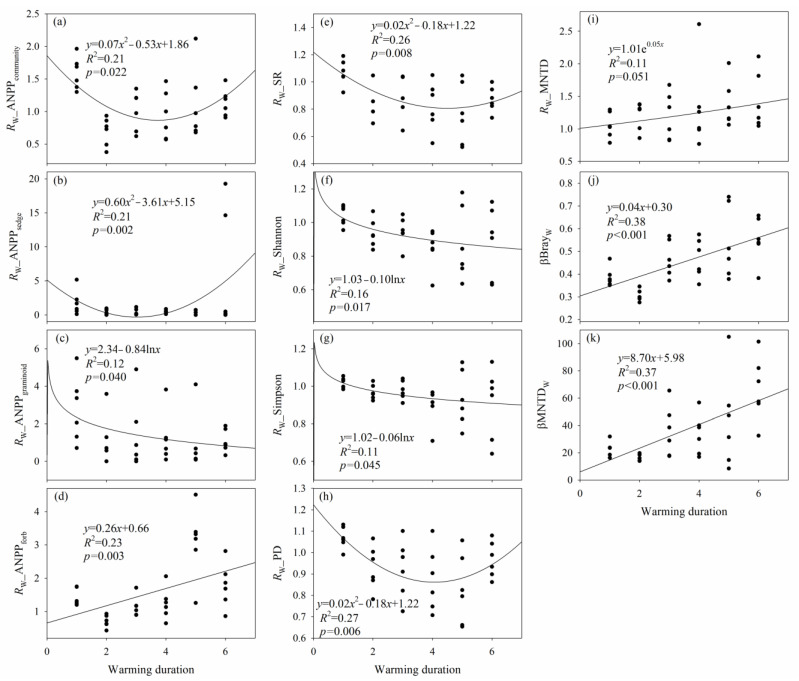
Relationships (**a**) between the response ratio of community aboveground net primary production to experimental warming (*R*_W__ANPP_community_) and warming duration; (**b**) between the response ratio of sedge aboveground net primary production to experimental warming (*R*_W__ANPP_sedge_) and warming duration; (**c**) between the response ratio of graminoid aboveground net primary production to experimental warming (*R*_W__ANPP_graminoid_) and warming duration; (**d**) between the response ratio of forb aboveground net primary production to experimental warming (*R*_W__ANPP_forb_) and warming duration; (**e**) between the response ratio of species richness to experimental warming (*R*_W__SR) and warming duration; (**f**) between the response ratio of Shannon to experimental warming (*R*_W__Shannon) and warming duration; (**g**) between the response ratio of Simpson to experimental warming (*R*_W__Simpson) and warming duration; (**h**) between the response ratio of Faith’s phylogenetic diversity to experimental warming (*R*_W__PD) and warming duration; (**i**) between the response ratio of mean nearest taxon distance to experimental warming (*R*_W__MNTD) and warming duration; (**j**) between species β–diversity of warming versus non–warming conditions (βBray_W_) and warming duration; and (**k**) between phylogenetic β–diversity of warming versus non-warming conditions (βMNTD_W_) and warming duration.

The effects of warming on Shannon, Simpson, and ANPP_graminoid_ decreased with warming duration, and the effects of warming on MNTD and ANPP_forb_ increased with warming duration (Figure 3 and Figure S16). Moreover, the effects of warming on species richness, PD, ANPP_community_, and ANPP_sedge_ showed quadratic relationships with warming duration, and there was a time point when the lowest effect of warming on species richness, PD, ANPP_community_, and ANPP_sedge_ occurred, respectively (Figure 3 and Figure S16). The longer the warming duration, the greater the warming-induced differences in species composition (βBray_W_) and phylogenetic composition (βMNTD_W_) (Figure 3).

**Figure 4:**
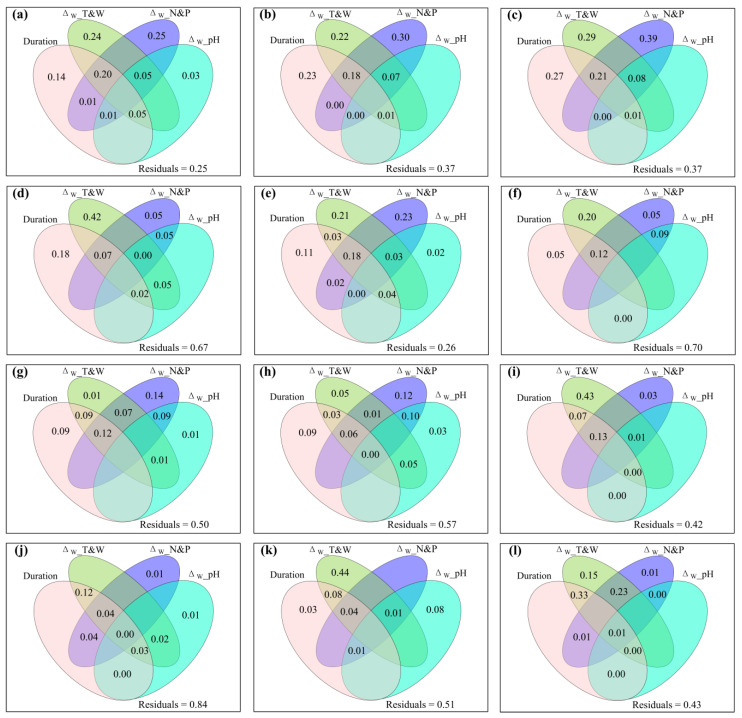
Venn plots of variation partitioning analysis, showing the shared and exclusive effects of warming duration, Δ_W__T&W (i.e., the change magnitude of air and/or soil temperature, soil moisture, vapor pressure deficit and/or the ratio of growing season precipitation to accumulated ≥5 °C daily air temperature caused by experimental warming), Δ_W__N&P (i.e., the change magnitude of ammonium nitrogen, nitrate nitrogen, and/or available phosphorus caused by experimental warming) and Δ_W__pH (i.e., the change magnitude of soil pH caused by experimental warming) on (**a**) the response ratio of species richness to experimental warming (*R*_W__SR); (**b**) the response ratio of Shannon to experimental warming (*R*_W__Shannon); (**c**) the response ratio of Simpson to experimental warming (*R*_W__Simpson); (**d**) the response ratio of Pielou to experimental warming (*R*_W__Pielou); (**e**) the response ratio of Faith’s phylogenetic diversity to experimental warming (*R*_W__PD); (**f**) the response ratio of mean nearest taxon distance to experimental warming (*R*_W__MNTD); (**g**) species β–diversity (βBrayw) between the warming and non–warming conditions; (**h**) phylogenetic β–diversity (βMNTDw) between the warming and non–warming conditions; (**i**) the response ratio of community aboveground net primary production to experimental warming (*R*_W__ANPP_community_); (**j**) the response ratio of sedge aboveground net primary production to experimental warming (*R*_W__ANPP_sedge_); (**k**) the response ratio of graminoid aboveground net primary production to experimental warming (*R*_W__ANPP_graminoid_); and (**l**) the response ratio of forb aboveground net primary production to experimental warming (*R*_W__ANPP_forb_).

The varpart results showed that warming duration, experimental warming-induced changes in environment temperature and moisture, soil nitrogen and phosphorus, and/or soil pH together controlled the variation of species, phylogenetic diversity, and aboveground plant production under controlled warming conditions (Figures S17–S21 and Figure 4).

### 2.3. Relationships between Experimental Warming–Induced Change Magnitude and Response Ratio of ANPP_community_ and Experimental Warming–Induced Change Magnitude and Response Ratio of α–Diversity, βBray_W_ and βMNTD_W_

*R*_W__ANPP_community_ increased with increasing *R*_W__SR and *R*_W__PD, but decreased with increasing *R*_W__Pielou (Figure S22). Δ_W__ANPP_community_ decreased with increasing Δ_W__Pielou and Δ_W__Simpson but increased with increasing βMNTD_W_ (Figure S22). The effect of experimental warming on the ANPP_community_ was simultaneously regulated by species and phylogenetic α– and β–diversity (Figure S23).

### 2.4. Relationships between Increased Precipitation–Induced Change Magnitude and Response Ratio of Biotic Variables, βBray_IP_ and ΒMNTD_IP_, and Abiotic Variables and Increased Precipitation Duration

Increased precipitation-induced change magnitude of ANPP_sedge_ (Δ_IP__ANPP_sedge_) increased with increasing Δ_IP__GSP, and there was a turning point from a negative effect of increased precipitation to a positive effect of increased precipitation on ANPP_sedge_ (Figure 1). In contrast, increased precipitation-induced change magnitude of MNTD (Δ_IP__MNTD) and increased precipitation-induced response ratio of MNTD (*R*_IP__MNTD) decreased with increasing Δ_IP__GSP, and there was a turning point from a positive effect of increased precipitation to a negative effect of increased precipitation on MNTD (Figure 1).

Increased precipitation-induced change in *T*_s_, SM, *T*_a_, VPD, NH_4_^+^–N, NO_3_–N, and/or AP may have some relationships with the effects of increased precipitation on species, phylogenetic diversity, and aboveground plant production (Figures S24–S27). For example, the effect of increased precipitation on species richness increased with Δ_IP__AP (the change in magnitude of AP caused by increased precipitation) (Figure S26).

**Figure 5:**
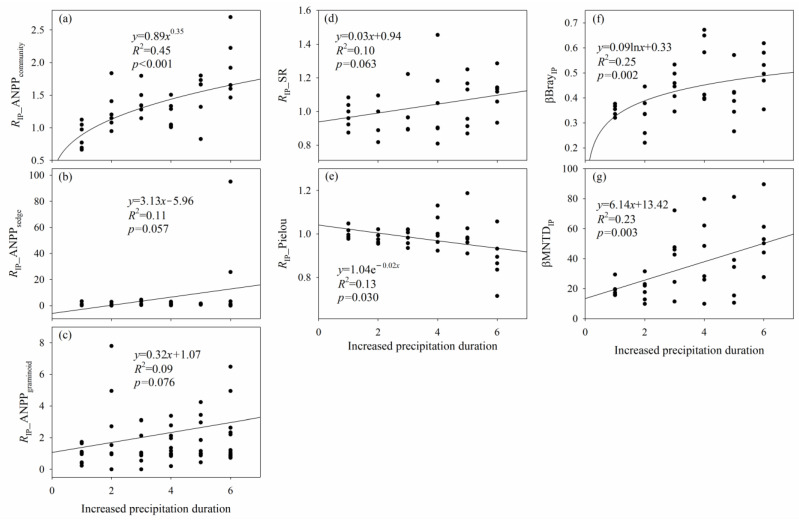
Relationships (**a**) between the response ratio of community aboveground net primary production to increased precipitation (*R*_IP__ANPP_community_) and increased precipitation duration; (**b**) between the response ratio of sedge aboveground net primary production to increased precipitation (*R*_IP__ANPP_sedge_) and increased precipitation duration; (**c**) between the response ratio of graminoid aboveground net primary production to increased precipitation (*R*_IP__ANPP_graminoid_) and increased precipitation duration; (**d**) between the response ratio of species richness to increased precipitation (*R*_IP__SR) and increased precipitation duration; (**e**) between the response ratio of Pielou to increased precipitation (*R*_IP__Pielou) and increased precipitation duration; (**f**) between species β–diversity of increased precipitation versus non–increased precipitation conditions (βBray_IP_) and increased precipitation duration; and (**g**) between phylogenetic β–diversity of increased precipitation versus non–increased precipitation conditions (βMNTD_IP_) and increased precipitation duration.

The effects of increased precipitation on SR, ANPP_community_, ANPP_sedge_, and ANPP_graminoid_ increased with increased precipitation duration, and the effect of increased precipitation on Pielou decreased with increased precipitation duration (Figure 5 and Figure S28). There was a turning point from a negative effect of increased precipitation to a positive effect of increased precipitation on SR, ANPP_community_, ANPP_sedge_, and ANPP_graminoid_, respectively (Figure 5 and Figure S28). There was a turning point from a positive effect of increased precipitation to a negative effect of increased precipitation on Pielou (Figure 5 and Figure S28). The longer the increased-precipitation duration, the greater the increased precipitation-induced difference in species composition (βBray_IP_) and phylogenetic composition (βMNTD_IP_) (Figure 5).

**Figure 6:**
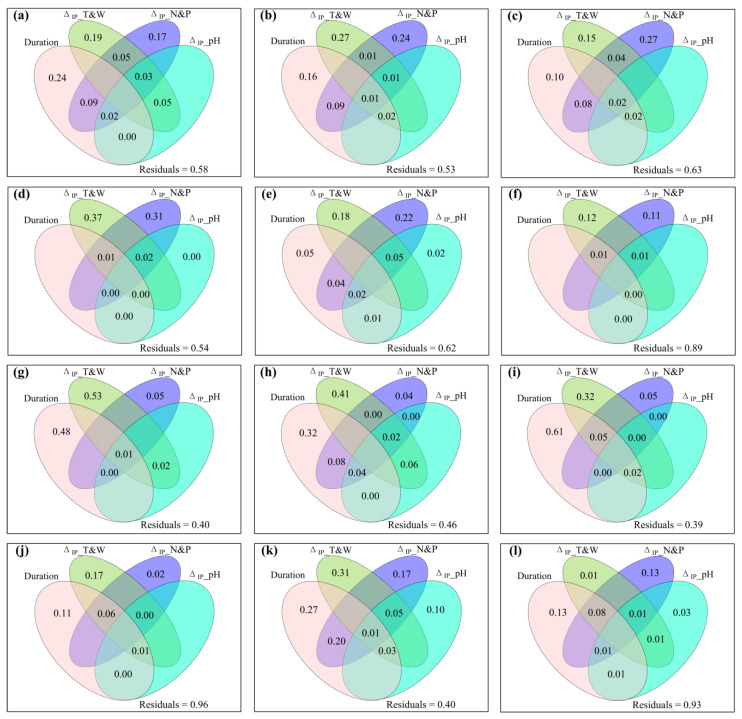
Venn plots of variation partitioning analysis, showing the shared and exclusive effects of increased precipitation duration, Δ_IP__T&W (i.e., the change magnitude of air and/or soil temperature, soil moisture, vapor pressure deficit and/or the ratio of growing season precipitation to accumulated ≥5 °C daily air temperature caused by increased precipitation), Δ_IP__N&P (i.e., the change magnitude of ammonium nitrogen, nitrate nitrogen, and/or available phosphorus caused by increased precipitation) and Δ_IP__pH (i.e., the change magnitude of soil pH caused by increased precipitation) on (**a**) the response ratio of species richness to increased precipitation (*R*_IP__SR); (**b**) the response ratio of Shannon to increased precipitation (*R*_IP__Shannon); (**c**) the response ratio of Simpson to increased precipitation (*R*_IP__Simpson); (**d**) the response ratio of Pielou to increased precipitation (*R*_IP__Pielou); (**e**) the response ratio of Faith’s phylogenetic diversity to increased precipitation (*R*_IP__PD); (**f**) the response ratio of mean nearest taxon distance to increased precipitation (*R*_IP__MNTD); (**g**) species β–diversity (βBray_w_) between the increased precipitation and non–increased precipitation conditions; (**h**) phylogenetic β–diversity (βMNTD_w_) between the increased precipitation and non–increased precipitation conditions; (**i**) the response ratio of community aboveground net primary production to increased precipitation (*R*_IP__ANPP_community_); (**j**) the response ratio of sedge aboveground net primary production to increased precipitation (*R*_IP__ANPP_sedge_); (**k**) the response ratio of graminoid aboveground net primary production to increased precipitation (*R*_IP__ANPP_graminoid_); and (**l**) the response ratio of forb aboveground net primary production to increased precipitation (*R*_IP__ANPP_forb_).

The shared and excluded effects of increased precipitation duration, Δ_IP__T (Δ_IP__*T*_s_ and/or Δ_IP__*T*_a_), Δ_IP__W (Δ_IP__GSP, Δ_IP__SM, and/or Δ_IP__VPD), and Δ_IP__GSP/AccT on the response ratio of biotic variables to increased precipitation and the change magnitude of biotic variables caused by increased precipitation were illustrated in Figures S29 and S30, respectively. The shared and excluded effects of increased precipitation duration, Δ_IP__N (Δ_IP__NH_4_^+^–N and/or Δ_IP__NO_3_^−^–N), Δ_IP__AP and Δ_IP__pH on the response ratio of biotic variables to increased precipitation and the change magnitude of biotic variables caused by increased precipitation were illustrated in Figures S31 and S32, respectively. The shared and exclusive effects of increased precipitation duration, Δ_IP__T&W (Δ_IP__*T*_s_, Δ_IP__*T*_a_, Δ_IP__GSP, Δ_IP__SM, Δ_IP__VPD and/or Δ_IP__GSP/AccT), Δ_IP__N&P (Δ_IP__NH_4_^+^–N, Δ_IP__NO_3_^−^–N and/or Δ_IP__AP), and Δ_IP__pH on the response ratio of biotic variables to increased precipitation and the change magnitude of biotic variables caused by increased precipitation were illustrated in Figure 6 and Figure S33, respectively. These varpart results showed that increased precipitation duration, increased precipitation-induced changes in environment temperature and moisture, soil nitrogen and phosphorus, and/or soil pH together controlled the variation of species, phylogenetic diversity, and aboveground plant production under controlled increased precipitation conditions.

### 2.5. Relationships between Increased–Precipitation–Induced Change Magnitude and Response Ratio of ANPP_community_ and Increased–Precipitation–Induced Change Magnitude and Response Ratio of α–Diversity, βBray_IP_ and βMNTD_IP_

*R*_IP__ANPP_community_ decreased with increasing *R*_IP__Shannon, *R*_IP__Simpson and *R*_IP__Pielou, and Δ_IP__ANPP_community_ decreased with increasing Δ_IP__Shannon, Δ_IP__Simpson and Δ_IP__Pielou (Figure S34). Both *R*_IP__ANPP_community_ and Δ_IP__ANPP_community_ increased with increasing βBray_IP_ and βMNTD_IP_ (Figure S34). The effect of increased precipitation on ANPP_community_ was simultaneously regulated by species and phylogenetic α– and β–diversity under increased precipitation conditions (Figure S35).

### 2.6. Shared and Excluded Effects of Experimental Duration, Environmental Variables, Species and Phylogenetic Diversity on ANPP_community_

**Figure 7:**
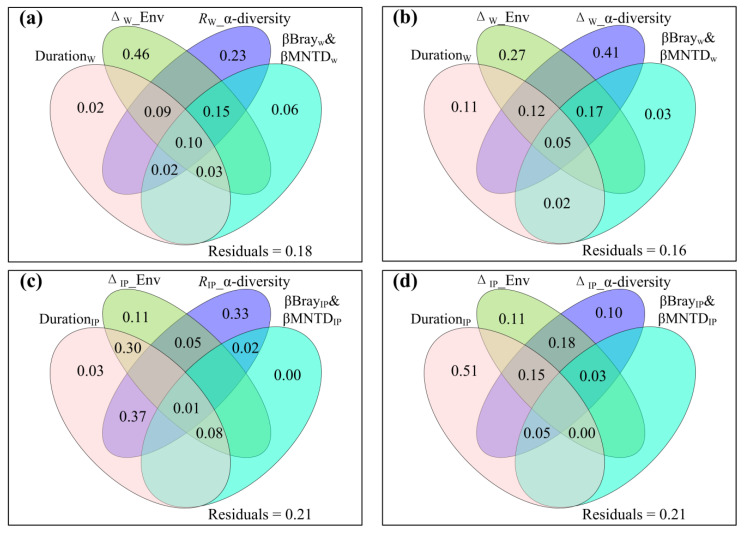
Venn plots of variation partitioning analysis, showing (**a**) the shared and exclusive effects of duration_W_ (warming duration), Δ_W__Env (the change magnitude of soil temperature, air temperature, soil moisture, vapor pressure deficit, ratio of growing season precipitation to accumulated ≥5 °C daily air temperature, ammonium nitrogen, nitrate nitrogen, available phosphorus, and/or pH caused by experimental warming), *R*_W__α–diversity (the response ratio of species richness, Shannon, Simpson, Pielou, Faith’s phylogenetic diversity and/or mean nearest taxon distance to experimental warming) and βBrayw&βMNTDw (species and phylogenetic β–diversity of warming versus non–warming conditions) on the response ratio of community aboveground net primary production to experimental warming (*R*_W__ANPPcommunity); (**b**) the shared and exclusive effects of durationw, Δ_W__Env, Δ_W__α–diversity (the change magnitude of species richness, Shannon, Simpson, Pielou, Faith’s phylogenetic diversity and/or mean nearest taxon distance caused by experimental warming) and βBray_W_&Βmntd_W_ on the change magnitude of community aboveground net primary production caused by experimental warming (Δ_W__ANPP_community_); (**c**) the shared and exclusive effects of duration_IP_ (increased precipitation duration), Δ_IP__Env (the change magnitude of soil temperature, air temperature, soil moisture, vapor pressure deficit, ratio of growing season precipitation to accumulated ≥5 °C daily air temperature, ammonium nitrogen, nitrate nitrogen, available phosphorus, and/or pH caused by increased precipitation), *R*_IP__α–diversity (the response ratio of species richness, Shannon, Simpson, Pielou, Faith’s phylogenetic diversity, and/or mean nearest taxon distance to increased precipitation) and βBray_IP_&Βmntd_IP_ (species and phylogenetic β–diversity of increased precipitation versus non–increased precipitation conditions) on the response ratio of community aboveground net primary production to increased precipitation (*R*_IP__ANPPcommunity); and (**d**) the shared and exclusive effects of duration_IP_, Δ_IP__Env, Δ_IP__α–diversity (the change magnitude of species richness, Shannon, Simpson, Pielou, Faith’s phylogenetic diversity, and/or mean nearest taxon distance caused by increased precipitation) and βBray_IP_&ΒMNTD_IP_ on the change magnitude of community aboveground net primary production caused by increased precipitation (Δ_IP__ANPP_community_).

All the variables in the *varpart* analysis explained about 82%, 84%, 79%, and 79% variations of *R*_W__ANPP_community_, Δ_W__ANPP_community_, *R*_IP__ANPP_community_, and Δ_IP__ANPP_community_, respectively (Figure 7). The excluded effects on *R*_W__ANPP_community_ was in an order of Δ_W__Env, *R*_W__α–diversity, βBray_W_&βMNTD_W_ and Duration_w_ (Figure 7a). The excluded effects on Δ_W__ANPP_community_ was in an order of Δ_W__α–diversity, Δ_W__Env, Duration_w_, and βBray_W_&βMNTD_W_ (Figure 7b). The excluded effects on *R*_IP__ANPP_community_ was in an order of *R*_IP__α–diversity, Δ_IP__Env, Duration_IP_, and βBray_IP_&βMNTD_IP_ (Figure 7c). The excluded effects on Δ_IP__ANPP_community_ were in the order of Duration_IP,_ Δ_IP__Env and Δ_IP__α–diversity, and the βBray_IP_&βMNTD_IP_ had no excluded effect on Δ_IP__ANPP_community_ (Figure 7d).

## 3. Discussion

### 3.1. Warming Effects

The high-level warming caused greater reductions in species richness and PD than did the low-level warming. First, low temperature is a key limited factor for alpine growth, but excessive temperature may lead to the death of temperature-sensitive species and, in turn, species loss [4,14] and the decline in PD. A greater warming can often cause greater increases in *T*_s_ and *T*_a_ [10,35]. Second, water availability is another key limited factor for plant growth, and drying may lead to stomatal closure and photorespiration [17,36]. Warming-induced drying may cause the death of drying-sensitive species and, in turn, the loss of species and related phylogenetic information [37,38]. A greater warming can often cause greater soil and air drying [10,35]. Third, GSP/AccT is often positively correlated with plant growth [4,10,39]. The greater decline in GSP/AccT caused by high-level warming caused a greater reduction in species richness and related phylogenetic information. Fourth, soil NO_3_^−^–N is an important nitrogen resource for alpine plant growth [2,3,20,40,41,42], and there can be species-specific preferences for soil NO_3_^−^–N [43,44]. However, only high-level warming increased soil NO_3_^−^–N.

The high-level warming caused a greater reduction in Shannon than did the low-level warming, which may be due to the high-level warming-induced greater reduction in SM and a greater increase in NO_3_^−^–N (Figures S12–S15). The high- rather than low-level warming increased MNTD, which may be due to the greater increase in *T*_a_ and NO_3_^−^–N, and the greater reduction in GSP/AccT caused by the high-level warming (Figure 2, Figures S9 and S12–S15).

Low- and high-level warming did not alter Simpson and Pielou. First, the effect of warming on Simpson may be mainly related to warming-induced increases in NO_3_^−^–N, whereas the effect of warming on Pielou may be mainly related to warming–induced increases in *T*_s_ and *T*_a_ (Figure 2, Figures S9–S11, S14 and S15). Second, the low- and high-level warming-induced increases in *T*_s_, *T*_a_, and NO_3_^−^–N were close to the turning points of *T*_s_, *T*_a_, and NO_3_^−^–N where the *R*_W__Simpson and *R*_W__Pielou were equal to 1, or Δ_W__Simpson and Δ_W__Pielou were equal to zero (Figure 2, Figures S9–S11, S14 and S15). Third, the decreased magnitude of Simpson increased with warming duration (Figure 3 and Figure S16), which indicated that short-term warming caused a negligible effect on Simpson.

The high-level warming resulted in greater dissimilarities in species and phylogenetic composition than did the low-level warming. First, there was an optimum of Δ_W__*T*_s_ (about 2.08 °C) when the βBray_W_ was the greatest. The high-level warming-induced by the increase in the Δ_W__*T*_s_ (about 2.59 °C) was closer to the optimum of Δ_W__*T*_s_ than the low-level warming–induced in the increase in the Δ_W__*T*_s_ (about 1.13 °C) (Figure S10). Second, compared to the low-level warming-induced lower increases in Δ_W__*T*_a_ and Δ_W__NO_3_^−^–N, the high-level warming-induced greater increases in Δ_W__*T*_a_ and Δ_W__NO_3_^−^–N can tend to cause greater βBray_W_ and βMNTD_W_ (Figure 2 and Figure S14). Third, compared to the low-level warming-induced lower reduction in GSP/AccT, the high-level warming-induced greater reduction in GSP/AccT can tend to cause greater βBray_W_ and βMNTD_W_ (Figure S12).

The high- rather than low-level warming reduced ANPP_sedge_, which was similar to a previous study demonstrating an increase in *T*_a_ can reduce sedge coverage in a Northern Tibet alpine meadow [25], and may be due to the high-level warming-induced greater increase in *T*_a_, soil drying, AP, and NO_3_^−^–N (Figures S9 and S13–S15).

The low- and high-level warming did not alter ANPP_graminoid_, and there was no difference in ANPP_graminoid_ between the low- and high-level warming. Similarly, no significant main effect of warming on graminoid coverage was observed in a four-level warming (control, +1.00, +2.70, and +4.00 °C, respectively) experiment [45]. First, the low-level warming–induced the increases in *T*_s_, *T*_a_ and VPD tended to increase *R*_W__ANPP_graminoid_ and Δ_W__ANPP_graminoid_, but the high-level warming-induced increases in *T*_s_, *T*_a_, and/or VPD tended to reduce *R*_W__ANPP_graminoid_ and Δ_W__ANPP_graminoid_ (Figure 2 and Figure S10). The low-level warming-induced reduction in SM tended to increase Δ_W__ANPP_graminoid_, but the high-level warming-induced reduction in SM tended to decrease Δ_W__ANPP_graminoid_ (Figure S13). The absolute values of the changes of *R*_W__ANPP_graminoid_ caused by the increases in *T*_s_, *T*_a_, and VPD under the low-level warming were greater than those under the high-level warming, whereas the absolute values of the changes of Δ_W__ANPP_graminoid_ caused by the changes in *T*_s_, *T*_a_ and SM under the low-level warming were lower than those under the high-level warming. Second, the low- and high-level warming-induced the reduction in GSP/AccT tended to increase the *R*_W__ANPP_graminoid_ (Figure S12), and with a greater increase caused by the low-level warming-induced change in GSP/AccT. Third, the high- rather than the low-level warming-induced the increase in NO_3_^−^–N tended to increase *R*_W__ANPP_graminoid_ (Figure S14). Fourth, the low- and high-level warming-induced the increase in NH_4_^+^–N tended to increase the Δ_W__ANPP_graminoid_ (Figure S15), and with a greater increase caused by the low-level warming-induced change in GSP/AccT. Fifth, a greater reduction in ANPP_graminoid_ can occur along with warming duration (Figure 3), which indicated that short–term warming (<7 years) caused negligible effect of warming on ANPP_graminoid_.

The high- rather than low-level warming increases ANPP_forb_. First, the high-level warming-induced greater increase in *T*_s_ and *T*_a_ tended to cause a greater increase in *R*_W__ANPP_forb_ and Δ_W__ANPP_forb_ than did the low-level warming (Figure 2 and Figures S9–S11). Second, the high-level warming-induced greater soil drying and reduction in GSP/AccT tended to cause a greater increase in *R*_W__ANPP_forb_ and Δ_W__ANPP_forb_ than did the low-level warming (Figures S12 and S13).

The low- and high-level warming did not alter ANPP_community_, and there was no difference in ANPP_community_ between the low- and high-level warming. Likewise, no main effect of warming on ANPP_community_ was detected in a four-level (control, +1.00, +2.70, and +4.00 °C, respectively) [45] and five-level warming experiment (control, +1.13, +1.66, +2.10, and +2.72 °C, respectively) [25]. There was a negligible difference in ANPP between low- and high-level warming (1.05 and 1.69 °C, respectively) in an alpine meadow [24]. First, the quite contrary effects of warming on ANPP_sedge_ and ANPP_forb_ may weaken the effect of warming on ANPP_community_. Meanwhile, the negligible effect of warming on ANPP_graminoid_ can further weaken the warming effect on ANPP_community_. Second, considering the obvious relationships of ANPP_community_ with Simpson and Pielou (Figure S22), the negligible effect of the low- and high-level warming on ANPP_community_ should be related to that on Simpson and Pielou. Third, warming-induced changes in *T*_s_, *T*_a_, VPD, SM, NO_3_–N, and AP may have exclusive effects on ANPP_community_. For example, drying climate conditions can dampen and even mask the effect of increased *T*_s_ and/or *T*_a_ on ANPP_community_ in grasslands [25,45] by reducing leaf area and inducing stomatal closure [46], and the average GSP (405.5 mm) in 2014–2019 was only equal to that (400.5 mm) in 1963–2019.

### 3.2. Increased Precipitation Effects

Increased precipitation did not change species and phylogenetic α–diversity, ANPP_community_, ANPP_sedge_, ANPP_graminoid_ and ANPP_forb_. Likewise, several previous studies demonstrated that increased precipitation did not affect species richness in alpine meadows [47], annual grasslands [48], infertile grasslands [38], and tallgrass prairies [49]. A low- and high-level increase in precipitation (20% and 40%, respectively) did not impact sedge aboveground production in an alpine meadow on the Tibetan Plateau [50]. No differences in graminoid coverage were detected among control, 20% and 40% increased precipitation in an alpine meadow [50], and control, 15% and 30% increased precipitation in an annual forb-dominated desert steppe [51]. A previous study also demonstrated that increased precipitation did not increase forb coverage [52]. First, except for MNTD and ANPP_sedge_, increased precipitation may only have indirect effects on species, phylogenetic α–diversity, and plant production, considering that only the effects of increased precipitation on MNTD and ANPP_sedge_ had significant correlations with Δ_IP__GSP. Second, although the increases in SM and GSP/AccT and the reduction in VPD under increased precipitation may be favorable for alpine plants, these probable positive effects may be dampened and even masked by the probable negative effect of the decrease in *T*_s_ under increased precipitation. Third, no effects of increased precipitation on soil pH, nitrogen, or phosphorus availability can explain the negligible effects of increased precipitation on species and phylogenetic α–diversity, ANPP_community_, ANPP_sedge_, ANPP_graminoid_, and ANPP_forb_. Fourth, the negligible effect of increased precipitation on ANPP_community_ can be related to that on species and phylogenetic α–diversity, ANPP_sedge_, ANPP_graminoid_, and ANPP_forb_. Fifth, the negligible effects of increased precipitation may also be related to the short-term duration (<7 years), because a greater increase or reduction in species and phylogenetic α–diversity, ANPP_community_, ANPP_sedge_, and ANPP_graminoid_ may occur along with an increasing duration of increased precipitation (Figure 5 and Figure S28).

### 3.3. Interactive Effects of Experimental Warming and Increased Precipitation

Several previous studies indicated that the main effect of warming and increased precipitation on species diversity and plant production may overestimate/underestimate the interactive effect of warming and increased precipitation on species diversity and plant production in grasslands [10,38], which was supported by this study. The negligible interactive effects of warming and increased precipitation on species richness and ANPP_community_ were also in line with some previous studies [53,54]. Although warming can elevate *T*_s_ and *T*_a_, it can also cause drying. Although increased precipitation can increase water availability, increased precipitation can also decrease *T*_s_. Moreover, both plant phyllosphere and soil microbial communities can also affect plant growth, α–diversity and community composition [55,56,57,58]. Climate change may cause plant phyllosphere and soil microbial communities to develop in a direction that is unfavorable to plant growth (e.g., the increase of plant pathogens) [55]. 

### 3.4. Stronger Inter-Annual Variations than Effects of Warming and Increased Precipitation

Inter-annual variations of species, phylogenetic diversity, and aboveground plant production were stronger than the effects of experimental warming or increased precipitation on these plant variables. This phenomenon was similar to some previous studies [21,35]. First, the maximum difference in growing season air temperature among years in 1963–2019 (2.7 °C) under natural conditions was greater than that (2.4 °C) under low-level warming. The maximum GSP difference among years in 2014–2019 (288.9 mm) and 1963–2019 (364.9 mm) under natural precipitation conditions was greater than the Δ_IP__GSP (45.0–176.7 mm) under increased precipitation. Second, aboveground plant production, plant species richness, and PD may be restored to their original levels with increasing warming duration (Figure 3 and Figure S16). Therefore, significant interannual variation can be related to the high inter-annual variations of environmental temperature and moisture and strong adaptation and resilience [59].

## 4. Materials and Methods

### 4.1. Study Area, Experimental Design and Microclimate Measurements

This experiment was conducted at an alpine grassland site (30°30′ N, 91°04′ E, 4313 m) from June 2014 to 2019. The mean annual temperature and mean annual precipitation were 1.96 °C and 476.36 mm in 1963–2019, respectively [17,21]. The dominant species are *Carex atrofusca*, *Stipa capillacea,* and *Kobresia pygmaea* [2,17]. Open-top chambers with 40 cm and 80 cm openings were used to simulate low- and high-magnitude warming, respectively. The opening sizes of these open-top chambers are hexagons with a side length of 60 cm. All the materials in the open top chambers are polythene. We also simulated two levels of increased precipitation (15% and 30%). Each treatment had three replicates. The nine treatments are control (CK), low- and high-level warming (LW and HW), low- and high-level increased precipitation (LP and HP), and their interactive effects (i.e., LW + LP, HW + LP, LW + HP, and HW + HP). We measured soil temperature (*T*_s_, 5 cm), soil moisture (SM, 10 cm), air temperature (*T*_a_, 15 cm), and relative humidity (RH, 15 cm) by HOBO weather stations (Onset Computer, Bourne, USA) during the growing season (June–September). We then calculated vapor pressure deficit (VPD) using measured *T*_a_ and RH and calculated the ratio of growing season precipitation to accumulated ≥5 °C daily air temperature (GSP/AccT). The effects of experimental warming and increased precipitation on *T*_s_, SM, *T*_a_, VPD, and/or GSP/AccT in 2014–2017 were reported in previous studies [10,35].

### 4.2. Community Investigation, ANPP Estimation, Soil Sampling, and Analyses

Plant community investigations for each plot in August 2014–2019 were conducted. The quadrat size of the community investigation was 50 cm × 50 cm. When we did the community investigation, the 50 cm × 50 cm quadrat was placed right in the center of each treatment plot. We recorded species coverage and height for each species within each of the twenty-seven plots. All the species names were artificially identified, and species coverage was artificially estimated. The 50 cm × 50 cm quadrat was divided into twenty-five 10 cm × 10 cm subquadrats to better estimate species coverage. The species height was measured using a steel tape with millimeter accuracy. There were three plant functional groups: sedge, graminoids, and forbs. Aboveground net primary production of sedge, graminoids, and forbs (i.e., ANPP_sedge_, ANPP_graminoid_ and ANPP_forb_) was estimated using observed coverage and height of plant function groups [5], respectively.

(1) $$ANPP_{sedge}=-0.77+0.93Coverage-0.24Height,R^{2}=0.95,p<0.001,n=120$$

(2) $$ANPP_{graminoid}=-2.25+1.03Coverage+0.06Height,R^{2}=0.95,p<0.001,n=120$$

(3) $$ANPP_{forb}=-1.00+0.50Coverage+1.71Height,R^{2}=0.91,p<0.001,n=120$$

The aboveground net primary production of plant community (ANPP_community_) was the sum of ANPP_sedge_, ANPP_graminoid_, and ANPP_forb_. Topsoil (0–10 cm) samples within all plots (areas outside the community investigation but inside each plot) were collected, sieved, and used to measure ammonium nitrogen (NH_4_^+^–N), nitrate nitrogen (NO_3_^−^–N), available phosphorus (AP), and pH in August 2014 and 2016–2018. Both NH_4_^+^–N and NO_3_^−^–N were measured on a LACHAT Quikchem Automated Ion Analyzer [60]. We measured soil pH using a soil pH meter [33,61,62]. We measured AP using the ammonium bicarbonate extraction molybdenum antimony resistance colorimetric method [18].

### 4.3. Statistical Analyses

We used the “specnumber” and “diversity” (vegan package) of R.4.1.2 software to calculate species α–diversity (SR—species richness; Shannon, Simpson, and Pielou).

(4) $$Shannon=-\sum_{i=1}^{n}p_{i}\times logp_{i}$$

(5) $$Simpson=1-\sum_{i=1}^{n}p_{i}^{2}$$

(6) $$Pielou=\frac{Shannon}{logSR}$$

where *p_i_* is the important value of each species within each sample. The important value of each plant species was the mean value of its relative coverage and relative height. We used the “vegdist” (Vegan package) of R.4.1.2 software to calculate species β–diversity (i.e., βBray, the dissimilarity indices of Bray–Curtis) between two treatments. We used “TPL” and “taxa.table” (Plantlist package) of R.4.1.2 software to obtain the taxonomy information (i.e., family, genus, and species) and then used Phylomatic software to generate a phylogenetic tree. We used the “pd”, “mntd”, and “comdistnt” (Picante package) of R.4.1.2 software to get phylogenetic α–diversity (PD: Faith’s phylogenetic diversity, i.e., the sum of the total phylogenetic branch length in one sample, and MNTD: mean nearest taxon distance for taxa in one sample) and phylogenetic β–diversity (i.e., βMNTD: beta mean nearest taxon distance) between two treatments. We used repeated-measures analysis of variance to estimate the main and interactive effects of experimental warming, increased precipitation, and measuring year on *T*_s_, SM, *T*_a_, VPD, AccT, GSP/AccT, NH_4_^+^–N, NO_3_^−^–N, AP, pH, SR, Shannon, Simpson, Pielou, PD, MNTD, ANPP_community_, ANPP_sedge_, ANPP_graminoid_, and ANPP_forb_. We then used Duncan multiple comparisons to examine the differences among the three experimental warming or increased precipitation levels. We used permutational multivariate analysis of variance (i.e., the “adonis2” of Vegan package) for the effects of experimental warming, increased precipitation, and measuring year on the βBray and βMNTD based on the R.4.1.2 software. The change magnitude of the abiotic and biotic variables caused by experimental warming (Δ_W_) or increased precipitation (Δ_IP_), and the response ratio of biotic variables to experimental warming (*R*_W_) or increased precipitation (*R*_IP_) were used as the effect size of experimental warming or increased precipitation for each year, respectively [63].

(7) $$\Delta_{W}\;\mathrm{or}\;\Delta_{\mathrm{IP}}=\bar{X_{t}}-\bar{X_{c}}$$

(8) $$R_{W}\;\mathrm{or}R_{\mathrm{IP}}=\bar{X_{t}}/\bar{X_{c}}$$

For the Δ_W_ and *R*_w_, $\bar{X_{t}}$ and $\bar{X_{c}}$ were concerned variables of the “LW” and “CK”, “HW” and “CK”, “LW + LP” and “LP”, “HW + LP” and “LP”, “LW + HP” and “HP”, or “HW + HP” and “HP”, respectively. For Δ_IP_ and *R*_IP_), $\bar{X_{t}}$ and $\bar{X_{c}}$ were concerned variables of the “LP” and “CK”, “HP” and “CK”, “LW + LP” and “LW”, “LW + HP” and “LW”, “HW + LP” and “HW”, or “HW + HP” and “HW”, respectively. We used “varpart” (Vegan package) of R.4.1.2 software to partition the variation of the plant α– and β–diversity and aboveground net plant production by four explanatory matrices of biotic and/or abiotic variables. All the statistical analyses were examined at *p* < 0.05.

## 5. Conclusions

In summary, warming and increased precipitation were not always favorable for aboveground net primary production, species and phylogenetic α–diversity and community composition, which were related to the duration and magnitude of warming and increased precipitation, respectively. Species and phylogenetic α–diversity and composition did not have entirely uniform responses to warming and increased precipitation, and they had different correlations with aboveground net primary production. The combination of species and phylogenetic α–diversity and composition can better reflect the effects of climate warming and increased precipitation on plant α–diversity and community composition, and also better explain the variation of aboveground net primary production under controlled warming and increased precipitation conditions. Aboveground net primary production, species and phylogenetic α–diversity, and community composition had obvious inter-annual variations, and their variations were greater than their responses to warming and increased precipitation. Sedge, graminoid, and forb aboveground net primary production had different responses to warming. 

The scientific findings of this study can provide some guidance for the conservation of plant diversity and the development of animal husbandry. First, compared to the regions with lower warming, more attention should be paid to the conservation of plant diversity in regions with greater warming. Second, in the context of climate change, biodiversity conservation policies should take into consideration both species and phylogenetic diversity. Third, we may pay more attention to the large inter-annual changes of ANPP and plant diversity than the effects of climate change on ANPP and plant diversity in terms of the stability of livestock and herders. Fourth, we need to be aware that the actual effects of warmer-wetter climate change trends on ANPP and plant diversity may be lower than their expected effects.

## Figures and Tables

**Table 1:** Repeated-measures analysis of variance was used to estimate the main and interactive effects of experimental warming (W), increased precipitation (IP), and measuring year (Y) on aboveground net primary production at community level (ANPP_community_), sedge ANPP (ANPP_sedge_), graminoid ANPP (ANPP_graminoid_), forb ANPP (ANPP_forb_), species richness (SR), Shannon, Simpson, Pielou, and Faith’s phylogenetic diversity (PD), and mean nearest taxon distance (MNTD).

Model	ANPP_community_	ANPP_sedge_	ANPP_graminoid_	ANPP_forb_	SR	Shannon	Simpson	Pielou	PD	MNTD
Warming (W)	1.00	4.00 *	1.00	4.00 *	15.00 ***	5.00 *	2.00	1.00	12.00 ***	4.00 *
Precipitation (IP)	3.00 ^+^	2.00	1.00	1.00	0.00	0.00	0.00	0.00	0.00	1.00
Year (Y)	43.00 ***	7.00 ***	18.00 ***	11.00 ***	38.00 ***	25.00 ***	16.00 ***	4.00 **	19.00 ***	23.00 ***
W × IP	0.00	1.00	1.00	1.00	1.00	1.00	1.00	1.00	0.00	1.00
W × Y	2.00	1.00	1.00	6.00 ***	4.00 ***	3.00 **	2.00 ^+^	1.00	4.00 ***	2.00 *
IP × Y	3.00 **	3.00 **	1.00	0.00	1.00	1.00	1.00	1.00	1.00	1.00
W × IP × Y	1.00	1.00	1.00	0.00	1.00	2.00 ^+^	1.00	1.00	1.00	2.00 *

^+^, *, **, and *** indicate *p* < 0.10, *p* < 0.05, *p* < 0.01, and *p* < 0.001, respectively.

**Table 2:** Permutational multivariate analysis of variance was used to estimate the main and interactive effects of experimental warming (W), increased precipitation (IP), and measuring year (Y) on species composition and phylogenetic composition.

Model	Species Composition	Phylogenetic Structure
Warming (W)	5.82 **	10.78 **
Precipitation (IP)	1.96 *	6.21 **
Year (Y)	29.67 **	52.89 **
W × IP	2.89 **	7.19 **
W × Y	1.91 **	2.08 *
IP × Y	0.79	−0.16
W × IP × Y	0.61	0.30

* and ** indicate *p* < 0.05 and *p* < 0.01, respectively.

## Data Availability

Request from the corresponding authors.

## Supplementary Materials

The following supporting information can be downloaded at: https://www.mdpi.com/article/10.3390/plants12173017/s1, Figure S1: Comparison of species α–diversity (SR: species richness; Shannon, Simpson and Pielou) among the nine experimental treatments in 2014, 2015, 2016, 2017, 2018, 2019 and 2014–2019, respectively. Different letters indicate significant difference at *p* < 0.05; Figure S2: Comparison of phylogenetic α–diversity (PD: Faith’s phylogenetic diversity; MNTD: mean nearest taxon distance) among the nine experimental treatments in 2014, 2015, 2016, 2017, 2018, 2019 and 2014–2019, respectively. Different letters indicate significant difference at *p* < 0.05; Figure S3: Comparison of aboveground net primary production at community, sedge, graminoid and forb levels (ANPP_community_, ANPP_sedge_, ANPP_graminoid_ and ANPP_forb_) among the nine experimental treatments in 2014, 2015, 2016, 2017, 2018, 2019 and 2014–2019, respectively. Different letters indicate significant difference at *p* < 0.05; Figure S4: Comparison of the low- and high-level experimental warming-induced dissimilarity of species composition (βBray_W_) and phylogenetic composition (βMNTD_W_) under the no, low- and high-level increased precipitation conditions in 2014, 2015, 2016, 2017, 2018, 2019 and 2014–2019, respectively. *, ** and *** indicate significant difference at *p* < 0.05, *p* < 0.01 and *p* < 0.001, respectively; Figure S5: Comparison of the low- and high-level increased precipitation-induced dissimilarity of species composition (βBray_IP_) and phylogenetic composition (βMNTD_IP_) under the no, low- and high-level experimental warming conditions in 2014, 2015, 2016, 2017, 2018, 2019 and 2014–2019, respectively. *, ** and *** indicate significant difference at *p* < 0.05, *p* < 0.01 and *p* < 0.001, respectively; Figure S6: Comparison of average soil temperature (*T*_s_), soil moisture (SM), air temperature (*T*_a_), vapor pressure deficit (VPD), accumulated ≥5 °C daily air temperature (AccT), and ratio of growing season precipitation to AccT (GSP/AccT) in 2014–2019 among the nine experimental treatments. Different letters indicate significant difference at *p* < 0.05; Figure S7: Relationships (a) between the decreased magnitude of soil moisture caused by experimental warming (ΔSM) and increased magnitude of air temperature caused by experimental warming (Δ_W__*T*_a_); (b) between ΔSM and increased magnitude of soil temperature caused by experimental warming (Δ_W__*T*_s_); (c) between the increased magnitude of vapor pressure deficit caused by experimental warming (ΔVPD) and Δ_W__*T*_a_; (d) between ΔVPD and Δ_W__*T*_s_; and (e) between the decreased magnitude of the ratio of growing season precipitation to accumulated ≥5 °C daily air temperature caused by experimental warming (ΔGSP/AccT) and Δ_W__*T*_a_; Figure S8: Comparison of ammonium nitrogen (NH_4_^+^-N), nitrate nitrogen (NO_3_^−^-N), available phosphorus (AP) and pH among the nine experimental treatments in 2014, 2016, 2017, 2018 and 2014–2018, respectively. Different letters indicate significant difference at *p* < 0.05; Figure S9: Relationships (a) between the change magnitude of species richness caused by experimental warming (Δ_W__SR) and increased magnitude of air temperature caused by experimental warming (Δ_W__*T*_a_); (b) between the change magnitude of Pielou caused by experimental warming (Δ_W__Pielou) and Δ_W__*T*_a_; (c) between the change magnitude of Faith’s phylogenetic diversity caused by experimental warming (Δ_W__PD) and Δ_W__*T*_a_; (d) between the change magnitude of mean nearest taxon distance caused by experimental warming (Δ_W__MNTD) and Δ_W__*T*_a_; (e) between the change magnitude of community aboveground net primary production caused by experimental warming (Δ_W__ANPP_community_) and Δ_W__*T*_a_; (f) between the change magnitude of sedge aboveground net primary production caused by experimental warming (Δ_W__ANPP_sedge_) and Δ_W__*T*_a_; (g) between the change magnitude of graminoid aboveground net primary production caused by experimental warming (Δ_W__ANPP_graminoid_) and Δ_W__*T*_a_; and between the change magnitude of forb aboveground net primary production caused by experimental warming (Δ_W__ANPP_forb_) and Δ_W__*T*_a_; Figure S10: Relationships (a) between the response ratio of species richness to experimental warming (*R*_W__SR) and increased magnitude of soil temperature caused by experimental warming (Δ_W__*T*_s_); (b) between the response ratio of Pielou to experimental warming (*R*_W__Pielou) and Δ_W__*T*_s_; (c) between the response ratio of Faith’s phylogenetic diversity to experimental warming (*R*_W__PD) and Δ_W__*T*_s_; (d) between species β–diversity (βBray_w_) and Δ_W__*T*_s_; (e) between the response ratio of community aboveground net primary production to experimental warming (*R*_W__ANPP_community_) and Δ_W__*T*_s_; (f) between the response ratio of graminoid aboveground net primary production to experimental warming (*R*_W__ANPP_graminoid_) and Δ_W__*T*_s_; and (g) between the response ratio of forb aboveground net primary production to experimental warming (*R*_W__ANPP_forb_) and Δ_W__*T*_s_; Figure S11: Relationships (a) between the change magnitude of species richness caused by experimental warming (Δ_W__SR) and increased magnitude of soil temperature caused by experimental warming (Δ_W__*T*_s_); (b) between the change magnitude of Pielou caused by experimental warming (Δ_W__Pielou) and Δ_W__*T*_s_; (c) between the change magnitude of Faith’s phylogenetic diversity caused by experimental warming (Δ_W__PD) and Δ_W__*T*_s_; (d) between the change magnitude of community aboveground net primary production caused by experimental warming (Δ_W__ANPP_community_) and Δ_W__*T*_s_; (e) between the change magnitude of graminoid aboveground net primary production caused by experimental warming (Δ_W__ANPP_graminoid_) and Δ_W__*T*_s_; and (f) between the change magnitude of forb aboveground net primary production caused by experimental warming (Δ_W__ANPP_forb_) and Δ_W__*T*_s_; Figure S12: Relationships (a) between the response ratio of species richness to experimental warming (*R*_W__SR) and decreased magnitude of soil moisture caused by experimental warming (Δ_W__SM); (b) between the response ratio of Shannon to experimental warming (*R*_W__Shannon) and Δ_W__SM; (c) between the response ratio of Faith’s phylogenetic diversity to experimental warming (*R*_W__PD) and Δ_W__SM; (d) between the response ratio of community aboveground net primary production to experimental warming (*R*_W__ANPP_community_) and Δ_W__SM; (e) between the response ratio of forb aboveground net primary production to experimental warming (*R*_W__ANPP_forb_) and Δ_W__SM; (f) between the *R*_W__SR and increased magnitude of vapor pressure deficit caused by experimental warming (Δ_W__VPD); (g) between the *R*_W__PD and Δ_W__VPD; (h) between the *R*_W__ANPP_community_ and Δ_W__VPD; (i) between the response ratio of graminoid aboveground net primary production to experimental warming (*R*_W__ANPP_graminoid_) and Δ_W__VPD; (j) between the *R*_W__ANPP_forb_ and Δ_W__VPD; (k) between the *R*_W__SR and decreased magnitude of accumulated ≥5 °C daily air temperature caused by experimental warming (Δ_W__GSP/AccT); (l) between the *R*_W__PD and Δ_W__GSP/AccT; (m) between the response ratio of sedge aboveground net primary production to experimental warming (*R*_W__ANPP_sedge_) and Δ_W__GSP/AccT; (n) between the *R*_W__ANPP_graminoid_ and Δ_W__GSP/AccT; (o) between the *R*_W__ANPP_forb_ and Δ_W__GSP/AccT; (p) between the species β–diversity of warming versus no-warming conditions (βBray_w_) and Δ_W__GSP/AccT; and (q) between the phylogenetic β–diversity of warming versus no-warming conditions (βMNTD_w_) and Δ_W__GSP/AccT; Figure S13: Relationships (a) between change magnitude of species richness caused by experimental warming (Δ_W__SR) and increased magnitude of vapor pressure deficit caused by experimental warming (Δ_W__VPD); (b) between the change magnitude of Faith’s phylogenetic diversity caused by experimental warming (Δ_W__PD) and Δ_W__VPD; (c) between the change magnitude of community aboveground net primary production caused by experimental warming (Δ_W__ANPP_community_) and Δ_W__VPD; (d) between the change magnitude of forb aboveground net primary production caused by experimental warming (Δ_W__ANPP_forb_) and Δ_W__VPD; (e) between the Δ_W__SR and decreased magnitude of accumulated ≥5 °C daily air temperature caused by experimental warming (Δ_W__GSP/AccT); (f) between Δ_W__PD and Δ_W__GSP/AccT; (g) between the change magnitude of graminoid aboveground net primary production caused by experimental warming (Δ_W__ANPP_graminoid_) and ΔGSP/AccT; (h) between the Δ_W__ANPP_forb_ and Δ_W__GSP/AccT; (i) between the Δ_W__SR and the decreased magnitude of soil moisture caused by experimental warming (ΔSM); (j) between the Δ_W__PD and Δ_W__SM; (k) between the Δ_W__ANPP_community_ and Δ_W__SM; (l) between the Δ_W__ANPP_forb_ and Δ_W__SM; (m) between the change magnitude of Shannon caused by experimental warming (Δ_W__Shannon) and Δ_W__SM; (n) between the change magnitude of sedge aboveground net primary production caused by experimental warming (Δ_W__ANPP_sedge_) and Δ_W__SM; (o) between the Δ_W__ANPP_graminoid_ and Δ_W__SM; and (p) between the change magnitude of mean nearest taxon distance caused by experimental warming (Δ_W__MNTD) and ΔGSP/AccT; Figure S14: Relationships (a) between the response ratio of species richness to experimental warming (*R*_W__SR) and change magnitude of soil pH caused by experimental warming (Δ_W__pH); (b) between the response ratio of Shannon to experimental warming (*R*_W__Shannon) and Δ_W__pH; (c) between the response ratio of Simpson to experimental warming (*R*_W__Simpson) and Δ_W__pH; (d) between the response ratio of Faith’s phylogenetic diversity to experimental warming (*R*_W__PD) and Δ_W__pH; (e) between the phylogenetic β–diversity of warming versus no-warming conditions (βMNTD_w_) and Δ_W__pH; (f) between the *R*_W__SR and the change magnitude of nitrate nitrogen (Δ_W__NO_3_^−^-N) caused by experimental warming; (g) between the *R*_W__Shannon and Δ_W__NO_3_^−^-N; (h) between the *R*_W__Simpson and Δ_W__NO_3_^−^-N; (i) between the *R*_W__PD and Δ_W__NO_3_^−^-N; (j) between the response ratio of mean nearest taxon distance to experimental warming (*R*_W__MNTD) and Δ_W__NO_3_^−^-N; (k) between the response ratio of community aboveground net primary production to experimental warming (*R*_W__ANPP_community_) and Δ_W__NO_3_^−^-N; (l) between the response ratio of graminoid aboveground net primary production to experimental warming (*R*_W__ANPP_graminoid_) and Δ_W__NO_3_^−^-N; (m) between species β–diversity of warming versus no-warming conditions (βBray_w_) and Δ_W__NO_3_^−^-N; (n) between the βMNTD_w_ and Δ_W__NO_3_^−^-N; (o) between the response ratio of forb aboveground net primary production to experimental warming (*R*_W__ANPP_forb_) and the change magnitude of ammonium nitrogen (Δ_W__NH_4_^+^-N) caused by experimental warming; (p) between the *R*_W__ANPP_community_ and the change magnitude of available phosphorus (Δ_W__AP) caused by experimental warming; and (q) between the response ratio of sedge aboveground net primary production to experimental warming (*R*_W__ANPP_sedge_) and Δ_W__AP; Figure S15: Relationships (a) between the change magnitude of species richness caused by experimental warming (Δ_W__SR) and change magnitude of soil pH caused by experimental warming (Δ_W__pH); (b) between the change magnitude of Shannon caused by experimental warming (Δ_W__Shannon) and Δ_W__pH; (c) between the change magnitude of Simpson caused by experimental warming (Δ_W__Simpson) and Δ_W__pH; (d) between the change magnitude of Faith’s phylogenetic diversity caused by experimental warming (Δ_W__PD) and Δ_W__pH; (e) between the Δ_W__SR and the change magnitude of nitrate nitrogen (Δ_W__NO_3_^−^-N) caused by experimental warming; (f) between the Δ_W__Shannon and Δ_W__NO_3_^−^-N; (g) between the Δ_W__Simpson and Δ_W__NO_3_^−^-N; (h) between the Δ_W__PD and Δ_W__NO_3_^−^-N; (i) between the change magnitude of mean nearest taxon distance to experimental warming (Δ_W__MNTD) and Δ_W__NO_3_^−^-N; (j) between the change magnitude of sedge aboveground net primary production caused by experimental warming (Δ_W__ANPP_sedge_) and Δ_W__NO_3_^−^-N; (k) between the change magnitude of graminoid aboveground net primary production caused by experimental warming (*R*_W__ANPP_graminoid_) and the change magnitude of ammonium nitrogen (Δ_W__NH_4_^+^-N) caused by experimental warming; (l) between the change magnitude of forb aboveground net primary production caused by experimental warming (*R*_W__ANPP_forb_) and Δ_W__NH_4_^+^-N; and (m) between the *R*_W__ANPP_sedge_ and the change magnitude of available phosphorus (Δ_W__AP) caused by experimental warming; Figure S16: Relationships (a) between the change magnitude of species richness caused by experimental warming (Δ_W__SR) and warming duration; (b) between the change magnitude of Shannon caused by experimental warming (Δ_W__Shannon) and warming duration; (c) between the change magnitude of Simpson caused by experimental warming (Δ_W__Simpson) and warming duration; (d) between the change magnitude of Faith’s phylogenetic diversity caused by experimental warming (Δ_W__PD) and warming duration; (e) between the change magnitude of mean nearest taxon distance to experimental warming (Δ_W__MNTD) and warming duration; (f) between the change magnitude of sedge aboveground net primary production caused by experimental warming (Δ_W__ANPP_sedge_) and warming duration; and (g) between the change magnitude of forb aboveground net primary production caused by experimental warming (Δ_W__ANPP_forb_) and warming duration; Figure S17: Venn plots of variation partitioning analysis, showing the shared and exclusive effects of warming duration, Δ_W__T (i.e., the change magnitude of air and/or soil temperature caused by experimental warming), Δ_W__W (i.e., the change magnitude of soil moisture and/or vapor pressure deficit caused by experimental warming) and Δ_W__GSP/AccT (i.e., the change magnitude of the ratio of growing season precipitation to accumulated ≥5 °C daily air temperature caused by experimental warming) on (a) the response ratio of species richness to experimental warming (*R*_W__SR), (b) the response ratio of Shannon to experimental warming (*R*_W__Shannon), (c) the response ratio of Simpson to experimental warming (*R*_W__Simpson), (d) the response ratio of Pielou to experimental warming (*R*_W__Pielou), (e) the response ratio of Faith’s phylogenetic diversity to experimental warming (*R*_W__PD), (f) the response ratio of mean nearest taxon distance to experimental warming (*R*_W__MNTD), (g) species β–diversity (βBray_w_) between the warming and non-warming conditions, (h) phylogenetic β–diversity (βMNTD_w_) between the warming and non-warming conditions, (i) the response ratio of community aboveground net primary production to experimental warming (*R*_W__ANPP_community_), (j) the response ratio of sedge aboveground net primary production to experimental warming (*R*_W__ANPP_sedge_), (k) the response ratio of graminoid aboveground net primary production to experimental warming (*R*_W__ANPP_graminoid_), and (l) the response ratio of forb aboveground net primary production to experimental warming (*R*_W__ANPP_forb_); Figure S18: Venn plots of variation partitioning analysis, showing the shared and exclusive effects of warming duration, Δ_W__T (i.e., the change magnitude of air and/or soil temperature caused by experimental warming), Δ_W__W (i.e., the change magnitude of soil moisture and/or vapor pressure deficit caused by experimental warming) and Δ_W__GSP/AccT (i.e., the change magnitude of the ratio of growing season precipitation to accumulated ≥5 °C daily air temperature caused by experimental warming) on (a) the change magnitude of species richness caused by experimental warming (Δ_W__SR), (b) the change magnitude of Shannon caused by experimental warming (Δ_W__Shannon), (c) the change magnitude of Simpson caused by experimental warming (Δ_W__Simpson), (d) the change magnitude of Pielou caused by experimental warming (Δ_W__Pielou), (e) the change magnitude of Faith’s phylogenetic diversity caused by experimental warming (Δ_W__PD), (f) the change magnitude of mean nearest taxon distance caused by experimental warming (Δ_W__MNTD), (g) the change magnitude of community aboveground net primary production caused by experimental warming (Δ_W__ANPP_community_), (h) the change magnitude of sedge aboveground net primary production caused by experimental warming (Δ_W__ANPP_sedge_), (i) the change magnitude of graminoid aboveground net primary production caused by experimental warming (Δ_W__ANPP_graminoid_), and (j) the change magnitude of forb aboveground net primary production caused by experimental warming (Δ_W__ANPP_forb_); Figure S19: Venn plots of variation partitioning analysis, showing the shared and exclusive effects of warming duration, Δ_W__N (i.e., the change magnitude of ammonium nitrogen and/or nitrate nitrogen caused by experimental warming), Δ_W__AP (i.e., the change magnitude of soil available phosphorus caused by experimental warming) and Δ_W__pH (i.e., the change magnitude of soil pH caused by experimental warming) on (a) the response ratio of species richness to experimental warming (*R*_W__SR), (b) the response ratio of Shannon to experimental warming (*R*_W__Shannon), (c) the response ratio of Simpson to experimental warming (*R*_W__Simpson), (d) the response ratio of Pielou to experimental warming (*R*_W__Pielou), (e) the response ratio of Faith’s phylogenetic diversity to experimental warming (*R*_W__PD), (f) the response ratio of mean nearest taxon distance to experimental warming (*R*_W__MNTD), (g) species β–diversity (βBray_w_) between the warming and non-warming conditions, (h) phylogenetic β–diversity (βMNTD_w_) between the warming and non-warming conditions, (i) the response ratio of community aboveground net primary production to experimental warming (*R*_W__ANPP_community_), (j) the response ratio of sedge aboveground net primary production to experimental warming (*R*_W__ANPP_sedge_), (k) the response ratio of graminoid aboveground net primary production to experimental warming (*R*_W__ANPP_graminoid_), and (l) the response ratio of forb aboveground net primary production to experimental warming (*R*_W__ANPP_forb_); Figure S20: Venn plots of variation partitioning analysis, showing the shared and exclusive effects of warming duration, Δ_W__N (i.e., the change magnitude of ammonium nitrogen and/or nitrate nitrogen caused by experimental warming), Δ_W__AP (i.e., the change magnitude of soil available phosphorus caused by experimental warming) and Δ_W__pH (i.e., the change magnitude of soil pH caused by experimental warming) on (a) the change magnitude of species richness caused by experimental warming (Δ_W__SR), (b) the change magnitude of Shannon caused by experimental warming (Δ_W__Shannon), (c) the change magnitude of Simpson caused by experimental warming (Δ_W__Simpson), (d) the change magnitude of Pielou caused by experimental warming (Δ_W__Pielou), (e) the change magnitude of Faith’s phylogenetic diversity caused by experimental warming (Δ_W__PD), (f) the change magnitude of mean nearest taxon distance caused by experimental warming (Δ_W__MNTD), (g) the change magnitude of community aboveground net primary production caused by experimental warming (Δ_W__ANPP_community_), (h) the change magnitude of sedge aboveground net primary production caused by experimental warming (Δ_W__ANPP_sedge_), (i) the change magnitude of graminoid aboveground net primary production caused by experimental warming (Δ_W__ANPP_graminoid_), and (j) the change magnitude of forb aboveground net primary production caused by experimental warming (Δ_W__ANPP_forb_); Figure S21: Venn plots of variation partitioning analysis, showing the shared and exclusive effects of warming duration, Δ_W__T&W (i.e., the change magnitude of air and/or soil temperature, soil moisture, vapor pressure deficit and/or the ratio of growing season precipitation to accumulated ≥5 °C daily air temperature caused by experimental warming), Δ_W__N&P (i.e., the change magnitude of ammonium nitrogen, nitrate nitrogen, and/or available phosphorus caused by experimental warming) and Δ_W__pH (i.e., the change magnitude of soil pH caused by experimental warming) on (a) the change magnitude of species richness caused by experimental warming (Δ_W__SR), (b) the change magnitude of Shannon caused by experimental warming (Δ_W__Shannon), (c) the change magnitude of Simpson caused by experimental warming (Δ_W__Simpson), (d) the change magnitude of Pielou caused by experimental warming (Δ_W__Pielou), (e) the change magnitude of Faith’s phylogenetic diversity caused by experimental warming (Δ_W__PD), (f) the change magnitude of mean nearest taxon distance caused by experimental warming (Δ_W__MNTD), (g) the change magnitude of community aboveground net primary production caused by experimental warming (Δ_W__ANPP_community_), (h) the change magnitude of sedge aboveground net primary production caused by experimental warming (Δ_W__ANPP_sedge_), (i) the change magnitude of graminoid aboveground net primary production caused by experimental warming (Δ_W__ANPP_graminoid_), and (j) the change magnitude of forb aboveground net primary production caused by experimental warming (Δ_W__ANPP_forb_); Figure S22: Relationships (a) between the response ratio of community aboveground net primary production to experimental warming (*R*_W__ANPP_community_) and the response ratio of species richness to experimental warming (*R*_W__SR); (b) between the *R*_W__ANPP_community_ and the response ratio of Pielou to experimental warming (*R*_W__Pielou); (c) between the *R*_W__ANPP_community_ and the response ratio of Faith’s phylogenetic diversity to experimental warming (*R*_W__PD); (d) the change magnitude of community aboveground net primary production caused by experimental warming (Δ_W__ANPP_community_) and the change magnitude of Simpson caused by experimental warming (Δ_W__Simpson); (e) between the Δ_W__ANPP_community_ and the change magnitude of Pielou caused by experimental warming (Δ_W__Pielou); and (f) between the Δ_W__ANPP_community_ and the phylogenetic β–diversity of warming versus no-warming conditions (βMNTD_w_); Figure S23: Venn plots of variation partitioning analysis, showing the shared and exclusive effects of (a) *R*_W__α-species (i.e., the response ratio of species richness, Shannon, Simpson and/or Pielou to experimental warming), *R*_W__α-phylo (i.e., the response ratio of Faith’s phylogenetic diversity and/or mean nearest taxon distance to experimental warming), βBray (i.e., species β–diversity of warming versus non-warming conditions) and βMNTD (i.e., phylogenetic β–diversity of warming versus non-warming conditions) on the response ratio of community aboveground net primary production to experimental warming (*R*_W__ANPP_community_); and (b) Δ_W__α-species (i.e., the change magnitude of species richness, Shannon, Simpson and/or Pielou caused by experimental warming), Δ_W__α-phylo (i.e., the change magnitude of Faith’s phylogenetic diversity and/or mean nearest taxon distance caused by experimental warming), βBray (i.e., species β–diversity of warming versus non-warming conditions) and βMNTD (i.e., phylogenetic β–diversity of warming versus non-warming conditions) on the change magnitude of community aboveground net primary production caused by experimental warming (Δ_W__ANPP_community_); Figure S24: Relationships (a) between the response ratio of community aboveground net primary production to increased precipitation (*R*_IP__ANPP_community_) and the change magnitude of vapor pressure deficit (Δ_IP__VPD); (b) between the change magnitude of community aboveground net primary production caused by increased precipitation (Δ_IP__ANPP_community_) and Δ_IP__VPD; (c) between the Δ_IP__ANPP_community_ and the change magnitude of the ratio of growing season precipitation to accumulated ≥5 °C daily air temperature (Δ_IP__GSP/AccT); (d) between the change magnitude of graminoid aboveground net primary production caused by increased precipitation (Δ_IP__ANPP_graminoid_) and Δ_IP__GSP/AccT; (e) between the change magnitude of species richness caused by increased precipitation (Δ_IP__SR) and the change magnitude of soil moisture (Δ_IP__SM); (f) between the change magnitude of Shannon caused by increased precipitation (Δ_IP__Shannon) and Δ_IP__SM; (g) between the change magnitude of mean nearest taxon distance caused by increased precipitation (Δ_IP__MNTD) and Δ_IP__SM; (h) between the change magnitude of sedge aboveground net primary production caused by increased precipitation (Δ_IP__ANPP_sedge_) and Δ_IP__SM; (i) between the species β–diversity of the increased versus no-increased precipitation conditions (βBray_IP_) and Δ_IP__SM; (j) between the response ratio of Shannon to increased precipitation (*R*_IP__Shannon) and Δ_IP__SM; and (k) between the response ratio of mean nearest taxon distance to increased precipitation (*R*_IP__MNTD) and Δ_IP__SM; Figure S25: Relationships (a) the phylogenetic β–diversity of the increased versus no-increased precipitation conditions (βMNTD_IP_) and the change magnitude of air temperature caused by increased precipitation (Δ_IP__*T*_a_); (b) between the response ratio of sedge aboveground net primary production to increased precipitation (*R*_IP__ANPP_sedge_) and Δ_IP__*T*_a_; (c) between the change magnitude of sedge aboveground net primary production caused by increased precipitation (Δ_IP__ANPP_sedge_) and Δ_IP__*T*_a_; (d) between the response ratio of Pielou to increased precipitation (*R*_IP__Pielou) and the change magnitude of soil temperature caused by increased precipitation (Δ_IP__*T*_s_); (e) between the response ratio of community aboveground net primary production to increased precipitation (*R*_IP__ANPP_community_) and the Δ_IP__*T*_s_; (f) between the response ratio of forb aboveground net primary production to increased precipitation (*R*_IP__ANPP_forb_) and the Δ_IP__*T*_s_; (g) between the change magnitude of graminoid aboveground net primary production caused by increased precipitation (Δ_IP__ ANPP_graminoid_) and Δ_IP__*T*_s_; (h) between the change magnitude of community aboveground net primary production caused by increased precipitation (Δ_IP__ ANPP_community_) and Δ_IP__*T*_s_; and (i) between the change magnitude of forb aboveground net primary production caused by increased precipitation (Δ_IP__ ANPP_forb_) and Δ_IP__*T*_s_; Figure S26: Relationships (a) between the response ratio of species richness to increased precipitation (*R*_IP__SR) and the change magnitude of available phosphorus caused by increased precipitation (Δ_IP__AP); (b) between the response ratio of Shannon to increased precipitation (*R*_IP__Shannon) and Δ_IP__AP; (c) between the response ratio of Simpson to increased precipitation (*R*_IP__Simpson) and Δ_IP__AP; (d) between the response ratio of Pielou to increased precipitation (*R*_IP__Pielou) and Δ_IP__AP; (e) between the response ratio of phylogenetic diversity to increased precipitation (*R*_IP__PD) and Δ_IP__AP; (f) between the response ratio of mean nearest taxon distance to increased precipitation (*R*_IP__MNTD) and Δ_IP__AP; (g) between the species β–diversity of the increased versus no-increased precipitation conditions (βBray_IP_) and Δ_IP__AP; (h) between the change magnitude of species richness caused by increased precipitation (Δ_IP__SR) and Δ_IP__AP; (i) between the change magnitude of Shannon caused by increased precipitation (Δ_IP__Shannon) and Δ_IP__AP; (j) between the change magnitude of Simpson caused by increased precipitation (Δ_IP__Simpson) and Δ_IP__AP; (k) between the change magnitude of Pielou caused by increased precipitation (Δ_IP__Pielou) and Δ_IP__AP; (l) between the change magnitude of phylogenetic diversity caused by increased precipitation (Δ_IP__PD) and Δ_IP__AP; (m) between the change magnitude of mean nearest taxon distance caused by increased precipitation (Δ_IP__MNTD) and Δ_IP__AP; and (n) between the phylogenetic β–diversity of the increased versus no-increased precipitation conditions (βMNTD_IP_) and Δ_IP__AP; Figure S27: Relationships (a) between the change magnitude of mean nearest taxon distance caused by increased precipitation (Δ_IP__MNTD) and the change magnitude of ammonium nitrogen caused by increased precipitation (Δ_IP__NH_4_^+^-N); and (b) between the change magnitude of sedge aboveground net primary production caused by increased precipitation (Δ_IP__ANPP_sedge_) and the change magnitude of nitrate nitrogen caused by increased precipitation (Δ_IP__NO_3_^−^-N); Figure S28: Relationships (a) between the change magnitude of community aboveground net primary production caused by increased precipitation (Δ_IP__ANPP_community_) and increased precipitation duration; (b) between the change magnitude of sedge aboveground net primary production caused by increased precipitation (Δ_IP__ANPP_sedge_) and increased precipitation duration; (c) between the change magnitude of graminoid aboveground net primary production caused by increased precipitation (Δ_IP__ANPP_graminoid_) and increased precipitation duration; and (d) between the change magnitude of Pielou caused by increased precipitation (Δ_IP__Pielou) and increased precipitation duration; Figure S29: Venn plots of variation partitioning analysis, showing the shared and exclusive effects of increased precipitation duration, Δ_IP__T (i.e., the change magnitude of air and/or soil temperature caused by increased precipitation), Δ_IP__W (i.e., the change magnitude of growing season precipitation, soil moisture and/or vapor pressure deficit caused by increased precipitation) and Δ_IP__GSP/AccT (i.e., the change magnitude of the ratio of growing season precipitation to accumulated ≥5 °C daily air temperature caused by increased precipitation) on (a) the response ratio of species richness to increased precipitation (*R*_IP__SR), (b) the response ratio of Shannon to increased precipitation (*R*_IP__Shannon), (c) the response ratio of Simpson to increased precipitation (*R*_IP__Simpson), (d) the response ratio of Pielou to increased precipitation (*R*_IP__Pielou), (e) the response ratio of Faith’s phylogenetic diversity to increased precipitation (*R*_IP__PD), (f) the response ratio of mean nearest taxon distance to increased precipitation (*R*_IP__MNTD), (g) species β–diversity (βBray_IP_) between the increased precipitation and non-increased precipitation conditions, (h) phylogenetic β–diversity (βMNTD_IP_) between the increased precipitation and non-increased precipitation conditions, (i) the response ratio of community aboveground net primary production to increased precipitation (*R*_IP__ANPP_community_), (j) the response ratio of sedge aboveground net primary production to increased precipitation (*R*_IP__ANPP_sedge_), (k) the response ratio of graminoid aboveground net primary production to increased precipitation (*R*_IP__ANPP_graminoid_), and (l) the response ratio of forb aboveground net primary production to increased precipitation (*R*_IP__ANPP_forb_); Figure S30: Venn plots of variation partitioning analysis, showing the shared and exclusive effects of increased precipitation duration, Δ_IP__N (i.e., the change magnitude of ammonium nitrogen and/or nitrate nitrogen caused by increased precipitation), Δ_IP__AP (i.e., the change magnitude of soil available phosphorus caused by increased precipitation) and Δ_IP__pH (i.e., the change magnitude of soil pH caused by increased precipitation) on (a) the response ratio of species richness to increased precipitation (*R*_IP__SR), (b) the response ratio of Shannon to increased precipitation (*R*_IP__Shannon), (c) the response ratio of Simpson to increased precipitation (*R*_IP__Simpson), (d) the response ratio of Pielou to increased precipitation (*R*_IP__Pielou), (e) the response ratio of Faith’s phylogenetic diversity to increased precipitation (*R*_IP__PD), (f) the response ratio of mean nearest taxon distance to increased precipitation (*R*_IP__MNTD), (g) species β–diversity (βBray_IP_) between the increased precipitation and non-increased precipitation conditions, (h) phylogenetic β–diversity (βMNTD_IP_) between the increased precipitation and non-increased precipitation conditions, (i) the response ratio of community aboveground net primary production to increased precipitation (*R*_IP__ANPP_community_), (j) the response ratio of sedge aboveground net primary production to increased precipitation (*R*_IP__ANPP_sedge_), (k) the response ratio of graminoid aboveground net primary production to increased precipitation (*R*_IP__ANPP_graminoid_), and (l) the response ratio of forb aboveground net primary production to increased precipitation (*R*_IP__ANPP_forb_); Figure S31: Venn plots of variation partitioning analysis, showing the shared and exclusive effects of increased precipitation duration, Δ_IP__T (i.e., the change magnitude of air and/or soil temperature caused by increased precipitation), Δ_IP__W (i.e., the change magnitude of growing season precipitation, soil moisture and/or vapor pressure deficit caused by increased precipitation) and Δ_IP__GSP/AccT (i.e., the change magnitude of the ratio of growing season precipitation to accumulated ≥5 °C daily air temperature caused by increased precipitation) on (a) the change magnitude of species richness caused by increased precipitation (Δ_IP__SR), (b) the change magnitude of Shannon caused by increased precipitation (Δ_IP__Shannon), (c) the change magnitude of Simpson caused by increased precipitation (Δ_IP__Simpson), (d) the change magnitude of Pielou caused by increased precipitation (Δ_IP__Pielou), (e) the change magnitude of Faith’s phylogenetic diversity caused by increased precipitation (Δ_IP__PD), (f) the change magnitude of mean nearest taxon distance caused by increased precipitation (Δ_IP__MNTD), (g) the change magnitude of community aboveground net primary production caused by increased precipitation (Δ_IP__ANPP_community_), (h) the change magnitude of sedge aboveground net primary production caused by increased precipitation (Δ_IP__ANPP_sedge_), (i) the change magnitude of graminoid aboveground net primary production caused by increased precipitation (Δ_IP__ANPP_graminoid_), and (j) the change magnitude of forb aboveground net primary production caused by increased precipitation (Δ_IP__ANPP_forb_); Figure S32: Venn plots of variation partitioning analysis, showing the shared and exclusive effects of increased precipitation duration, Δ_IP__N (i.e., the change magnitude of ammonium nitrogen and/or nitrate nitrogen caused by increased precipitation), Δ_IP__AP (i.e., the change magnitude of soil available phosphorus caused by increased precipitation) and Δ_IP__pH (i.e., the change magnitude of soil pH caused by increased precipitation) on (a) the change magnitude of species richness caused by increased precipitation (Δ_IP__SR), (b) the change magnitude of Shannon caused by increased precipitation (Δ_IP__Shannon), (c) the change magnitude of Simpson caused by increased precipitation (Δ_IP__Simpson), (d) the change magnitude of Pielou caused by increased precipitation (Δ_IP__Pielou), (e) the change magnitude of Faith’s phylogenetic diversity caused by increased precipitation (Δ_IP__PD), (f) the change magnitude of mean nearest taxon distance caused by increased precipitation (Δ_IP__MNTD), (g) the change magnitude of community aboveground net primary production caused by increased precipitation (Δ_IP__ANPP_community_), (h) the change magnitude of sedge aboveground net primary production caused by increased precipitation (Δ_IP__ANPP_sedge_), (i) the change magnitude of graminoid aboveground net primary production caused by increased precipitation (Δ_IP__ANPP_graminoid_), and (j) the change magnitude of forb aboveground net primary production caused by increased precipitation (Δ_IP__ANPP_forb_); Figure S33: Venn plots of variation partitioning analysis, showing the shared and exclusive effects of increased precipitation duration, Δ_IP__T&W (i.e., the change magnitude of air and/or soil temperature, soil moisture, vapor pressure deficit and/or the ratio of growing season precipitation to accumulated ≥5 °C daily air temperature caused by increased precipitation), Δ_IP__N&P (i.e., the change magnitude of ammonium nitrogen, nitrate nitrogen, and/or available phosphorus caused by increased precipitation) and Δ_IP__pH (i.e., the change magnitude of soil pH caused by increased precipitation) on (a) the change magnitude of species richness caused by increased precipitation (Δ_IP__SR), (b) the change magnitude of Shannon caused by increased precipitation (Δ_IP__Shannon), (c) the change magnitude of Simpson caused by increased precipitation (Δ_IP__Simpson), (d) the change magnitude of Pielou caused by increased precipitation (Δ_IP__Pielou), (e) the change magnitude of Faith’s phylogenetic diversity caused by increased precipitation (Δ_IP__PD), (f) the change magnitude of mean nearest taxon distance caused by increased precipitation (Δ_IP__MNTD), (g) the change magnitude of community aboveground net primary production caused by increased precipitation (Δ_IP__ANPP_community_), (h) the change magnitude of sedge aboveground net primary production caused by increased precipitation (Δ_IP__ANPP_sedge_), (i) the change magnitude of graminoid aboveground net primary production caused by increased precipitation (Δ_IP__ANPP_graminoid_), and (j) the change magnitude of forb aboveground net primary production caused by increased precipitation (Δ_IP__ANPP_forb_); Figure S34: Relationships (a) between the response ratio of community aboveground net primary production to increased precipitation (*R*_IP__ANPP_community_) and the response ratio of Shannon to increased precipitation (*R*_IP__Shannon); (b) between the *R*_IP__ANPP_community_ and the response ratio of Simpson to increased precipitation (*R*_IP__Simpson); (c) between the *R*_IP__ANPP_community_ and the response ratio of Pielou to increased precipitation (*R*_IP__Pielou); (d) between the *R*_IP__ANPP_community_ and the species β–diversity of increased precipitation versus non-increased precipitation conditions (βBray_IP_); (e) between the *R*_IP__ANPP_community_ and the phylogenetic β–diversity of increased precipitation versus non-increased precipitation conditions (βMNTD_IP_); (f) between the change magnitude of community aboveground net primary production caused by increased precipitation (Δ_IP__ANPP_community_) and the change magnitude of Shannon caused by increased precipitation (Δ_IP__Shannon); (g) between the Δ_IP__ANPP_community_ and the change magnitude of Simpson caused by increased precipitation (Δ_IP__Simpson); (h) between the Δ_IP__ANPP_community_ and the change magnitude of Pielou caused by increased precipitation (Δ_IP__Pielou); (i) between the Δ_IP__ANPP_community_ and βBray_IP_; and (j) between the Δ_IP__ANPP_community_ and βMNTD_IP_; Figure S35: Venn plots of variation partitioning analysis, showing the shared and exclusive effects of (a) *R*_IP__α-species (i.e., the response ratio of species richness, Shannon, Simpson and/or Pielou to increased precipitation), *R*_IP__α-phylo (i.e., the response ratio of Faith’s phylogenetic diversity and/or mean nearest taxon distance to increased precipitation), βBray (i.e., species β–diversity of increased precipitation versus non-increased precipitation conditions) and βMNTD (i.e., phylogenetic β–diversity of increased precipitation versus non-increased precipitation conditions) on the response ratio of community aboveground net primary production to increased precipitation (*R*_IP__ANPP_community_); and (b) Δ_IP__α-species (i.e., the change magnitude of species richness, Shannon, Simpson and/or Pielou caused by increased precipitation), Δ_IP__α-phylo (i.e., the change magnitude of Faith’s phylogenetic diversity and/or mean nearest taxon distance caused by increased precipitation), βBray (i.e., species β–diversity of increased precipitation versus non-increased precipitation conditions) and βMNTD (i.e., phylogenetic β–diversity of increased precipitation versus non-increased precipitation conditions) on the change magnitude of community aboveground net primary production caused by increased precipitation (Δ_IP__ANPP_community_); Table S1: Repeated-measures analysis of variance was used to estimate the main and interactive effects of experimental warming (W), increased precipitation (IP) and measuring year (Y) on soil temperature (*T*_s_), soil moisture (SM), air temperature (*T*_a_), vapor pressure deficit (VPD), accumulated ≥5 °C daily air temperature (AccT), ratio of growing season precipitation to AccT (GSP/AccT), ammonium nitrogen (NH_4_^+^-N), nitrate nitrogen (NO_3_^−^-N), available phosphorus (AP) and pH.
