# Supplementary material for: Response of Aboveground Net Primary Production, Species and Phylogenetic Diversity to Warming and Increased Precipitation in an Alpine Meadow
Source: Plants (Basel). 2023 Aug 22;12(17):3017. doi: 10.3390/plants12173017 (PMC10490440; doi:10.3390/plants12173017)
Supplement: Supplementary file 1 [file plants-12-03017-s001.zip › plants-2545734-supplementary.pdf]

Table S1 Repeated-measures analysis of variance was used to estimate the main and interactive effects of experimental warming (W), increased precipitation (IP) and measuring year (Y) on soil temperature (T_s), soil moisture (SM), air temperature (T_a), vapor pressure deficit (VPD), accumulated >=5℃ daily air temperature (AccT), ratio of growing season precipitation to AccT (GSP/AccT), ammonium nitrogen (NH_4^+-N), nitrate nitrogen (NO_3^--N), available phosphorus (AP) and pH.

Model	T_s	SM	T_a	VPD	AccT	GSP/AccT	NH_4^+-N	NO_3^--N	AP	pH
Warming(W)	**3.00⁺**	**18.00*** **	1.00	**7.00** **	1.00	**148.00*** **	0.00	0.00	1.00	0.00
Precipitation(IP)	**66.00*** **	**4.00* **	**85.00*** **	**30.00*** **	**82.00*** **	**17.00*** **	**4.00* **	2.00	**6.00** **	1.00
Year(Y)	**13.00*** **	**25.00*** **	**10.00*** **	**34.00*** **	**10.00*** **	**310.00*** **	**41.00*** **	**38.00*** **	**152.00*** **	**117.00*** **
W×IP	**87.00*** **	**16.00*** **	**92.00*** **	**47.00*** **	**88.00*** **	**63.00*** **	1.00	2.00	**3.00⁺**	0.00
W×Y	0.00	0.00	0.00	1.00	0.00	**10.00*** **	**2.00* **	1.00	1.00	0.00
IP×Y	1.00	0.00	1.00	1.00	1.00	**2.00* **	**2.00* **	1.00	**5.00*** **	1.00
W×IP×Y	1.00	0.00	1.00	**2.00* **	1.00	**2.00** **	**2.00** **	1.00	**2.00** **	0.00

⁺, *, ** and *** indicate $p<0.10$, $p<0.05$, $p<0.01$ and $p<0.001$, respectively.

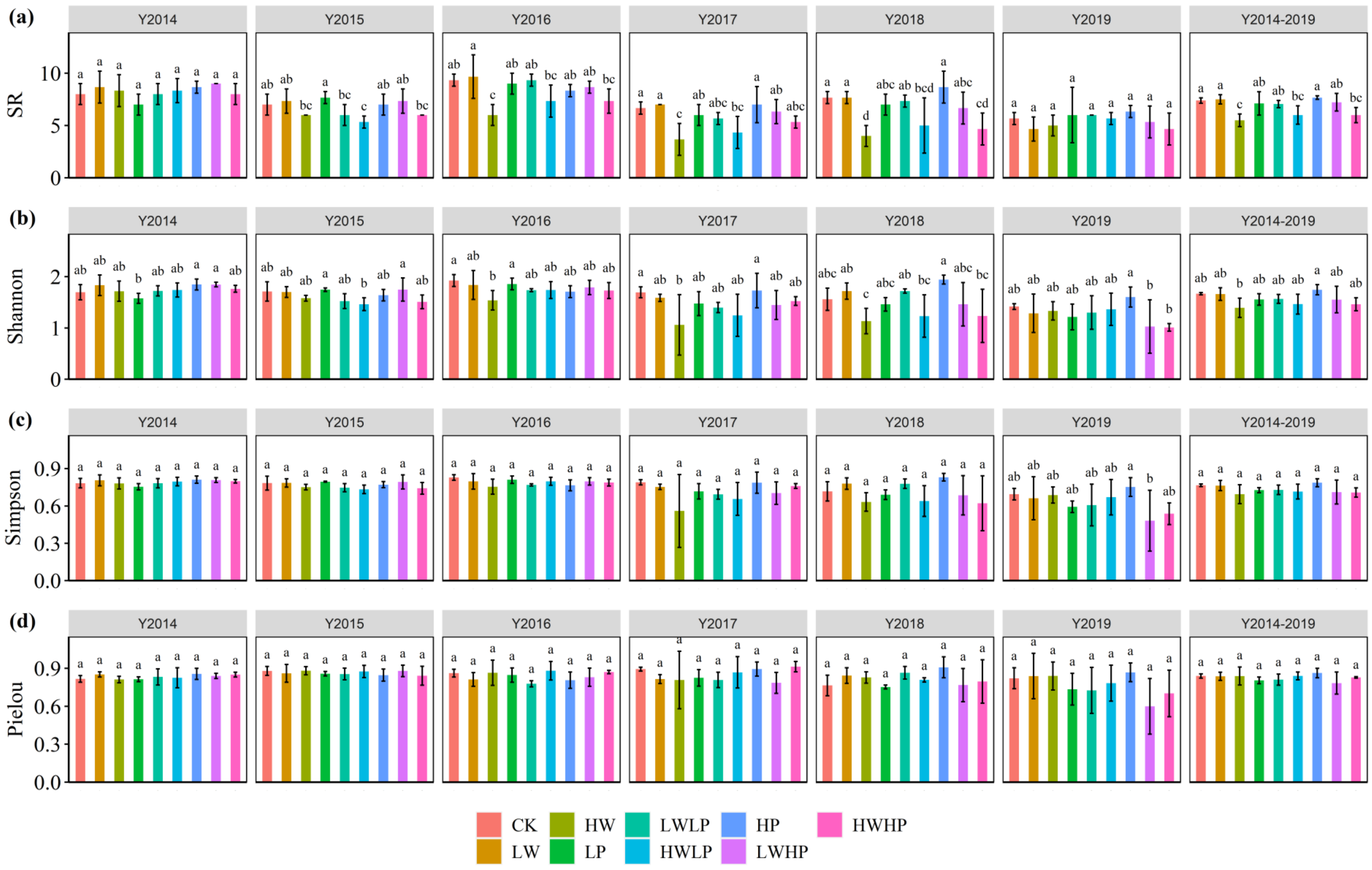

Figure S1 Comparison of species α-diversity (SR: species richness; Shannon, Simpson and Pielou) among the nine experimental treatments in 2014, 2015, 2016, 2017, 2018, 2019 and 2014-2019, respectively. Different letters indicate significant difference at p<0.05.

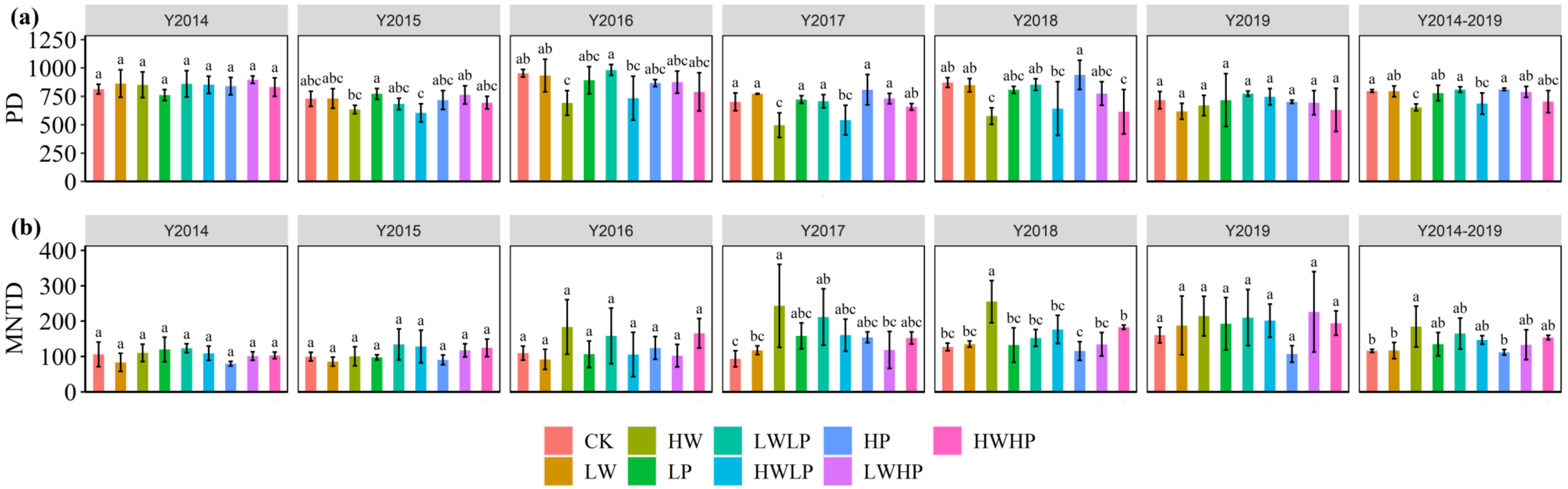

Figure S2 Comparison of phylogenetic α-diversity (PD: Faith's phylogenetic diversity; MNTD: mean nearest taxon distance) among the nine experimental treatments in 2014, 2015, 2016, 2017, 2018, 2019 and 2014-2019, respectively. Different letters indicate significant difference at $p<0.05$.

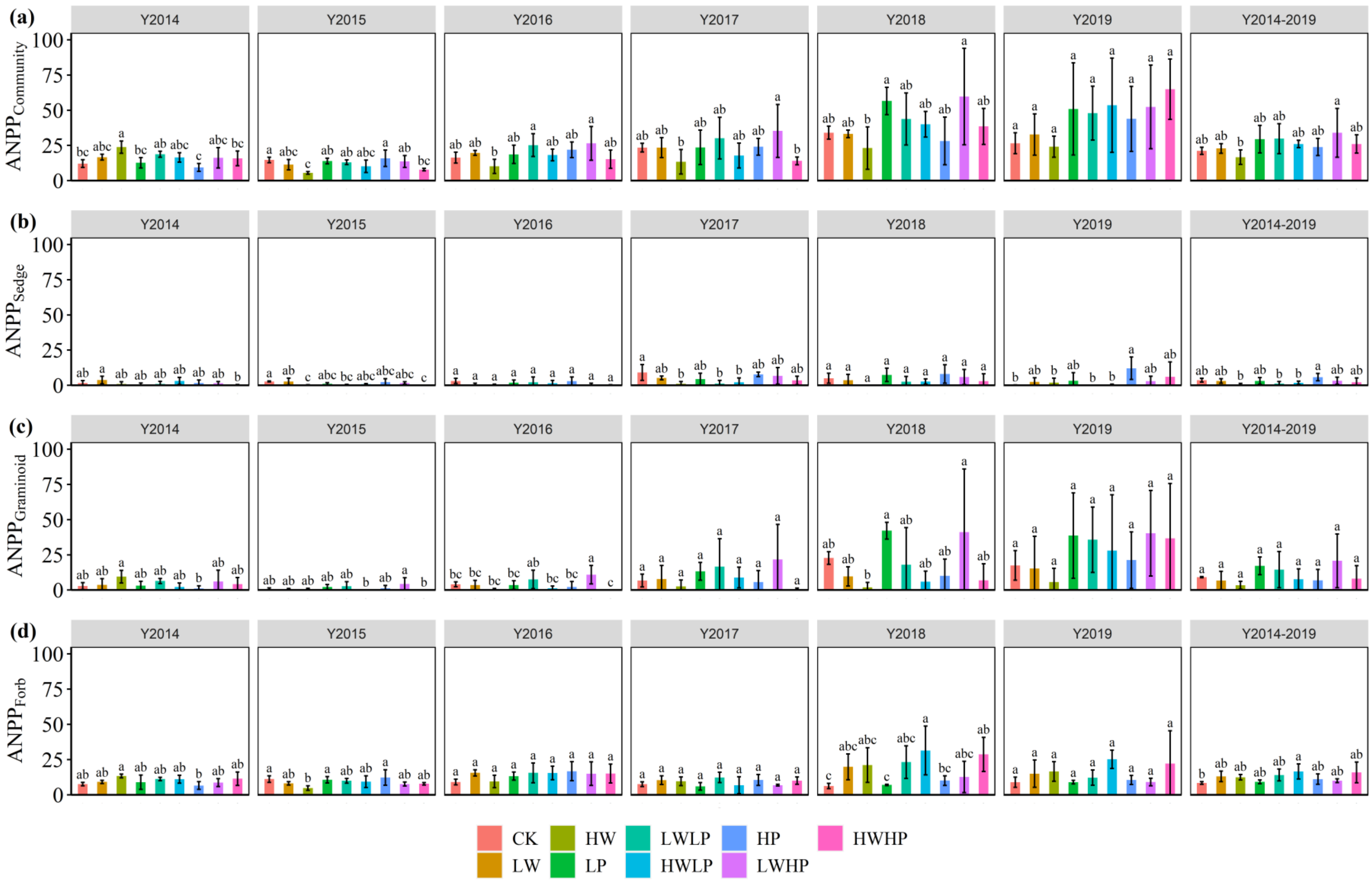

Figure S3 Comparison of aboveground net primary production at community, sedge, graminoid and forb levels ($ANPP_{community}$, $ANPP_{sedge}$, $ANPP_{graminoid}$ and $ANPP_{forb}$) among the nine experimental treatments in 2014, 2015, 2016, 2017, 2018, 2019 and 2014-2019, respectively. Different letters indicate significant difference at $p<0.05$.

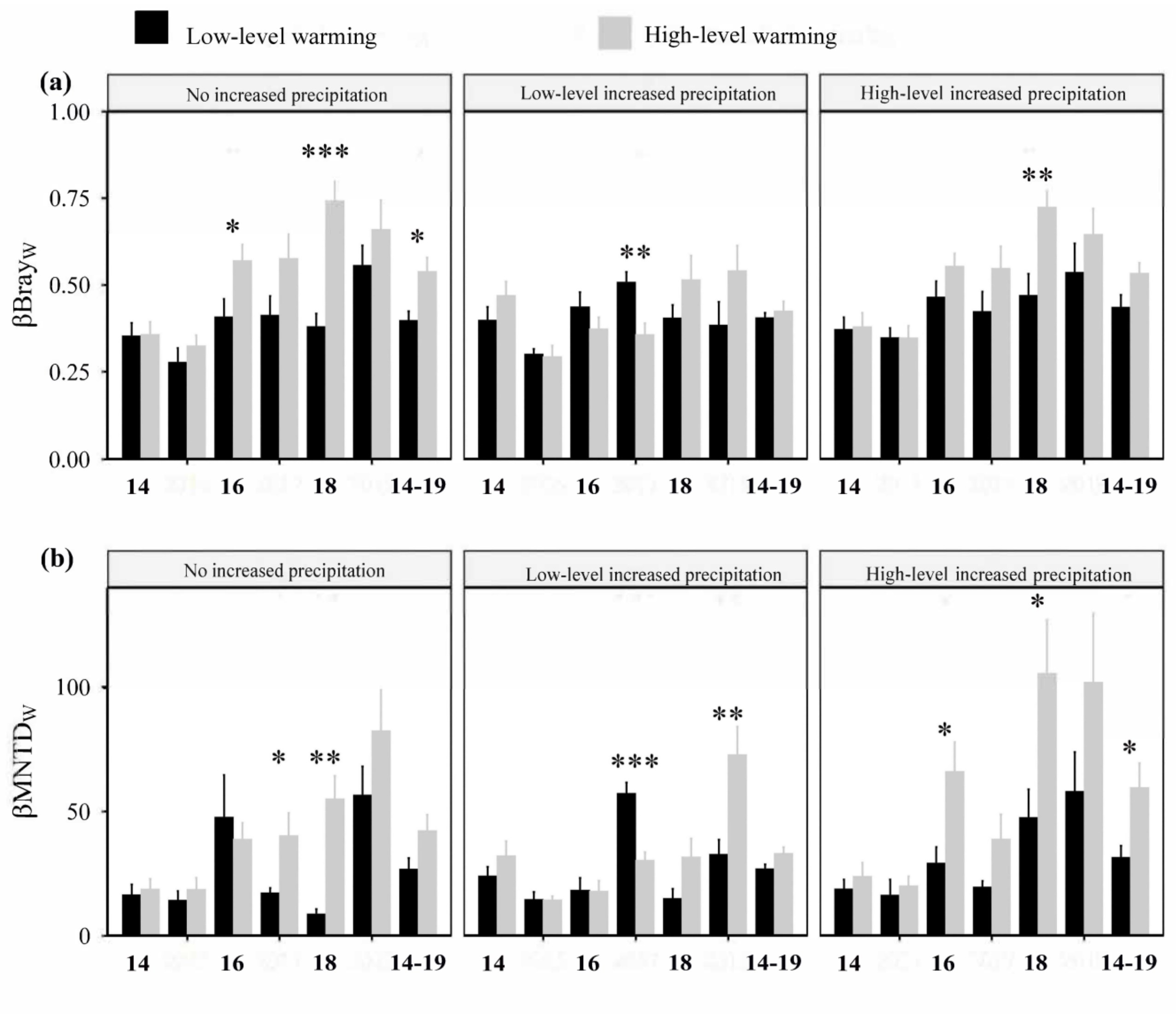

Figure S4 Comparison of the low- and high-level experimental warming-induced dissimilarity of species composition (βBrayW) and phylogenetic composition (βMNTDW) under the no, low- and high-level increased precipitation conditions in 2014, 2015, 2016, 2017, 2018, 2019 and 2014-2019, respectively. *, ** and *** indicate significant difference at p<0.05, p<0.01 and p<0.001, respectively.

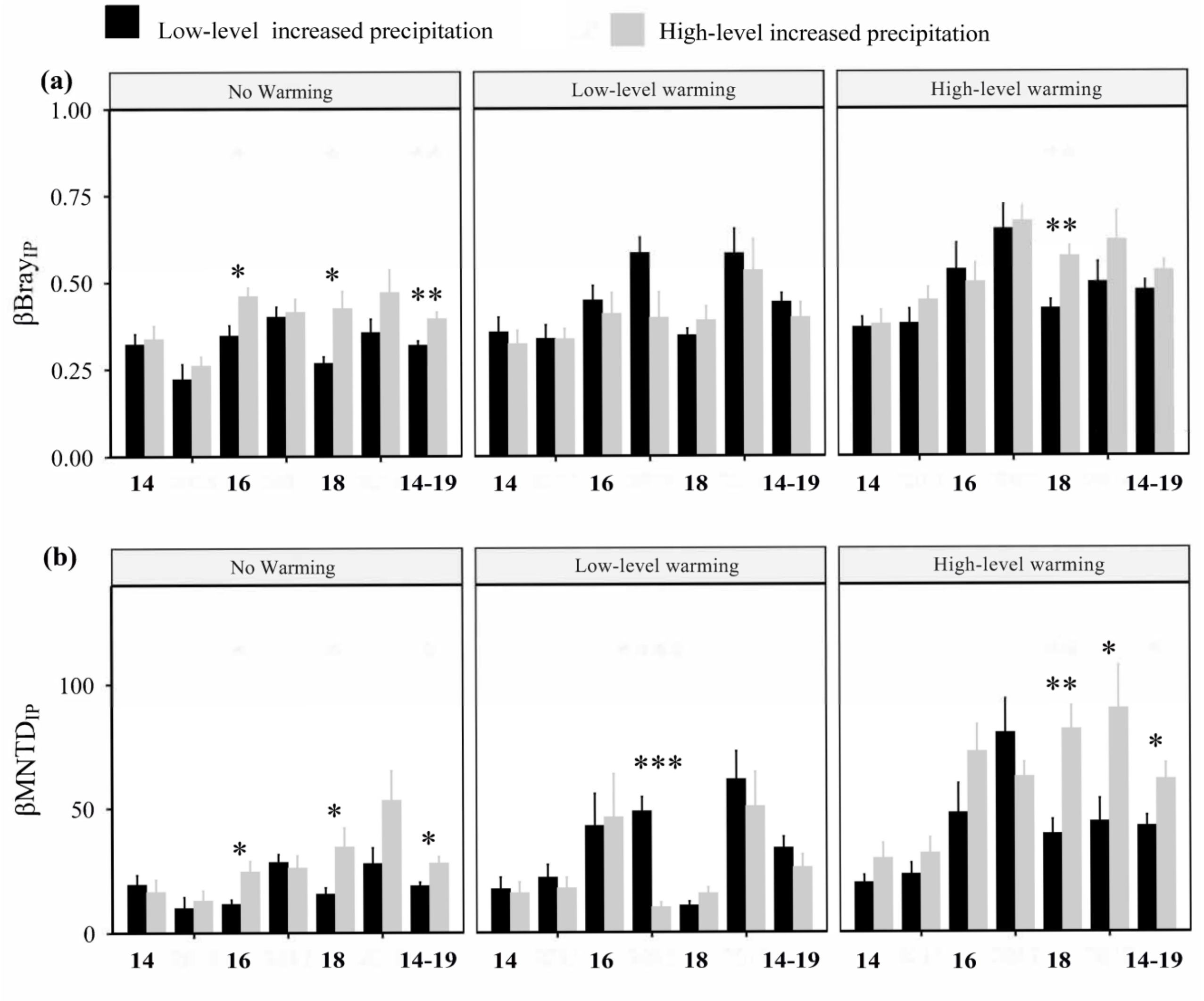

Figure S5 Comparison of the low- and high-level increased precipitation-induced dissimilarity of species composition (βBray$_{IP}$) and phylogenetic composition (βMNTD$_{IP}$) under the no, low- and high-level experimental warming conditions in 2014, 2015, 2016, 2017, 2018, 2019 and 2014-2019, respectively. *, ** and *** indicate significant difference at p<0.05, p<0.01 and p<0.001, respectively.

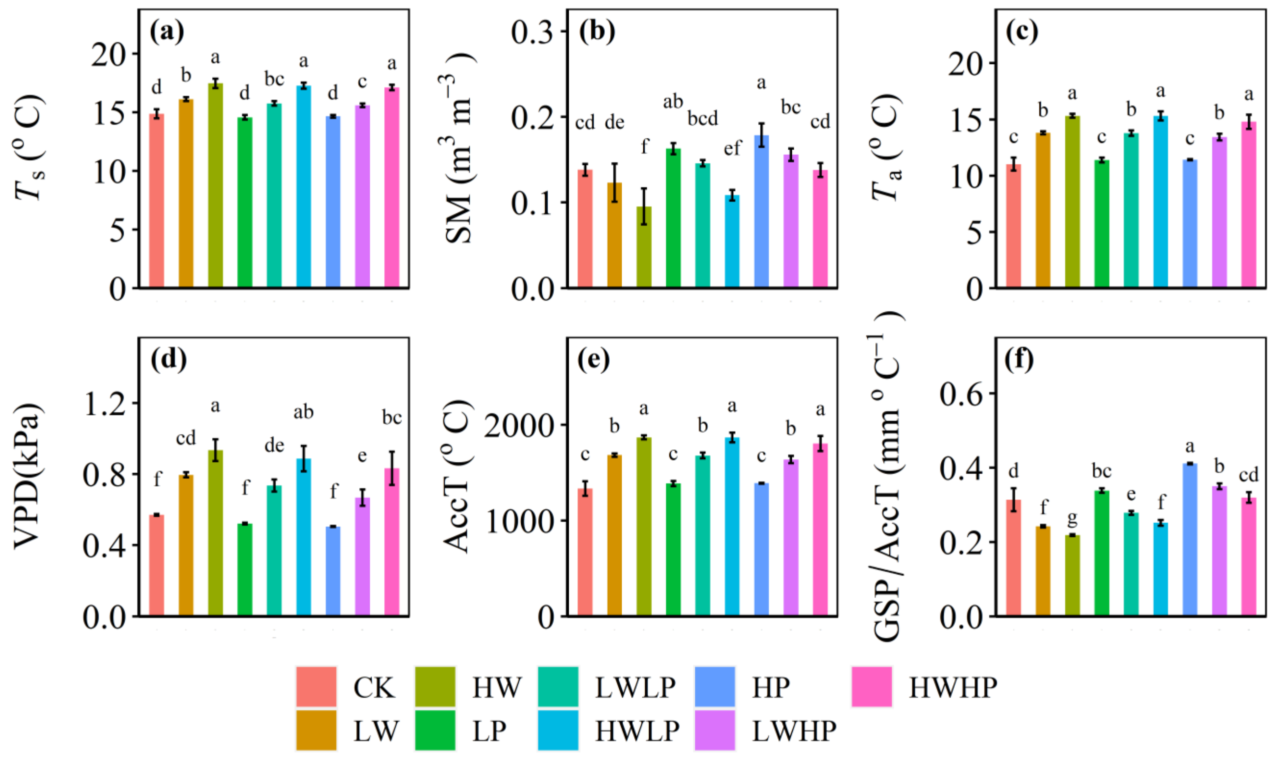

Figure S6 Comparison of average soil temperature (T_s), soil moisture (SM), air temperature (T_a), vapor pressure deficit (VPD), accumulated >=5 °C daily air temperature (AccT), and ratio of growing season precipitation to AccT (GSP/AccT) in 2014-2019 among the nine experimental treatments. Different letters indicate significant difference at p<0.05.

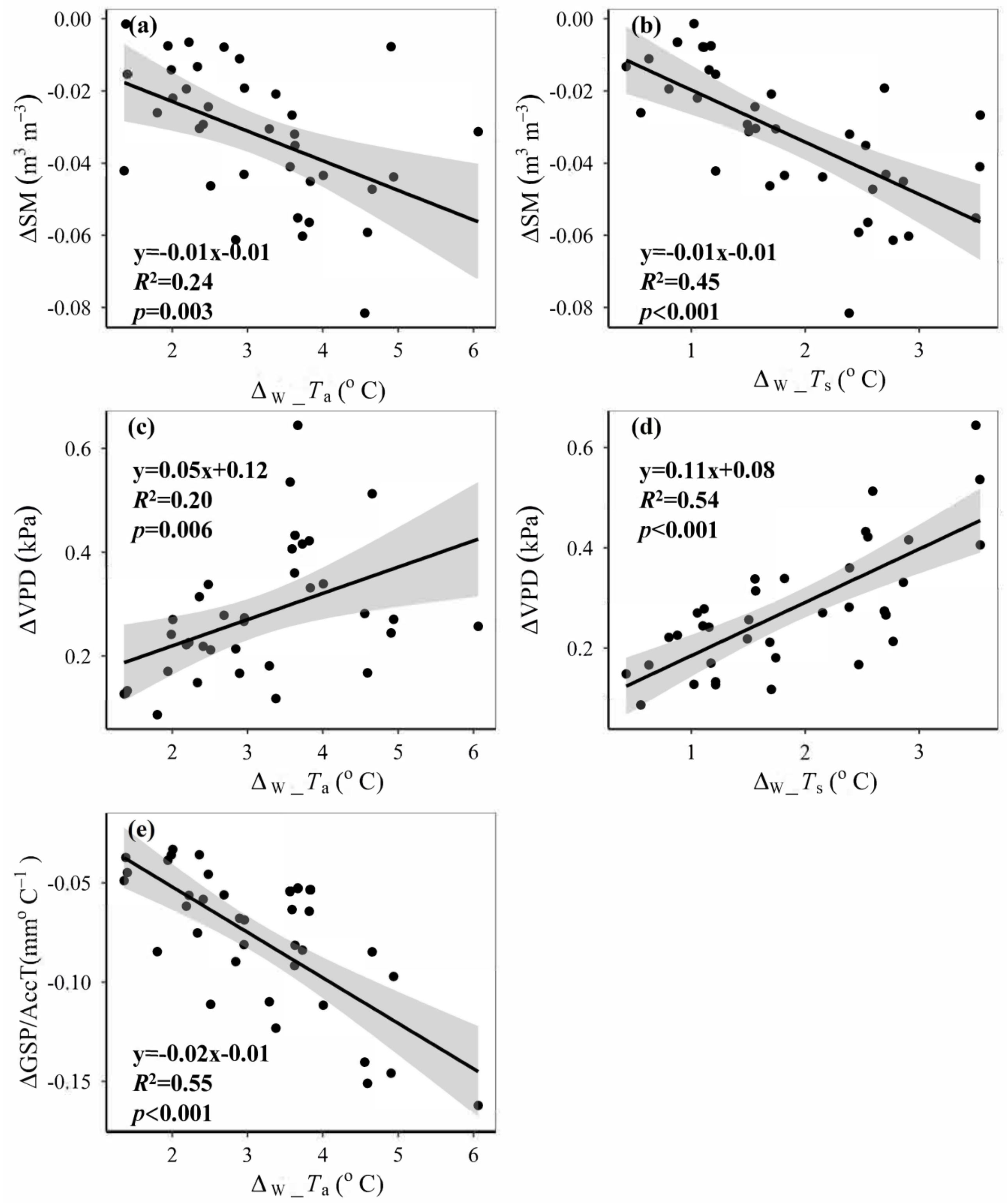

Figure S7 Relationships (a) between the decreased magnitude of soil moisture caused by experimental warming (ΔSM) and increased magnitude of air temperature caused by experimental warming ($\Delta_W\_T_a$); (b) between ΔSM and increased magnitude of soil temperature caused by experimental warming ($\Delta_W\_T_s$); (c) between the increased magnitude of vapor pressure deficit caused by experimental warming (ΔVPD) and $\Delta_W\_T_a$; (d) between ΔVPD and $\Delta_W\_T_s$; and (e) between the decreased magnitude of the ratio of growing season precipitation to accumulated >=5 °C daily air temperature caused by experimental warming (ΔGSP/AccT) and $\Delta_W\_T_a$.

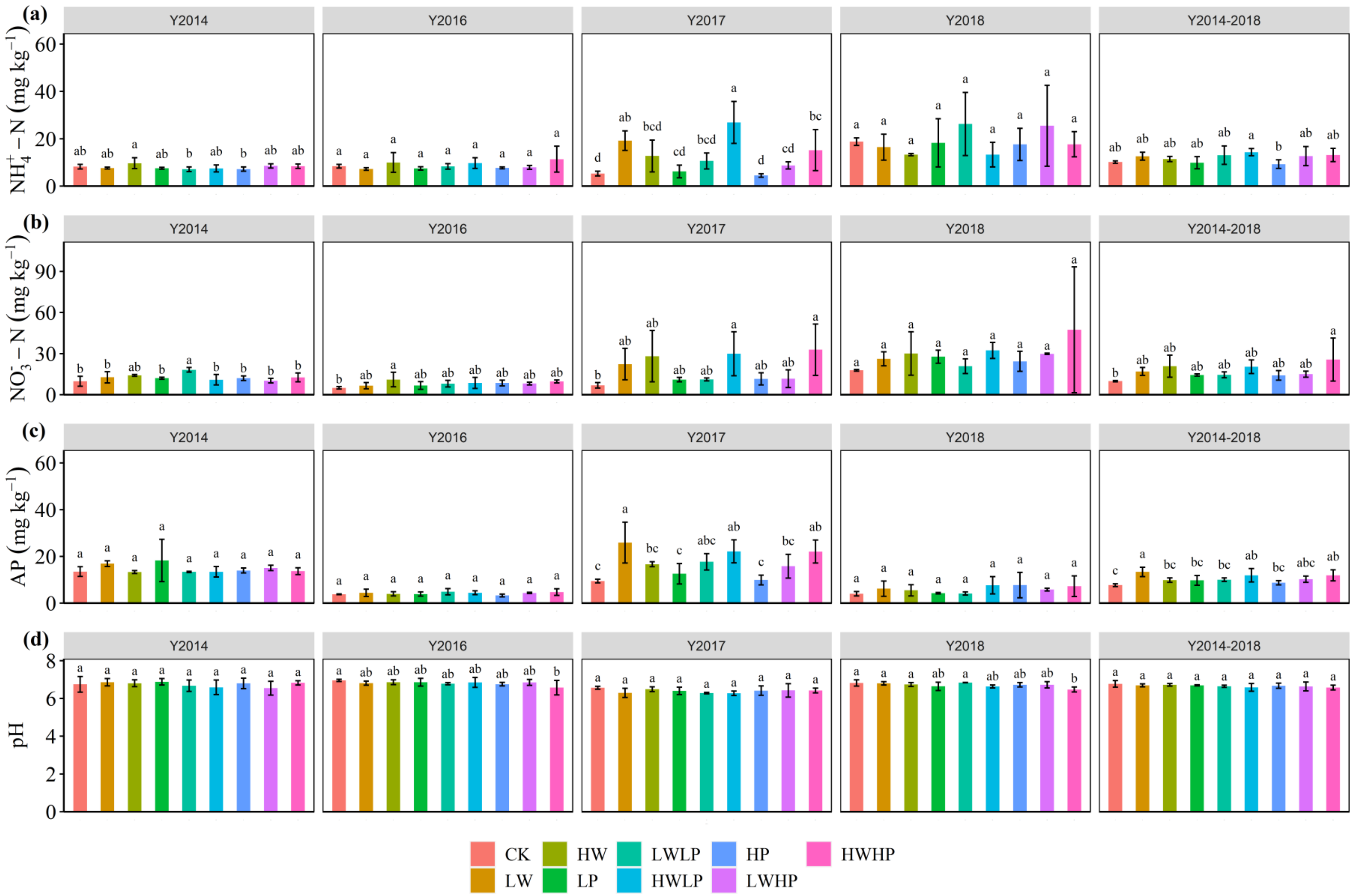

Figure S8 Comparison of ammonium nitrogen (NH_4^+-N), nitrate nitrogen (NO_3^--N), available phosphorus (AP) and pH among the nine experimental treatments in 2014, 2016, 2017, 2018 and 2014-2018, respectively. Different letters indicate significant difference at p<0.05.

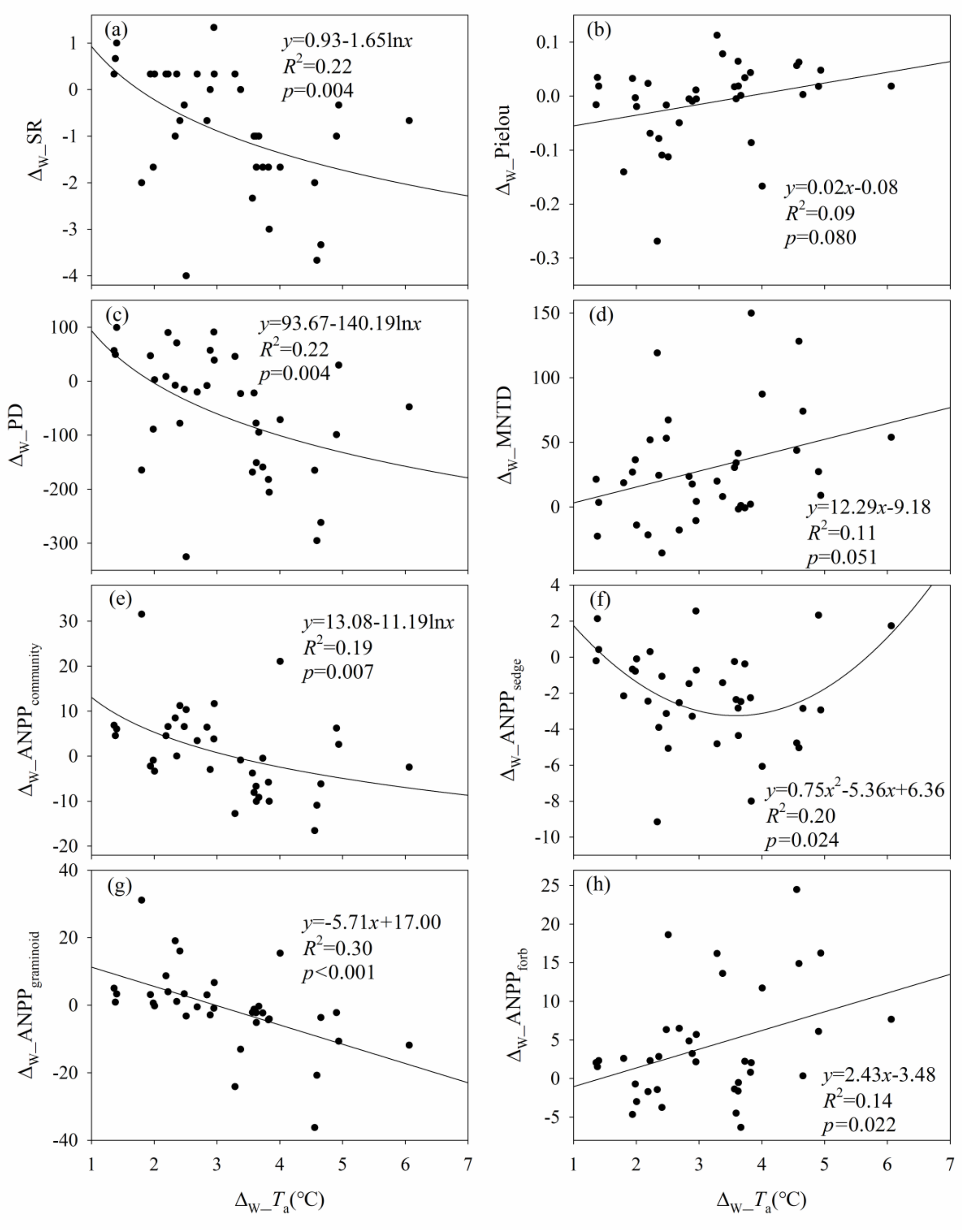

Figure S9 Relationships (a) between the change magnitude of species richness caused by experimental warming (Δ_W_SR) and increased magnitude of air temperature caused by experimental warming (Δ_W_T_a); (b) between the change magnitude of Pielou caused by experimental warming (Δ_W_Pielou) and Δ_W_T_a; (c) between the change magnitude of Faith's phylogenetic diversity caused by experimental warming (Δ_W_PD) and Δ_W_T_a; (d) between the change magnitude of mean nearest taxon distance caused by experimental warming (Δ_W_MNTD) and Δ_W_T_a; (e) between the change magnitude of community aboveground net primary production caused by experimental warming (Δ_W_$ANPP_{community}$) and Δ_W_T_a; (f) between the change magnitude of sedge aboveground net primary production caused by experimental warming (Δ_W_$ANPP_{sedge}$) and Δ_W_T_a; (g) between the change magnitude of graminoid aboveground net primary production caused by experimental warming (Δ_W_$ANPP_{graminoid}$) and Δ_W_T_a; and between the change magnitude of forb aboveground net primary production caused by experimental warming (Δ_W_$ANPP_{forb}$) and Δ_W_T_a.

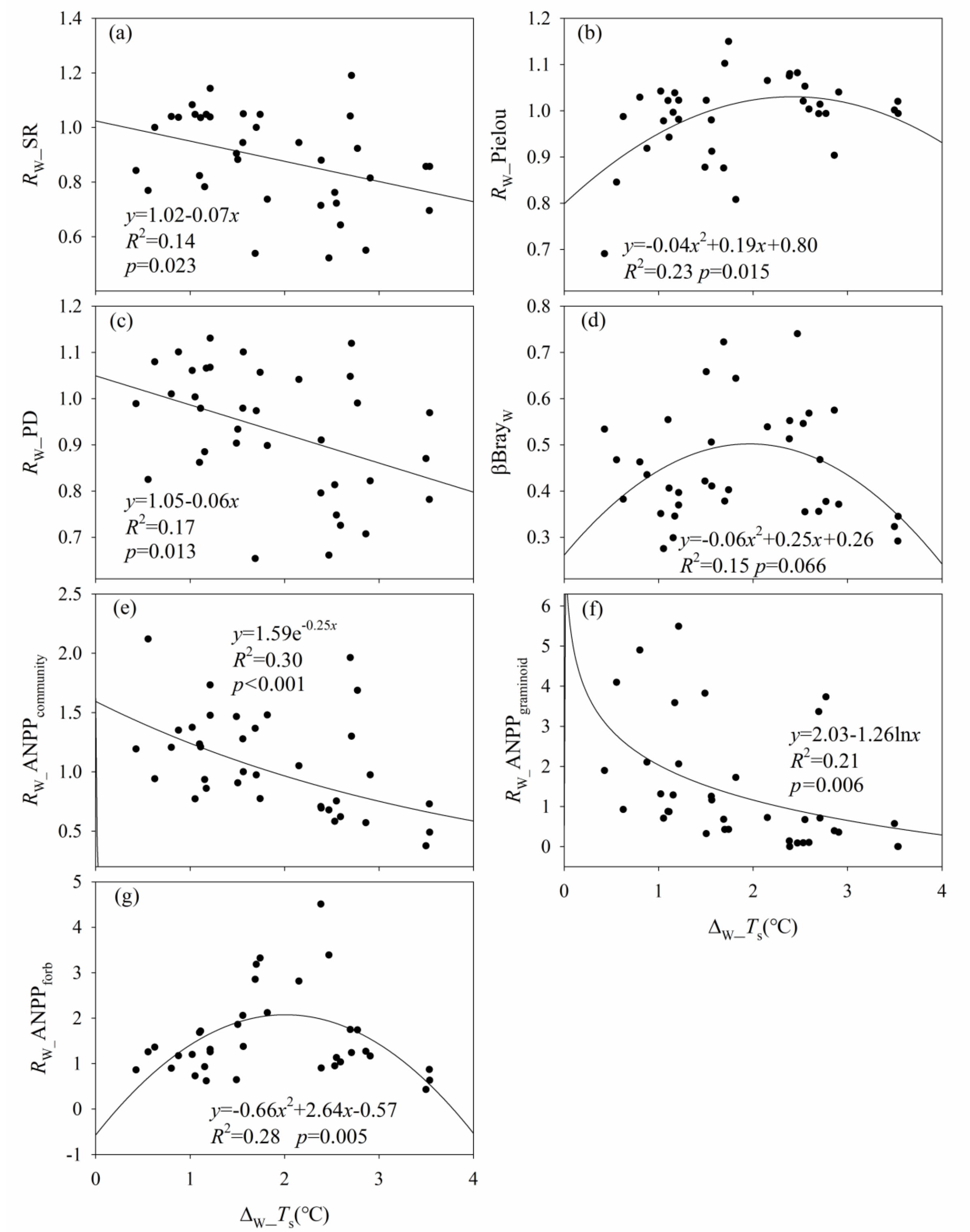

Figure S10 Relationships (a) between the response ratio of species richness to experimental warming (R_W_SR) and increased magnitude of soil temperature caused by experimental warming (Δ_W_T_s); (b) between the response ratio of Pielou to experimental warming (R_W_Pielou) and Δ_W_T_s; (c) between the response ratio of Faith's phylogenetic diversity to experimental warming (R_W_PD) and Δ_W_T_s; (d) between species β-diversity (βBray$_W$) and Δ_W_T_s; (e) between the response ratio of community aboveground net primary production to experimental warming (R_W_ANPP$_{community}$) and Δ_W_T_s; (f) between the response ratio of graminoid aboveground net primary production to experimental warming (R_W_ANPP$_{graminoid}$) and Δ_W_T_s; and (g) between the response ratio of forb aboveground net primary production to experimental warming (R_W_ANPP$_{forb}$) and Δ_W_T_s.

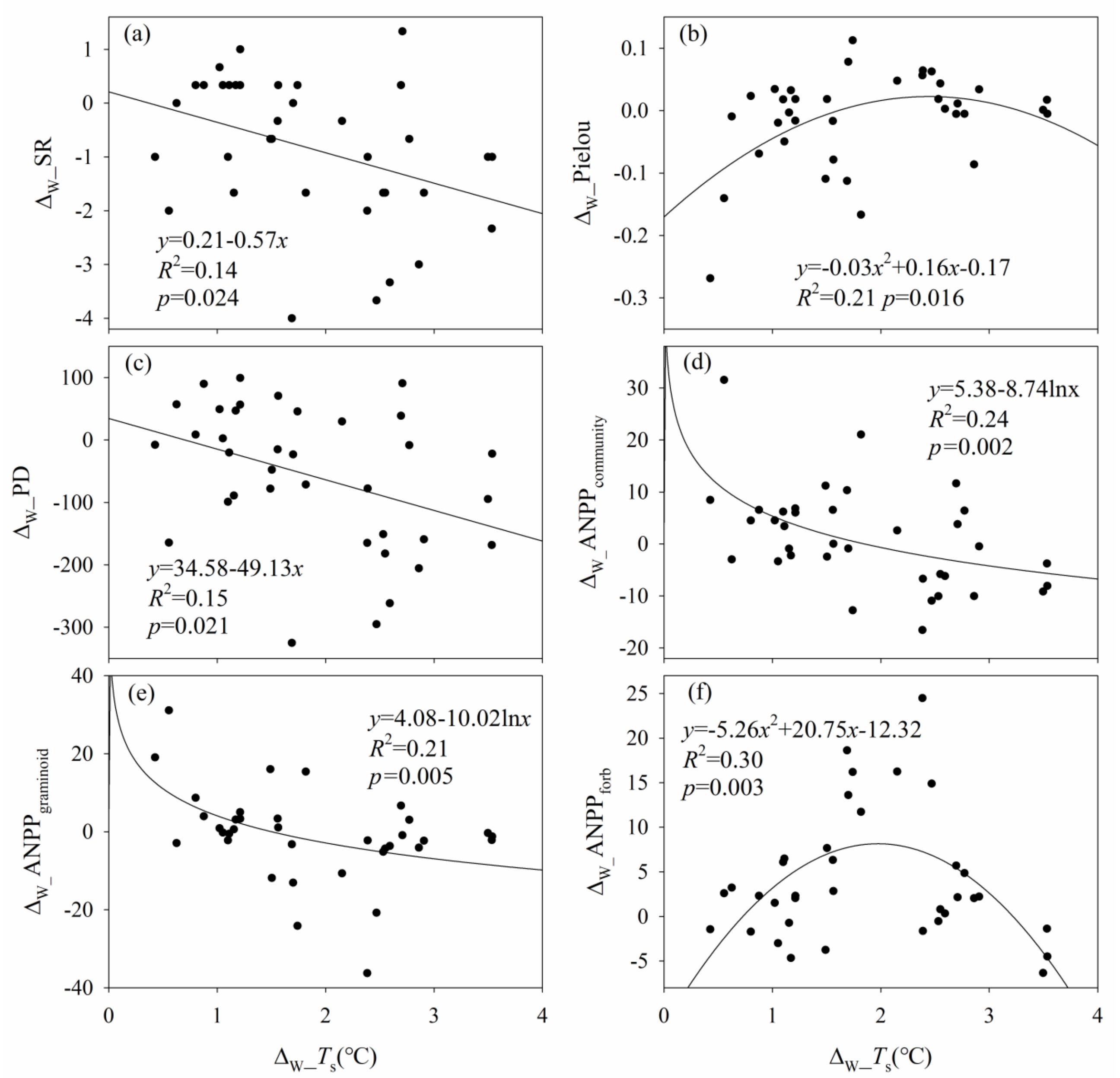

Figure S11 Relationships (a) between the change magnitude of species richness caused by experimental warming (Δ_W_SR) and increased magnitude of soil temperature caused by experimental warming (Δ_W_T_s); (b) between the change magnitude of Pielou caused by experimental warming (Δ_W_Pielou) and Δ_W_T_s; (c) between the change magnitude of Faith's phylogenetic diversity caused by experimental warming (Δ_W_PD) and Δ_W_T_s; (d) between the change magnitude of community aboveground net primary production caused by experimental warming (Δ_W_$ANPP_{community}$) and Δ_W_T_s; (e) between the change magnitude of graminoid aboveground net primary production caused by experimental warming (Δ_W_$ANPP_{graminoid}$) and Δ_W_T_s; and (f) between the change magnitude of forb aboveground net primary production caused by experimental warming (Δ_W_$ANPP_{forb}$) and Δ_W_T_s.

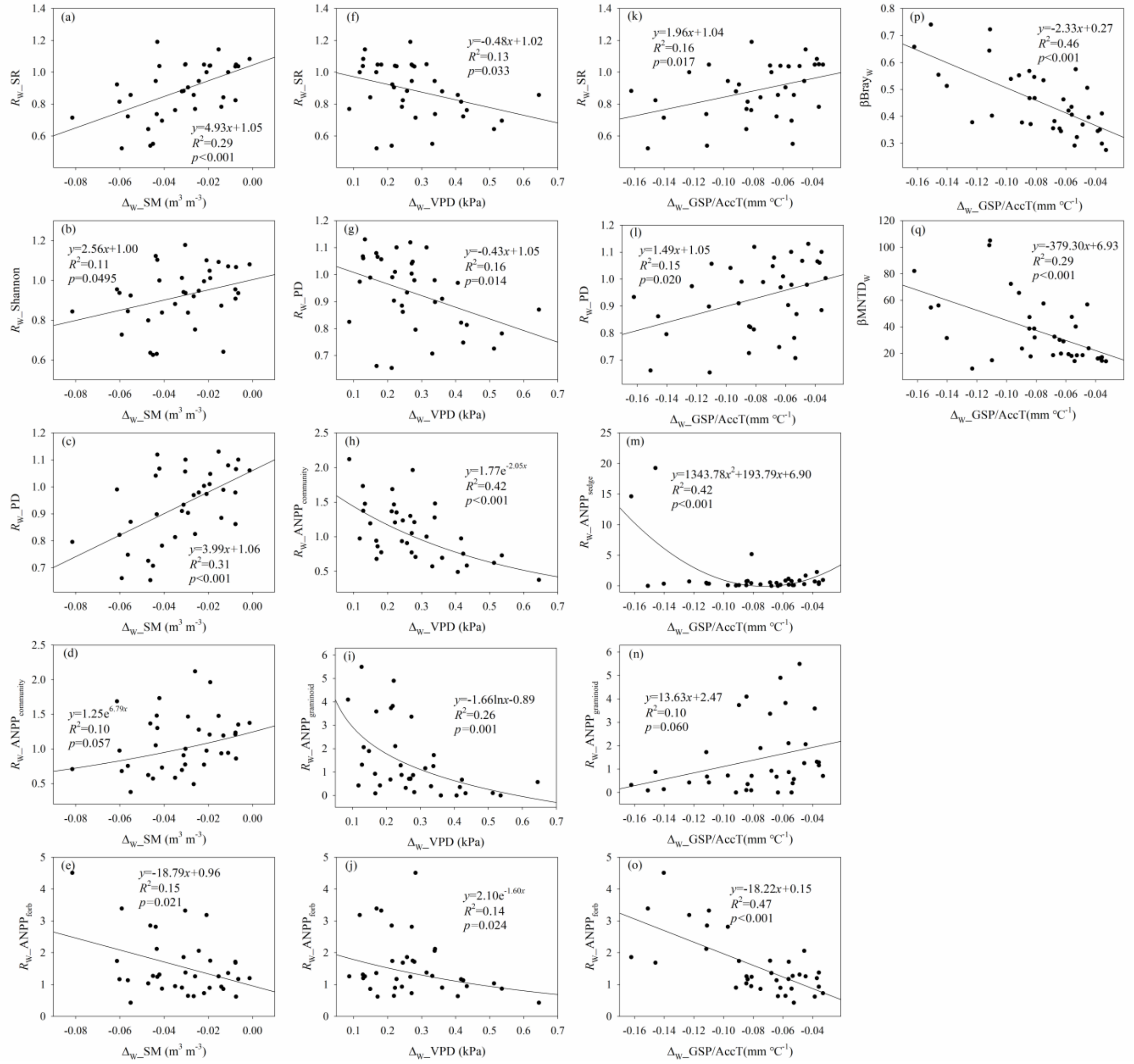

Figure S12 Relationships (a) between the response ratio of species richness to experimental warming (R_W_SR) and decreased magnitude of soil moisture caused by experimental warming (Δ_W_SM); (b) between the response ratio of Shannon to experimental warming (R_W_Shannon) and Δ_W_SM; (c) between the response ratio of Faith's phylogenetic diversity to experimental warming (R_W_PD) and Δ_W_SM; (d) between the response ratio of community aboveground net primary production to experimental warming (R_W_ANPP$_{community}$) and Δ_W_SM; (e) between the response ratio of forb aboveground net primary production to experimental warming (R_W_ANPP$_{forb}$) and Δ_W_SM; (f) between the R_W_SR and increased magnitude of vapor pressure deficit caused by experimental warming (Δ_W_VPD); (g) between the R_W_PD and Δ_W_VPD; (h) between the R_W_ANPP$_{community}$ and Δ_W_VPD; (i) between the response ratio of graminoid aboveground net primary production to experimental warming (R_W_ANPP$_{graminoid}$) and Δ_W_VPD; (j) between the R_W_ANPP$_{forb}$ and Δ_W_VPD; (k) between the R_W_SR and decreased magnitude of accumulated >=5 ^{o}C daily air temperature caused by experimental warming (Δ_W_GSP/AccT); (l) between the R_W_PD and Δ_W_GSP/AccT; (m) between the response ratio of sedge aboveground net primary production to experimental warming (R_W_ANPP$_{sedge}$) and Δ_W_GSP/AccT; (n) between the R_W_ANPP$_{graminoid}$ and Δ_W_GSP/AccT; (o) between the R_W_ANPP$_{forb}$ and Δ_W_GSP/AccT; (p) between the species β-diversity of warming versus no-warming conditions (βBray$_W$) and Δ_W_GSP/AccT; and (q) between the phylogenetic β-diversity of warming versus no-warming conditions (βMNTD$_W$) and Δ_W_GSP/AccT.

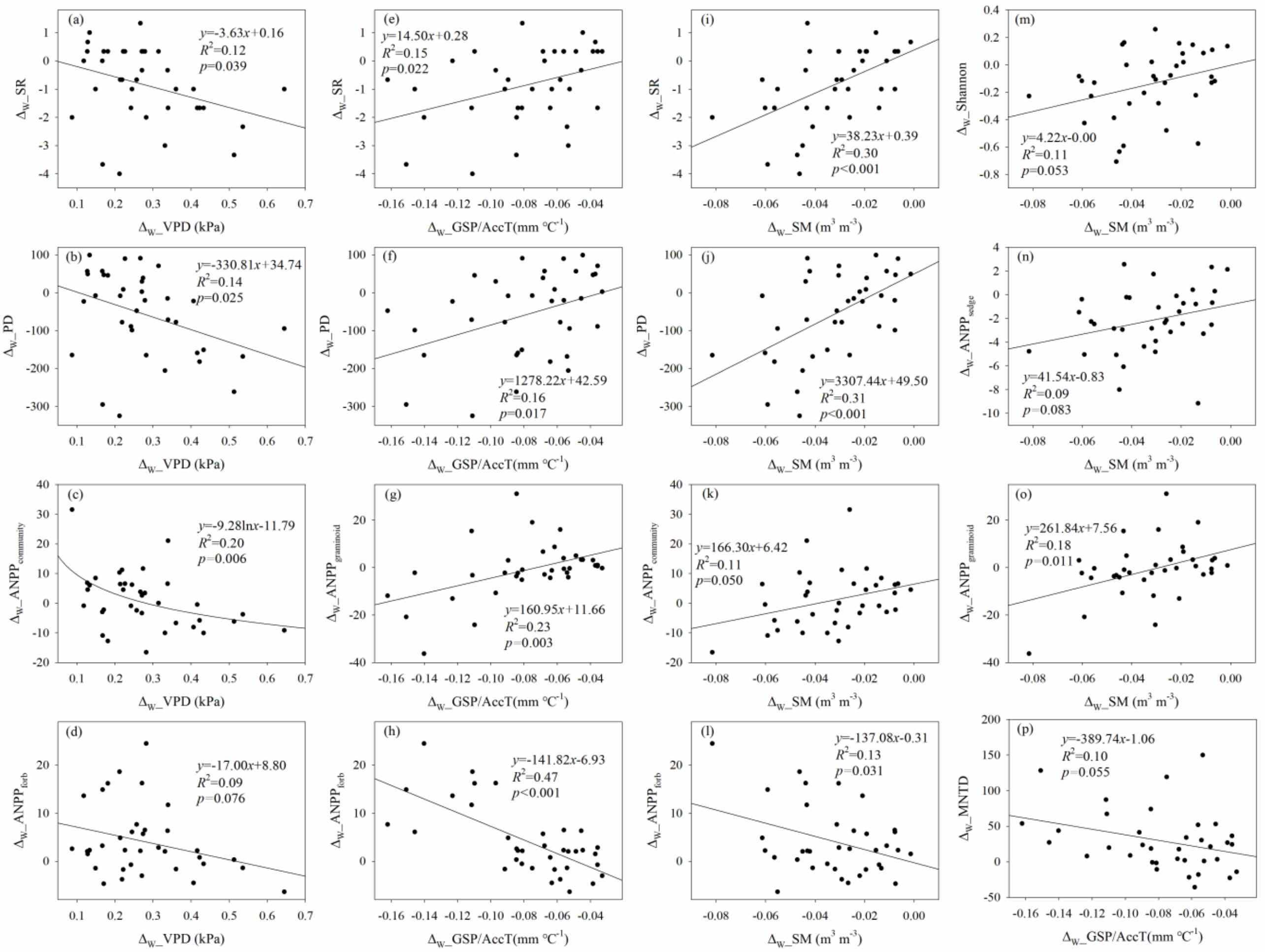

Figure S13 Relationships (a) between change magnitude of species richness caused by experimental warming (Δw_SR) and increased magnitude of vapor pressure deficit caused by experimental warming (Δw_VPD); (b) between the change magnitude of Faith's phylogenetic diversity caused by experimental warming (Δw_PD) and Δw_VPD; (c) between the change magnitude of community aboveground net primary production caused by experimental warming (Δw_$ANPP_{community}$) and Δw_VPD; (d) between the change magnitude of forb aboveground net primary production caused by experimental warming (Δw_$ANPP_{forb}$) and Δw_VPD; (e) between the Δw_SR and decreased magnitude of accumulated >=5 ^{o}C daily air temperature caused by experimental warming (Δw_GSP/AccT); (f) between Δw_PD and Δw_GSP/AccT; (g) between the change magnitude of graminoid aboveground net primary production caused by experimental warming (Δw_$ANPP_{graminoid}$) and ΔGSP/AccT; (h) between the Δw_$ANPP_{forb}$ and Δw_GSP/AccT; (i) between the Δw_SR and the decreased magnitude of soil moisture caused by experimental warming (ΔSM); (j) between the Δw_PD and Δw_SM; (k) between the Δw_$ANPP_{community}$ and Δw_SM; (l) between the Δw_$ANPP_{forb}$ and Δw_SM; (m) between the change magnitude of Shannon caused by experimental warming (Δw_Shannon) and Δw_SM; (n) between the change magnitude of sedge aboveground net primary production caused by experimental warming (Δw_$ANPP_{sedge}$) and Δw_SM; (o) between the Δw_$ANPP_{graminoid}$ and Δw_SM; and (p) between the change magnitude of mean nearest taxon distance caused by experimental warming (Δw_MNTD) and ΔGSP/AccT.

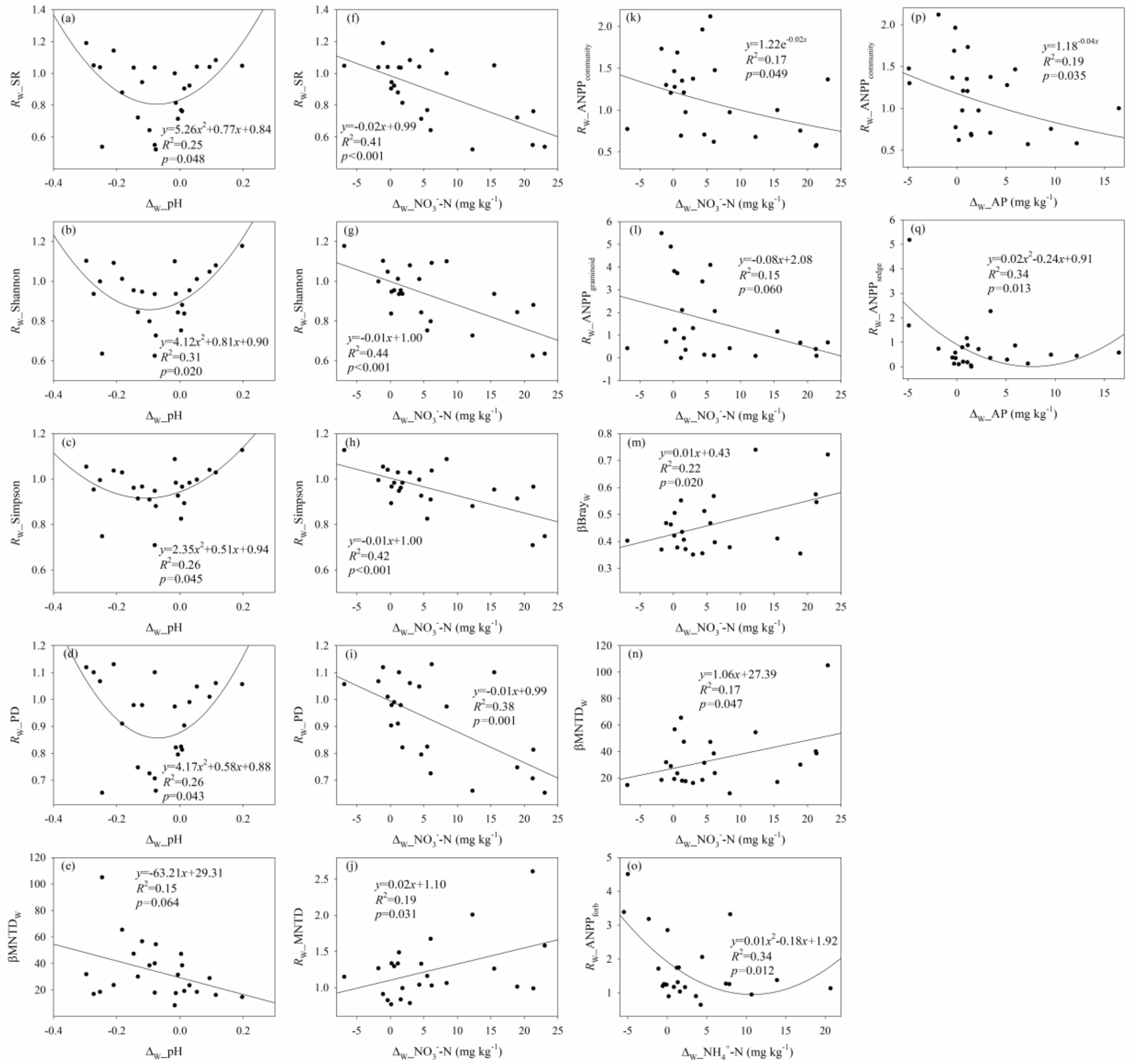

Figure S14 Relationships (a) between the response ratio of species richness to experimental warming (*R*w_SR) and change magnitude of soil pH caused by experimental warming (Δw_pH); (b) between the response ratio of Shannon to experimental warming (*R*w_Shannon) and Δw_pH; (c) between the response ratio of Simpson to experimental warming (*R*w_Simpson) and Δw_pH; (d) between the response ratio of Faith's phylogenetic diversity to experimental warming (*R*w_PD) and Δw_pH; (e) between the phylogenetic β-diversity of warming versus no-warming conditions (βMNTD$_w$) and Δw_pH; (f) between the *R*w_SR and the change magnitude of nitrate nitrogen (Δw_NO_3^--N) caused by experimental warming; (g) between the *R*w_Shannon and Δw_NO_3^--N; (h) between the *R*w_Simpson and Δw_NO_3^--N; (i) between the *R*w_PD and Δw_NO_3^--N; (j) between the response ratio of mean nearest taxon distance to experimental warming (*R*w_MNTD) and Δw_NO_3^--N; (k) between the response ratio of community aboveground net primary production to experimental warming (*R*w_ANPP$_{community}$) and Δw_NO_3^--N; (l) between the response ratio of graminoid aboveground net primary production to experimental warming (*R*w_ANPP$_{graminoid}$) and Δw_NO_3^--N; (m) between species β-diversity of warming versus no-warming conditions (βBray$_w$) and Δw_NO_3^--N; (n) between the βMNTD$_w$ and Δw_NO_3^--N; (o) between the response ratio of forb aboveground net primary production to experimental warming (*R*w_ANPP$_{forb}$) and the change magnitude of ammonium nitrogen (Δw_NH_4^+-N) caused by experimental warming; (p) between the *R*w_ANPP$_{community}$ and the change magnitude of available phosphorus (Δw_AP) caused by experimental warming; and (q) between the response ratio of sedge aboveground net primary production to experimental warming (*R*w_ANPP$_{sedge}$) and Δw_AP.

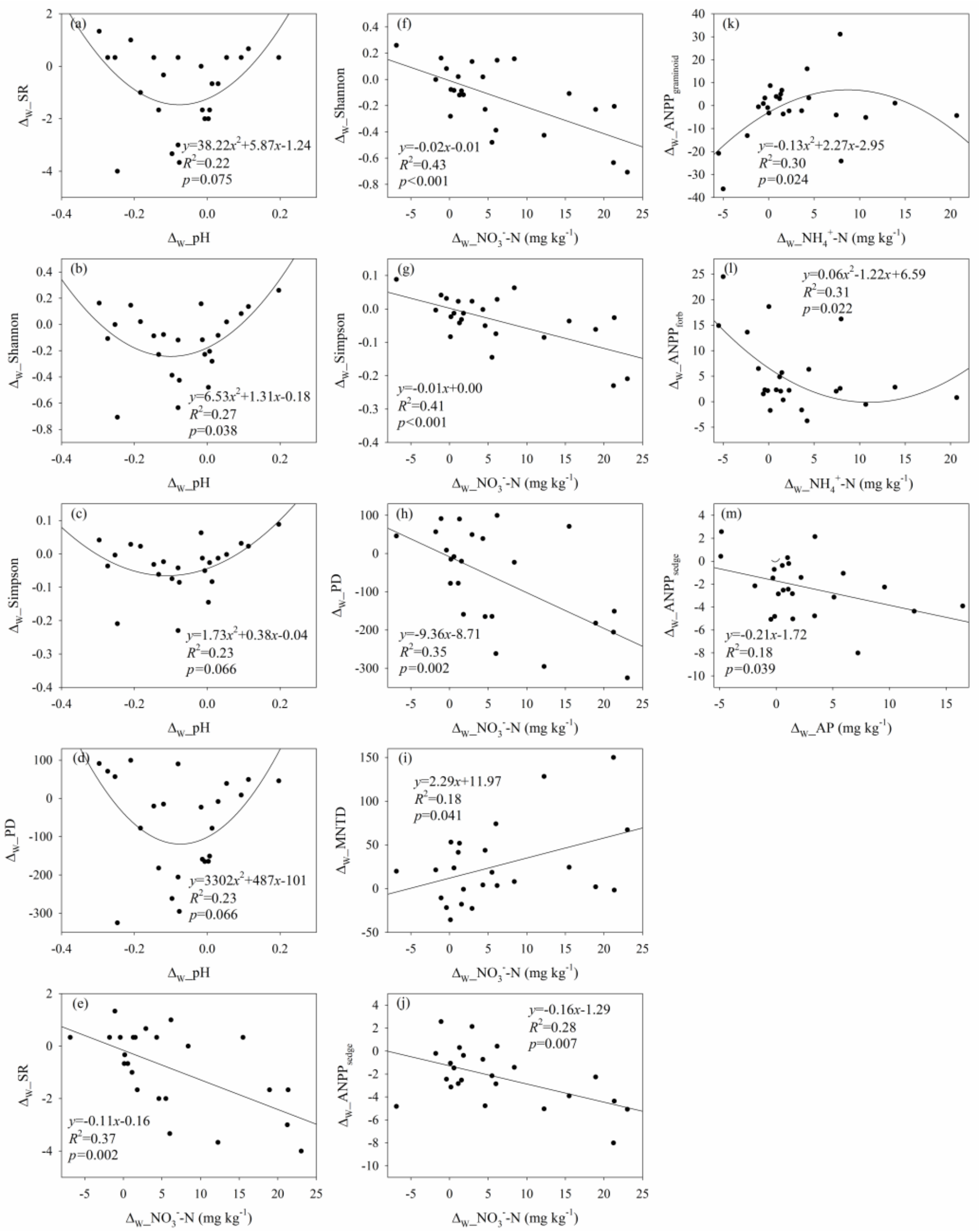

Figure S15 Relationships (a) between the change magnitude of species richness caused by experimental warming (Δw_SR) and change magnitude of soil pH caused by experimental warming (Δw_pH); (b) between the change magnitude of Shannon caused by experimental warming (Δw_Shannon) and Δw_pH; (c) between the change magnitude of Simpson caused by experimental warming (Δw_Simpson) and Δw_pH; (d) between the change magnitude of Faith's phylogenetic diversity caused by experimental warming (Δw_PD) and Δw_pH; (e) between the Δw_SR and the change magnitude of nitrate nitrogen (Δw_NO_3^--N) caused by experimental warming; (f) between the Δw_Shannon and Δw_NO_3^--N; (g) between the Δw_Simpson and Δw_NO_3^--N; (h) between the Δw_PD and Δw_NO_3^--N; (i) between the change magnitude of mean nearest taxon distance to experimental warming (Δw_MNTD) and Δw_NO_3^--N; (j) between the change magnitude of sedge aboveground net primary production caused by experimental warming (Δw_$ANPP_{sedge}$) and Δw_NO_3^--N; (k) between the change magnitude of graminoid aboveground net primary production caused by experimental warming (*R*w_$ANPP_{graminoid}$) and the change magnitude of ammonium nitrogen (Δw_NH_4^+-N) caused by experimental warming; (l) between the change magnitude of forb aboveground net primary production caused by experimental warming (*R*w_$ANPP_{forb}$) and Δw_NH_4^+-N; and (m) between the *R*w_$ANPP_{sedge}$ and the change magnitude of available phosphorus (Δw_AP) caused by experimental warming.

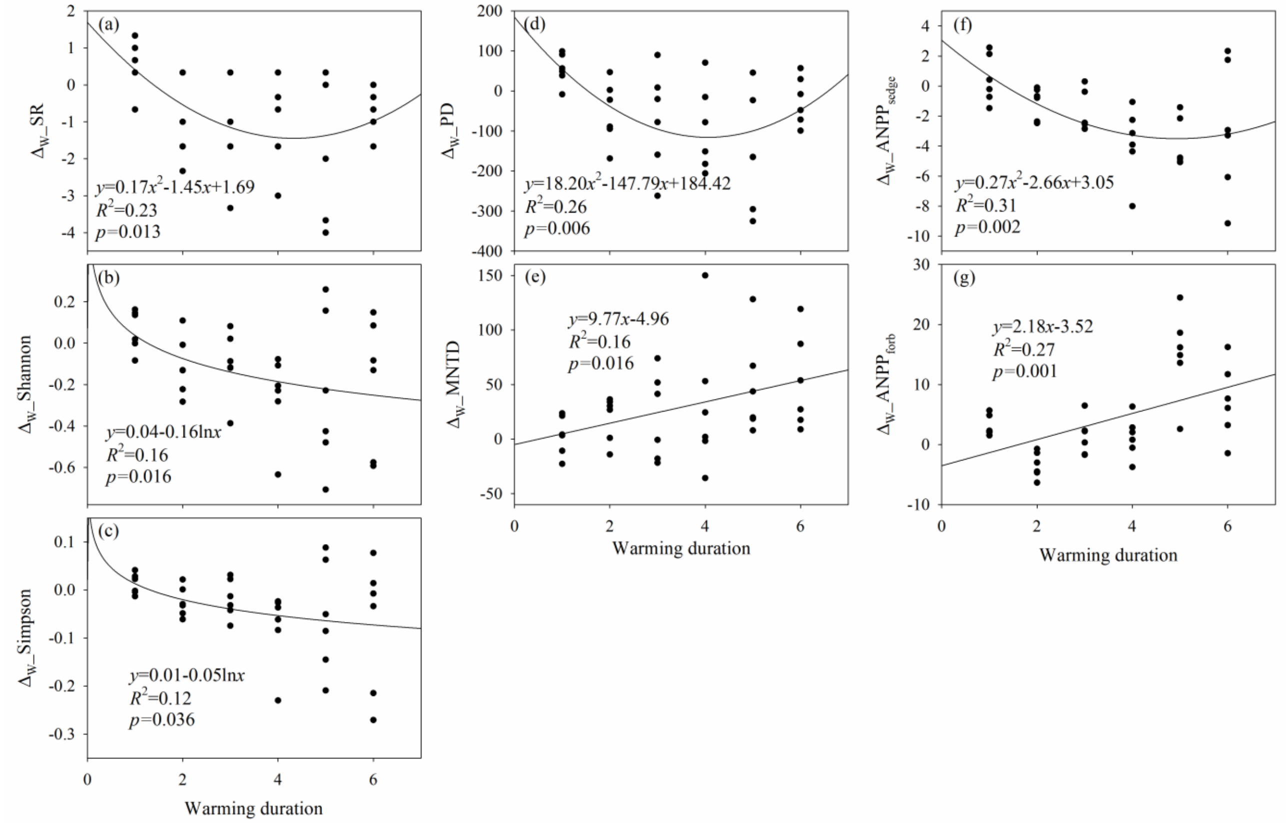

Figure S16 Relationships (a) between the change magnitude of species richness caused by experimental warming (Δw_SR) and warming duration; (b) between the change magnitude of Shannon caused by experimental warming (Δw_Shannon) and warming duration; (c) between the change magnitude of Simpson caused by experimental warming (Δw_Simpson) and warming duration; (d) between the change magnitude of Faith's phylogenetic diversity caused by experimental warming (Δw_PD) and warming duration; (e) between the change magnitude of mean nearest taxon distance to experimental warming (Δw_MNTD) and warming duration; (f) between the change magnitude of sedge aboveground net primary production caused by experimental warming (Δw_ANPP$_{sedge}$) and warming duration; and (g) between the change magnitude of forb aboveground net primary production caused by experimental warming (Δw_ANPP$_{forb}$) and warming duration.

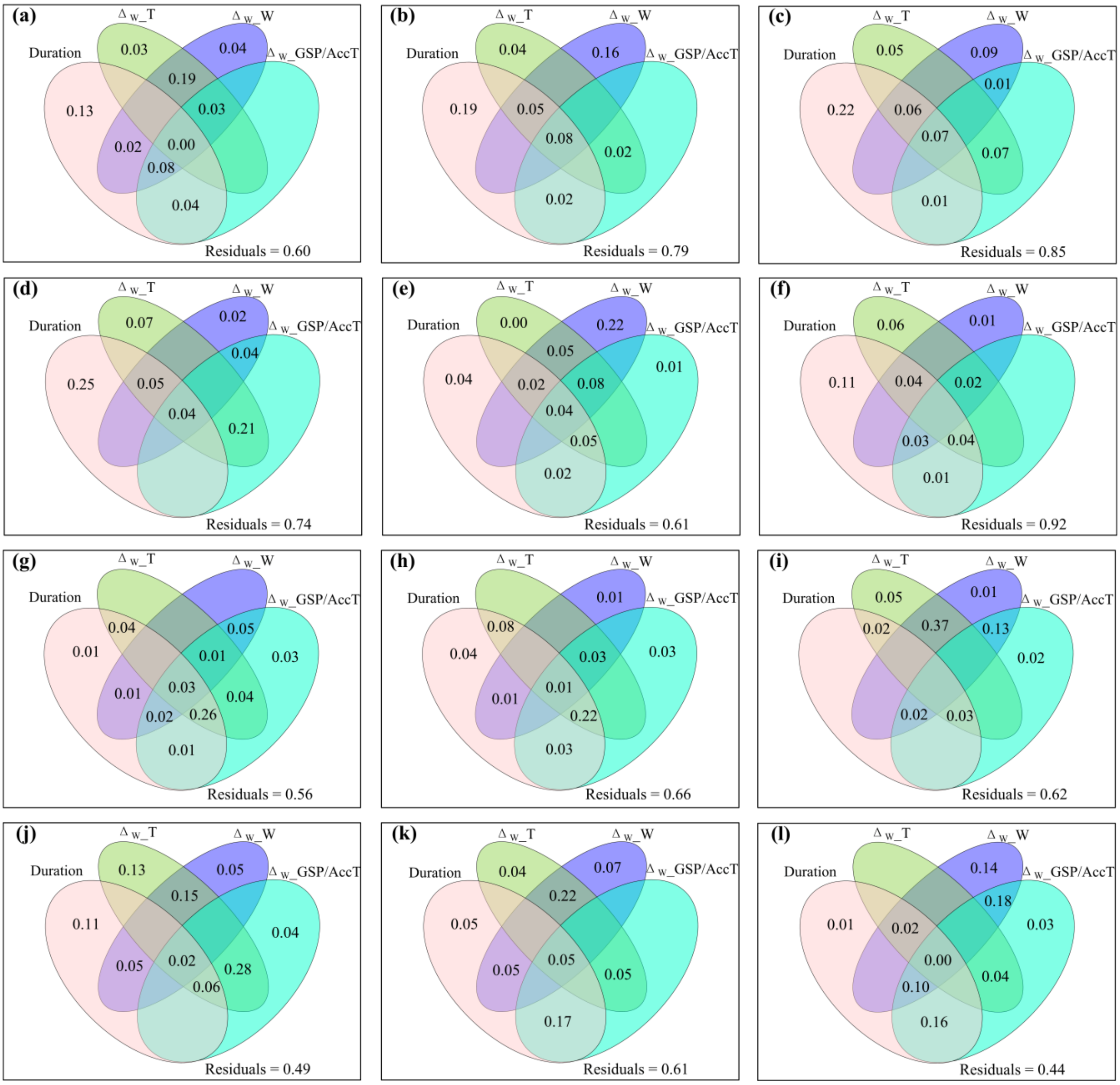

Figure S17 Venn plots of variation partitioning analysis, showing the shared and exclusive effects of warming duration, Δ_W_T (i.e. the change magnitude of air and/or soil temperature caused by experimental warming), Δ_W_W (i.e. the change magnitude of soil moisture and/or vapor pressure deficit caused by experimental warming) and Δ_W_GSP/AccT (i.e. the change magnitude of the ratio of growing season precipitation to accumulated >=5 ^{o}C daily air temperature caused by experimental warming) on (a) the response ratio of species richness to experimental warming (R_W_SR), (b) the response ratio of Shannon to experimental warming (R_W_Shannon), (c) the response ratio of Simpson to experimental warming (R_W_Simpson), (d) the response ratio of Pielou to experimental warming (R_W_Pielou), (e) the response ratio of Faith's phylogenetic diversity to experimental warming (R_W_PD), (f) the response ratio of mean nearest taxon distance to experimental warming (R_W_MNTD), (g) species β-diversity ($\beta Bray_w$) between the warming and non-warming conditions, (h) phylogenetic β-diversity ($\beta MNTD_w$) between the warming and non-warming conditions, (i) the response ratio of community aboveground net primary production to experimental warming (R_W_ANPP$_{community}$), (j) the response ratio of sedge aboveground net primary production to experimental warming (R_W_ANPP$_{sedge}$), (k) the response ratio of graminoid aboveground net primary production to experimental warming (R_W_ANPP$_{graminoid}$), and (l) the response ratio of forb aboveground net primary production to experimental warming (R_W_ANPP$_{forb}$).

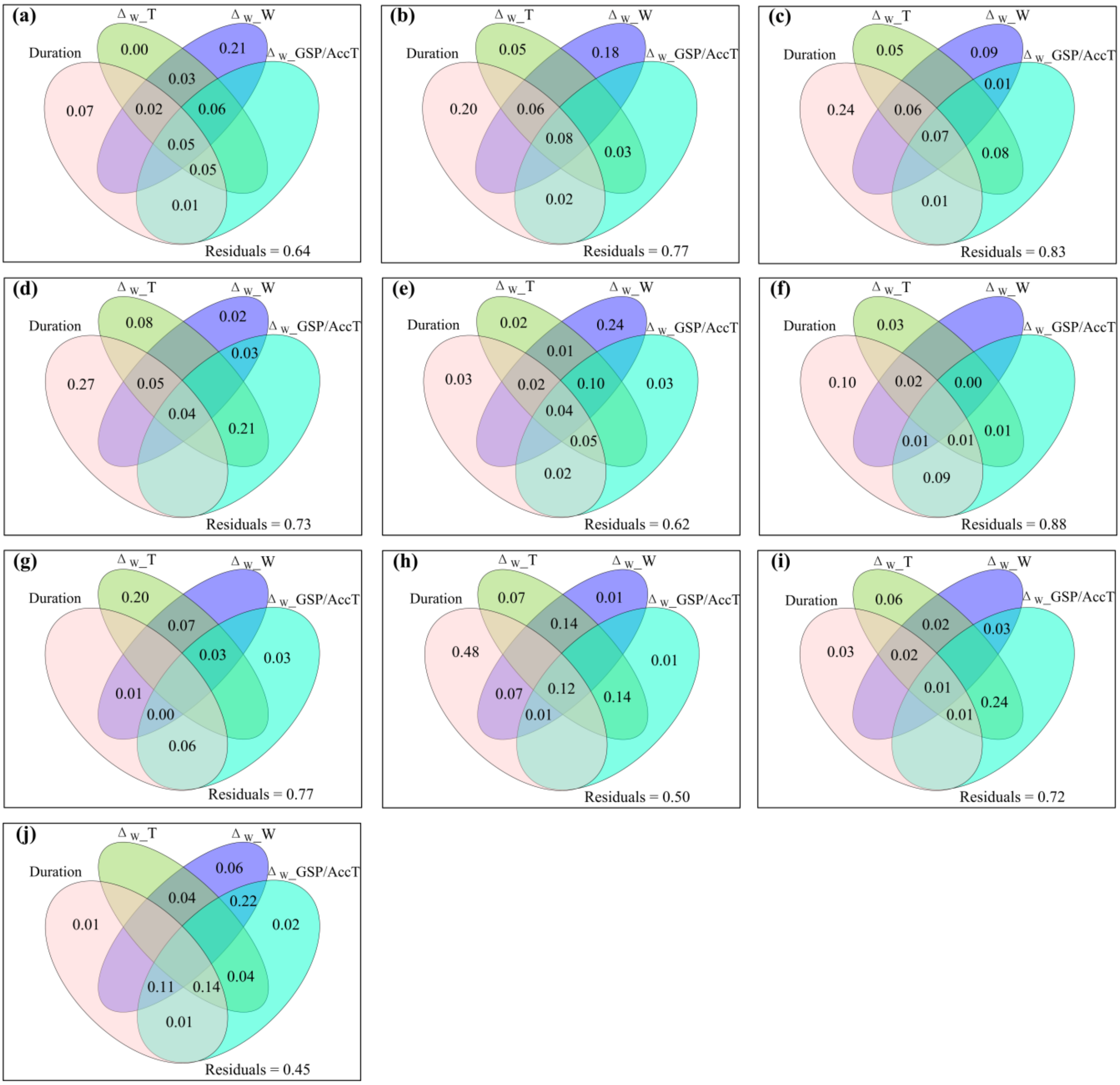

Figure S18 Venn plots of variation partitioning analysis, showing the shared and exclusive effects of warming duration, Δw_T (i.e. the change magnitude of air and/or soil temperature caused by experimental warming), Δw_W (i.e. the change magnitude of soil moisture and/or vapor pressure deficit caused by experimental warming) and Δw_GSP/AccT (i.e. the change magnitude of the ratio of growing season precipitation to accumulated >=5 ^{o}C daily air temperature caused by experimental warming) on (a) the change magnitude of species richness caused by experimental warming (Δw_SR), (b) the change magnitude of Shannon caused by experimental warming (Δw_Shannon), (c) the change magnitude of Simpson caused by experimental warming (Δw_Simpson), (d) the change magnitude of Pielou caused by experimental warming (Δw_Pielou), (e) the change magnitude of Faith's phylogenetic diversity caused by experimental warming (Δw_PD), (f) the change magnitude of mean nearest taxon distance caused by experimental warming (Δw_MNTD), (g) the change magnitude of community aboveground net primary production caused by experimental warming (Δw_ANPP$_{community}$), (h) the change magnitude of sedge aboveground net primary production caused by experimental warming (Δw_ANPP$_{sedge}$), (i) the change magnitude of graminoid aboveground net primary production caused by experimental warming (Δw_ANPP$_{graminoid}$), and (j) the change magnitude of forb aboveground net primary production caused by experimental warming (Δw_ANPP$_{forb}$).

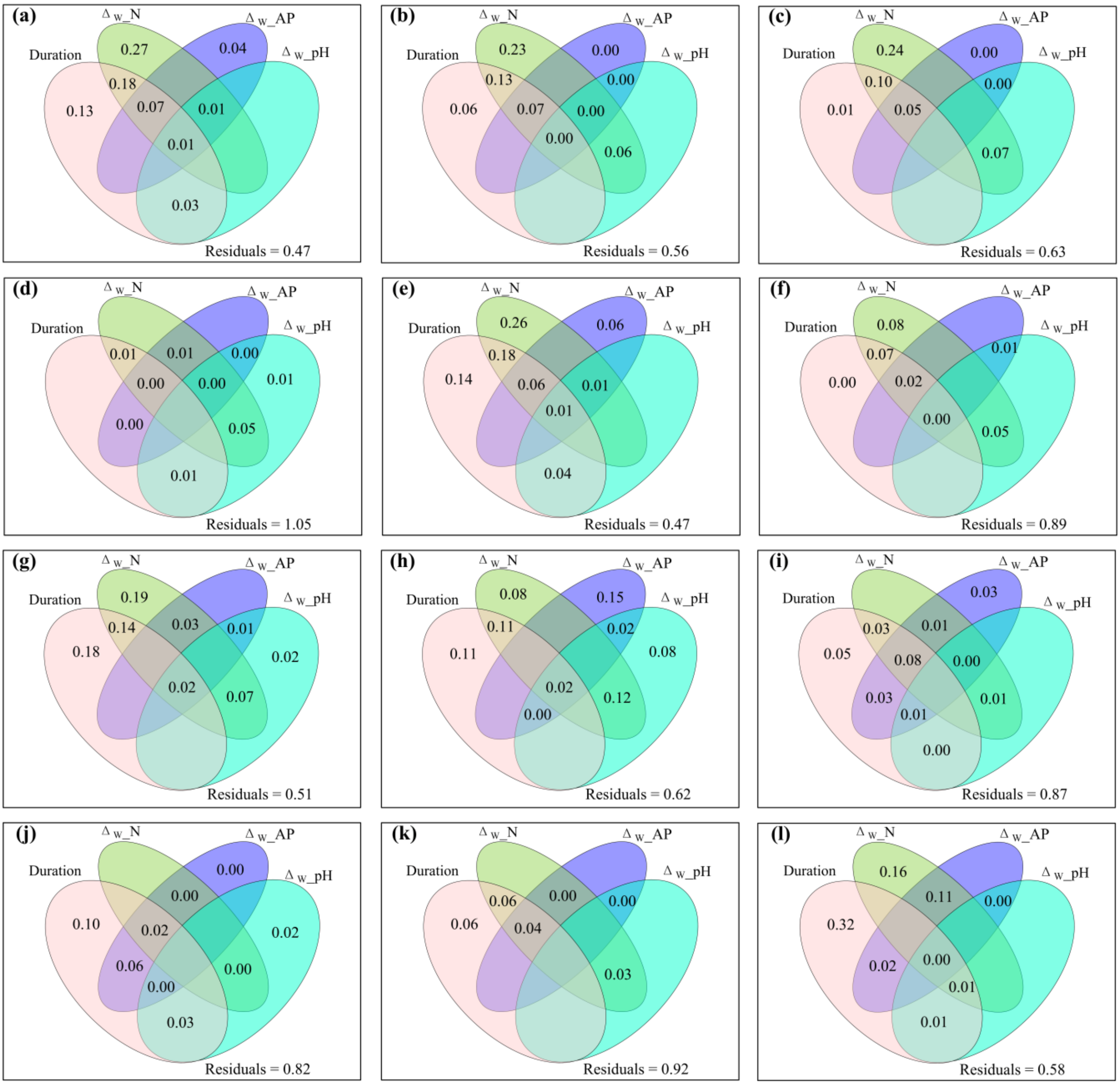

Figure S19 Venn plots of variation partitioning analysis, showing the shared and exclusive effects of warming duration, Δ_W_N (i.e. the change magnitude of ammonium nitrogen and/or nitrate nitrogen caused by experimental warming), Δ_W_AP (i.e. the change magnitude of soil available phosphorus caused by experimental warming) and Δ_W_pH (i.e. the change magnitude of soil pH caused by experimental warming) on (a) the response ratio of species richness to experimental warming (R_W_SR), (b) the response ratio of Shannon to experimental warming (R_W_Shannon), (c) the response ratio of Simpson to experimental warming (R_W_Simpson), (d) the response ratio of Pielou to experimental warming (R_W_Pielou), (e) the response ratio of Faith's phylogenetic diversity to experimental warming (R_W_PD), (f) the response ratio of mean nearest taxon distance to experimental warming (R_W_MNTD), (g) species β-diversity (βBray$_W$) between the warming and non-warming conditions, (h) phylogenetic β-diversity (βMNTD$_W$) between the warming and non-warming conditions, (i) the response ratio of community aboveground net primary production to experimental warming (R_W_ANPP$_{community}$), (j) the response ratio of sedge aboveground net primary production to experimental warming (R_W_ANPP$_{sedge}$), (k) the response ratio of graminoid aboveground net primary production to experimental warming (R_W_ANPP$_{graminoid}$), and (l) the response ratio of forb aboveground net primary production to experimental warming (R_W_ANPP$_{forb}$).

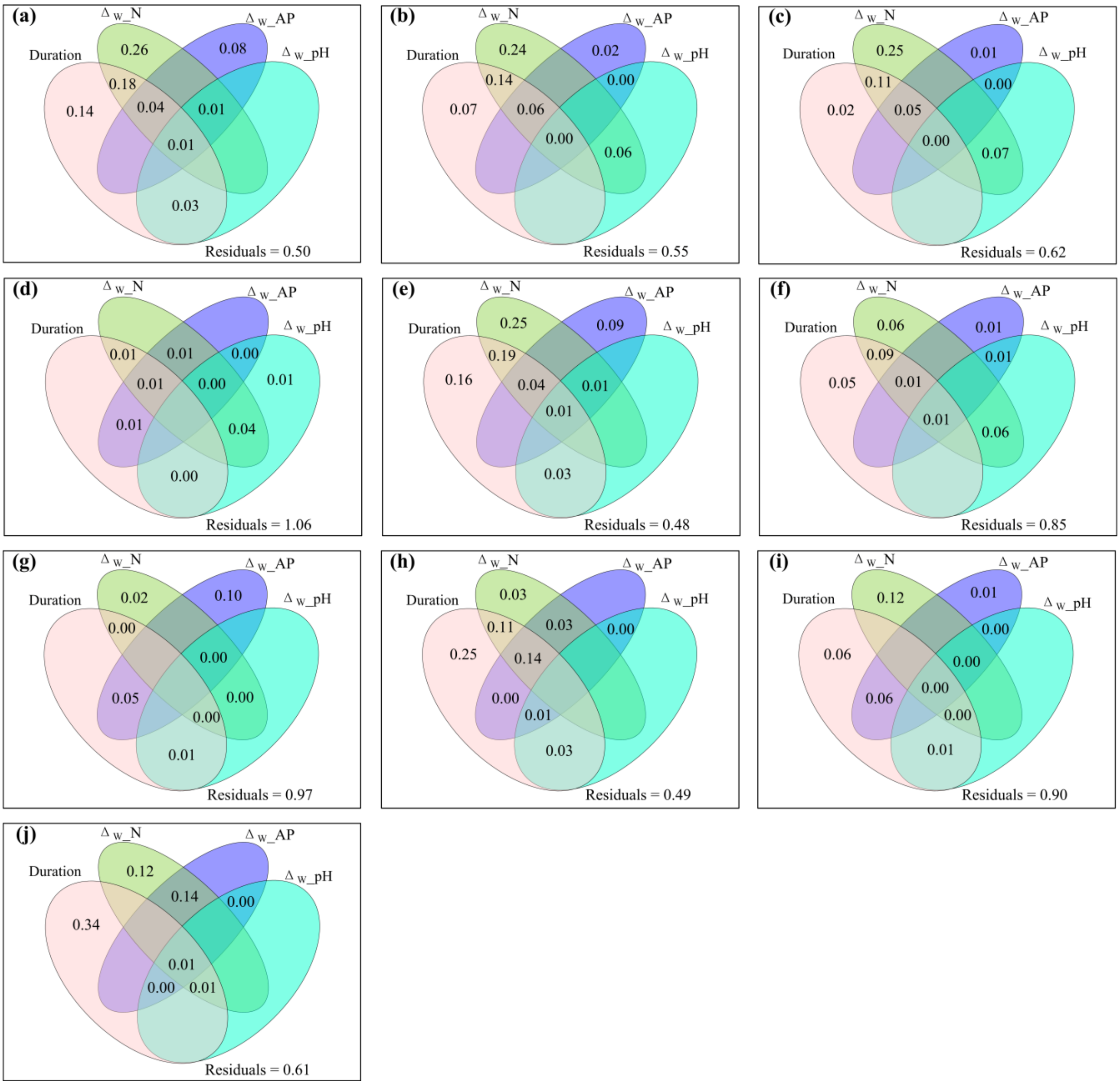

Figure S20 Venn plots of variation partitioning analysis, showing the shared and exclusive effects of warming duration, Δw_N (i.e. the change magnitude of ammonium nitrogen and/or nitrate nitrogen caused by experimental warming), Δw_AP (i.e. the change magnitude of soil available phosphorus caused by experimental warming) and Δw_pH (i.e. the change magnitude of soil pH caused by experimental warming) on (a) the change magnitude of species richness caused by experimental warming (Δw_SR), (b) the change magnitude of Shannon caused by experimental warming (Δw_Shannon), (c) the change magnitude of Simpson caused by experimental warming (Δw_Simpson), (d) the change magnitude of Pielou caused by experimental warming (Δw_Pielou), (e) the change magnitude of Faith's phylogenetic diversity caused by experimental warming (Δw_PD), (f) the change magnitude of mean nearest taxon distance caused by experimental warming (Δw_MNTD), (g) the change magnitude of community aboveground net primary production caused by experimental warming ($Δw\_ANPP_{community}$), (h) the change magnitude of sedge aboveground net primary production caused by experimental warming ($Δw\_ANPP_{sedge}$), (i) the change magnitude of graminoid aboveground net primary production caused by experimental warming ($Δw\_ANPP_{graminoid}$), and (j) the change magnitude of forb aboveground net primary production caused by experimental warming ($Δw\_ANPP_{forb}$).

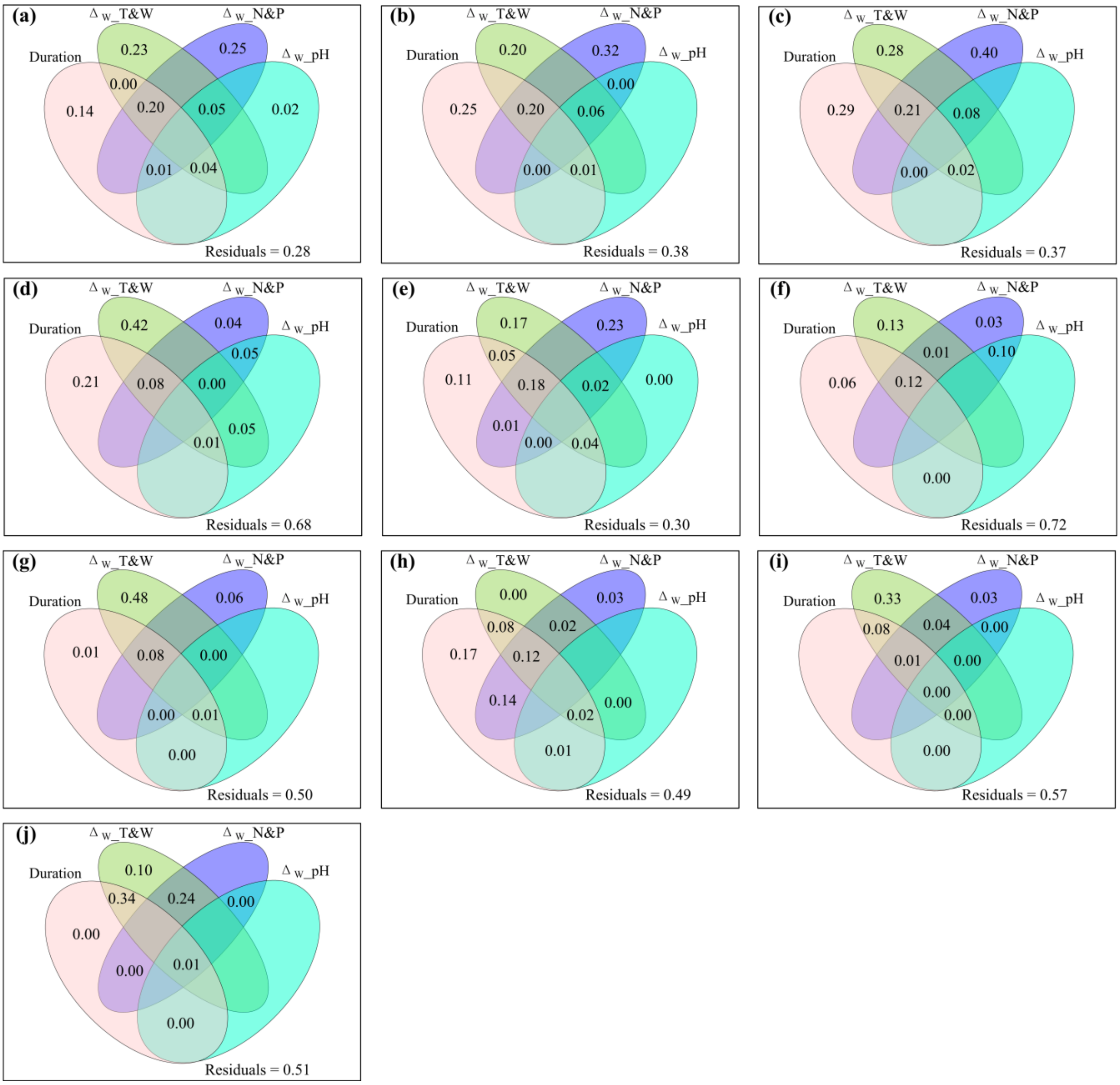

Figure S21 Venn plots of variation partitioning analysis, showing the shared and exclusive effects of warming duration, Δw_T&W (i.e. the change magnitude of air and/or soil temperature, soil moisture, vapor pressure deficit and/or the ratio of growing season precipitation to accumulated >=5 ^{o}C daily air temperature caused by experimental warming), Δw_N&P (i.e. the change magnitude of ammonium nitrogen, nitrate nitrogen, and/or available phosphorus caused by experimental warming) and Δw_pH (i.e. the change magnitude of soil pH caused by experimental warming) on (a) the change magnitude of species richness caused by experimental warming (Δw_SR), (b) the change magnitude of Shannon caused by experimental warming (Δw_Shannon), (c) the change magnitude of Simpson caused by experimental warming (Δw_Simpson), (d) the change magnitude of Pielou caused by experimental warming (Δw_Pielou), (e) the change magnitude of Faith's phylogenetic diversity caused by experimental warming (Δw_PD), (f) the change magnitude of mean nearest taxon distance caused by experimental warming (Δw_MNTD), (g) the change magnitude of community aboveground net primary production caused by experimental warming ($Δw\_ANPP_{community}$), (h) the change magnitude of sedge aboveground net primary production caused by experimental warming ($Δw\_ANPP_{sedge}$), (i) the change magnitude of graminoid aboveground net primary production caused by experimental warming ($Δw\_ANPP_{graminoid}$), and (j) the change magnitude of forb aboveground net primary production caused by experimental warming ($Δw\_ANPP_{forb}$).

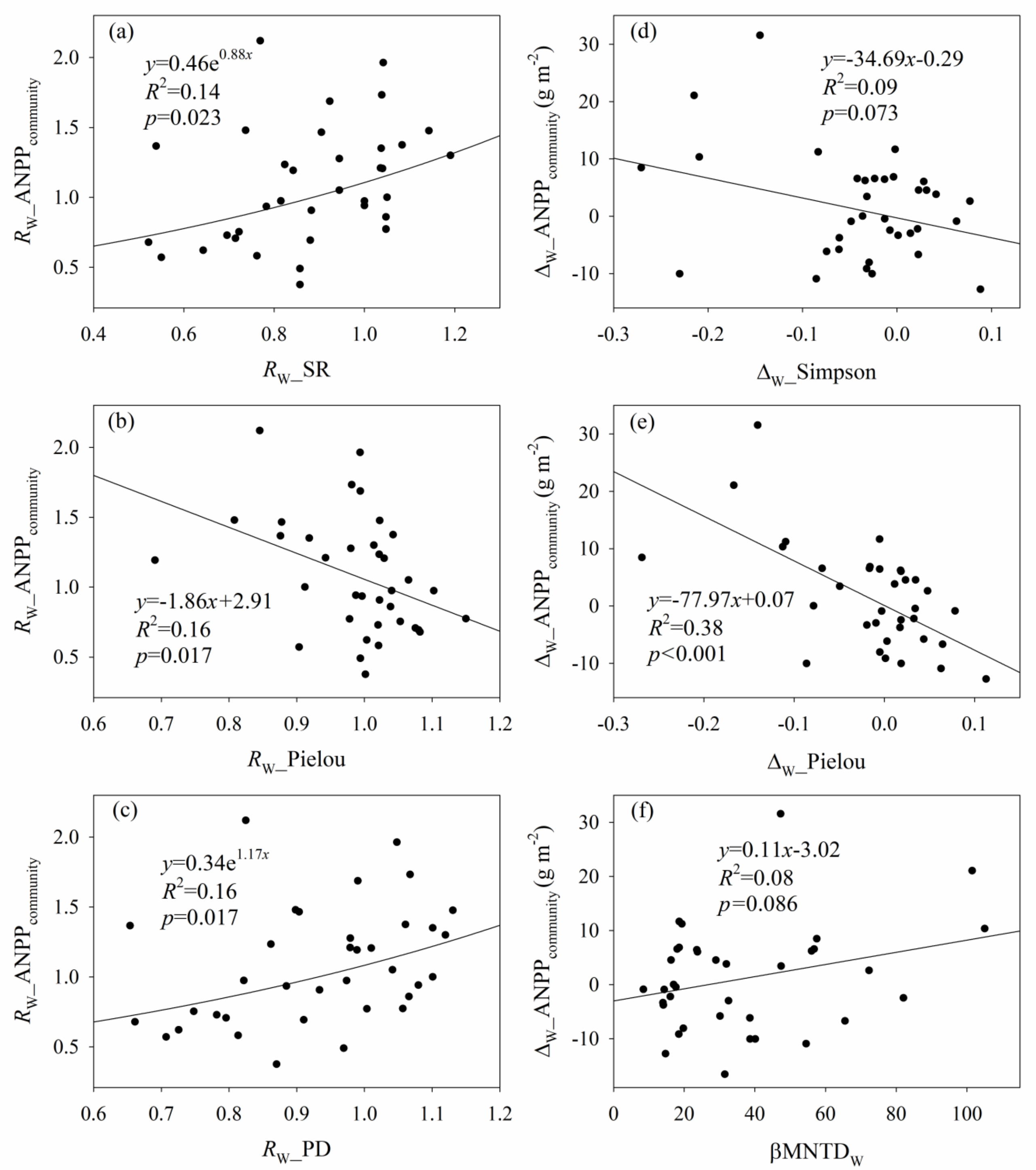

Figure S22 Relationships (a) between the response ratio of community aboveground net primary production to experimental warming (R_W_ANPP$_{community}$) and the response ratio of species richness to experimental warming (R_W_SR); (b) between the R_W_ANPP$_{community}$ and the response ratio of Pielou to experimental warming (R_W_Pielou); (c) between the R_W_ANPP$_{community}$ and the response ratio of Faith's phylogenetic diversity to experimental warming (R_W_PD); (d) the change magnitude of community aboveground net primary production caused by experimental warming (Δ_W_ANPP$_{community}$) and the change magnitude of Simpson caused by experimental warming (Δ_W_Simpson); (e) between the Δ_W_ANPP$_{community}$ and the change magnitude of Pielou caused by experimental warming (Δ_W_Pielou); and (f) between the Δ_W_ANPP$_{community}$ and the phylogenetic β-diversity of warming versus no-warming conditions ($\beta MNTD_W$).

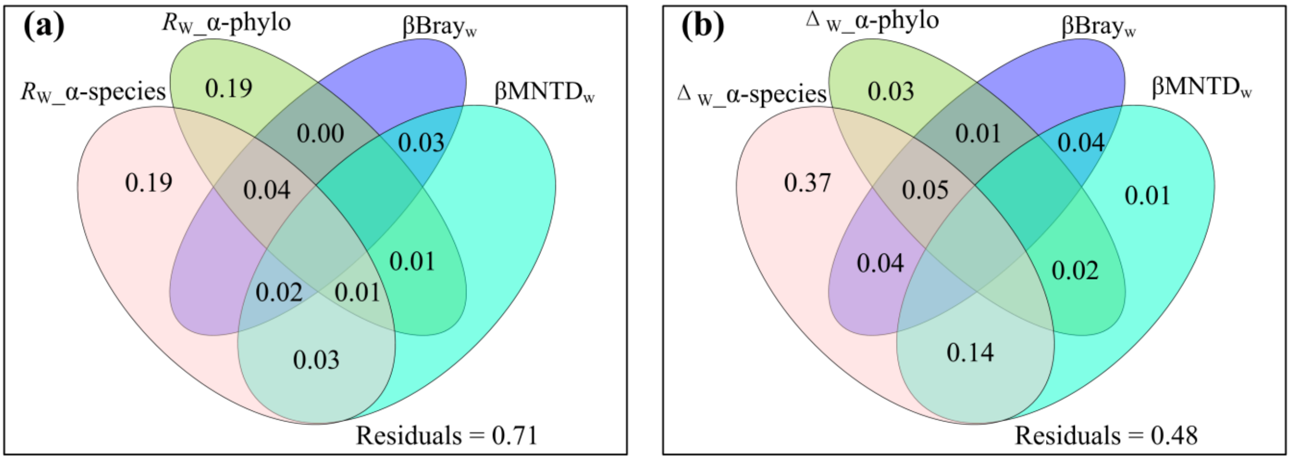

Figure S23 Venn plots of variation partitioning analysis, showing the shared and exclusive effects of (a) R_W_α-species (i.e. the response ratio of species richness, Shannon, Simpson and/or Pielou to experimental warming), R_W_α-phylo (i.e. the response ratio of Faith's phylogenetic diversity and/or mean nearest taxon distance to experimental warming), βBray (i.e. species β-diversity of warming versus non-warming conditions) and βMNTD (i.e. phylogenetic β-diversity of warming versus non-warming conditions) on the response ratio of community aboveground net primary production to experimental warming (R_W_ANPP$_{community}$); and (b) Δ_W_α-species (i.e. the change magnitude of species richness, Shannon, Simpson and/or Pielou caused by experimental warming), Δ_W_α-phylo (i.e. the change magnitude of Faith's phylogenetic diversity and/or mean nearest taxon distance caused by experimental warming), βBray (i.e. species β-diversity of warming versus non-warming conditions) and βMNTD (i.e. phylogenetic β-diversity of warming versus non-warming conditions) on the change magnitude of community aboveground net primary production caused by experimental warming (Δ_W_ANPP$_{community}$).

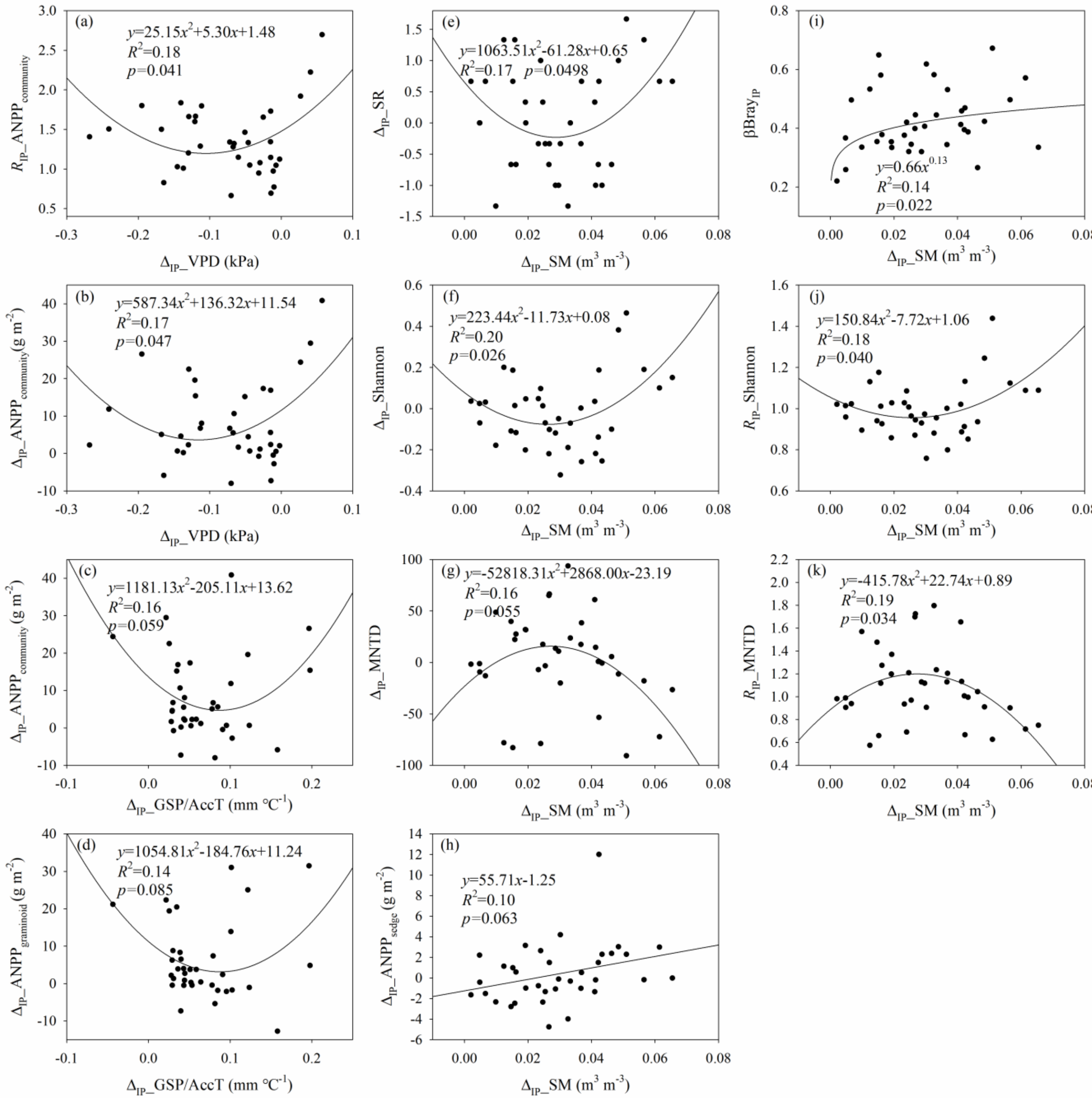

Figure S24 Relationships (a) between the response ratio of community aboveground net primary production to increased precipitation (R_{IP}_ANPP$_{community}$) and the change magnitude of vapor pressure deficit (Δ_{IP}_VPD); (b) between the change magnitude of community aboveground net primary production caused by increased precipitation (Δ_{IP}_ANPP$_{community}$) and Δ_{IP}_VPD; (c) between the Δ_{IP}_ANPP$_{community}$ and the change magnitude of the ratio of growing season precipitation to accumulated >=5 ^{o}C daily air temperature (Δ_{IP}_GSP/AccT); (d) between the change magnitude of graminoid aboveground net primary production caused by increased precipitation (Δ_{IP}_ANPP$_{graminoid}$) and Δ_{IP}_GSP/AccT; (e) between the change magnitude of species richness caused by increased precipitation (Δ_{IP}_SR) and the change magnitude of soil moisture (Δ_{IP}_SM); (f) between the change magnitude of Shannon caused by increased precipitation (Δ_{IP}_Shannon) and Δ_{IP}_SM; (g) between the change magnitude of mean nearest taxon distance caused by increased precipitation (Δ_{IP}_MNTD) and Δ_{IP}_SM; (h) between the change magnitude of sedge aboveground net primary production caused by increased precipitation (Δ_{IP}_ANPP$_{sedge}$) and Δ_{IP}_SM; (i) between the species β-diversity of the increased versus no-increased precipitation conditions (βBray$_{IP}$) and Δ_{IP}_SM; (j) between the response ratio of Shannon to increased precipitation (R_{IP}_Shannon) and Δ_{IP}_SM; and (k) between the response ratio of mean nearest taxon distance to increased precipitation (R_{IP}_MNTD) and Δ_{IP}_SM.

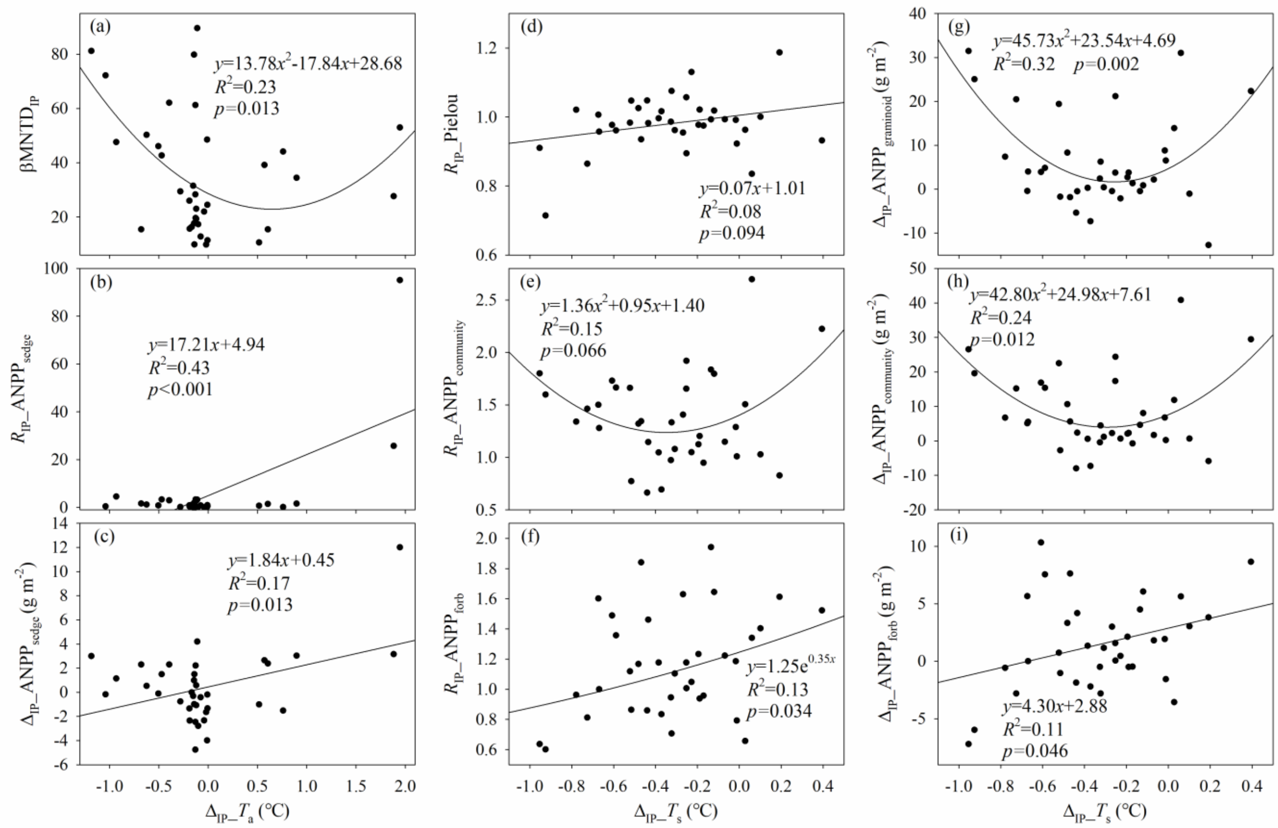

Figure S25 Relationships (a) the phylogenetic β-diversity of the increased versus no-increased precipitation conditions ($\beta MNTD_{IP}$) and the change magnitude of air temperature caused by increased precipitation (Δ_{IP}_T_a); (b) between the response ratio of sedge aboveground net primary production to increased precipitation (R_{IP}_$ANPP_{sedge}$) and Δ_{IP}_T_a; (c) between the change magnitude of sedge aboveground net primary production caused by increased precipitation (Δ_{IP}_$ANPP_{sedge}$) and Δ_{IP}_T_a; (d) between the response ratio of Pielou to increased precipitation (R_{IP}_Pielou) and the change magnitude of soil temperature caused by increased precipitation (Δ_{IP}_T_s); (e) between the response ratio of community aboveground net primary production to increased precipitation (R_{IP}_$ANPP_{community}$) and the Δ_{IP}_T_s; (f) between the response ratio of forb aboveground net primary production to increased precipitation (R_{IP}_$ANPP_{forb}$) and the Δ_{IP}_T_s; (g) between the change magnitude of graminoid aboveground net primary production caused by increased precipitation (Δ_{IP}_ $ANPP_{graminoid}$) and Δ_{IP}_T_s; (h) between the change magnitude of community aboveground net primary production caused by increased precipitation (Δ_{IP}_ $ANPP_{community}$) and Δ_{IP}_T_s; and (i) between the change magnitude of forb aboveground net primary production caused by increased precipitation (Δ_{IP}_ $ANPP_{forb}$) and Δ_{IP}_T_s.

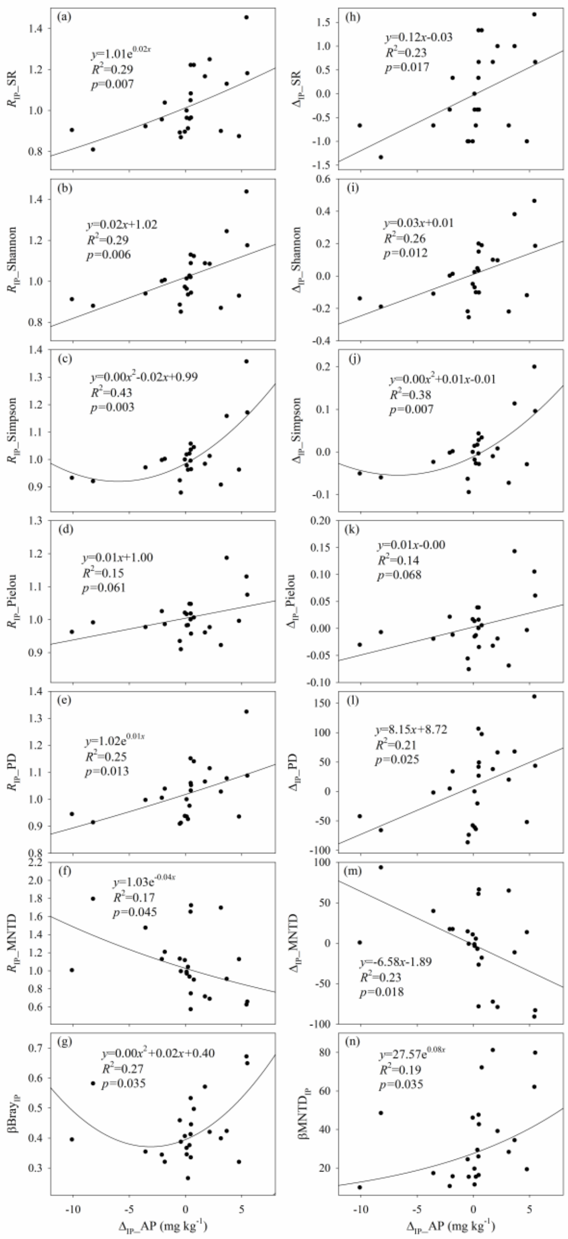

Figure S26 Relationships (a) between the response ratio of species richness to increased precipitation (R_{IP}_SR) and the change magnitude of available phosphorus caused by increased precipitation (Δ_{IP}_AP); (b) between the response ratio of Shannon to increased precipitation (R_{IP}_Shannon) and Δ_{IP}_AP; (c) between the response ratio of Simpson to increased precipitation (R_{IP}_Simpson) and Δ_{IP}_AP; (d) between the response ratio of Pielou to increased precipitation (R_{IP}_Pielou) and Δ_{IP}_AP; (e) between the response ratio of phylogenetic diversity to increased precipitation (R_{IP}_PD) and Δ_{IP}_AP; (f) between the response ratio of mean nearest taxon distance to increased precipitation (R_{IP}_MNTD) and Δ_{IP}_AP; (g) between the species β-diversity of the increased versus no-increased precipitation conditions ($\beta\mathrm{Bray}_{\mathrm{IP}}$) and Δ_{IP}_AP; (h) between the change magnitude of species richness caused by increased precipitation (Δ_{IP}_SR) and Δ_{IP}_AP; (i) between the change magnitude of Shannon caused by increased precipitation (Δ_{IP}_Shannon) and Δ_{IP}_AP; (j) between the change magnitude of Simpson caused by increased precipitation (Δ_{IP}_Simpson) and Δ_{IP}_AP; (k) between the change magnitude of Pielou caused by increased precipitation (Δ_{IP}_Pielou) and Δ_{IP}_AP; (l) between the change magnitude of phylogenetic diversity caused by increased precipitation (Δ_{IP}_PD) and Δ_{IP}_AP; (m) between the change magnitude of mean nearest taxon distance caused by increased precipitation (Δ_{IP}_MNTD) and Δ_{IP}_AP; and (n) between the phylogenetic β-diversity of the increased versus no-increased precipitation conditions ($\beta\mathrm{MNTD}_{\mathrm{IP}}$) and Δ_{IP}_AP.

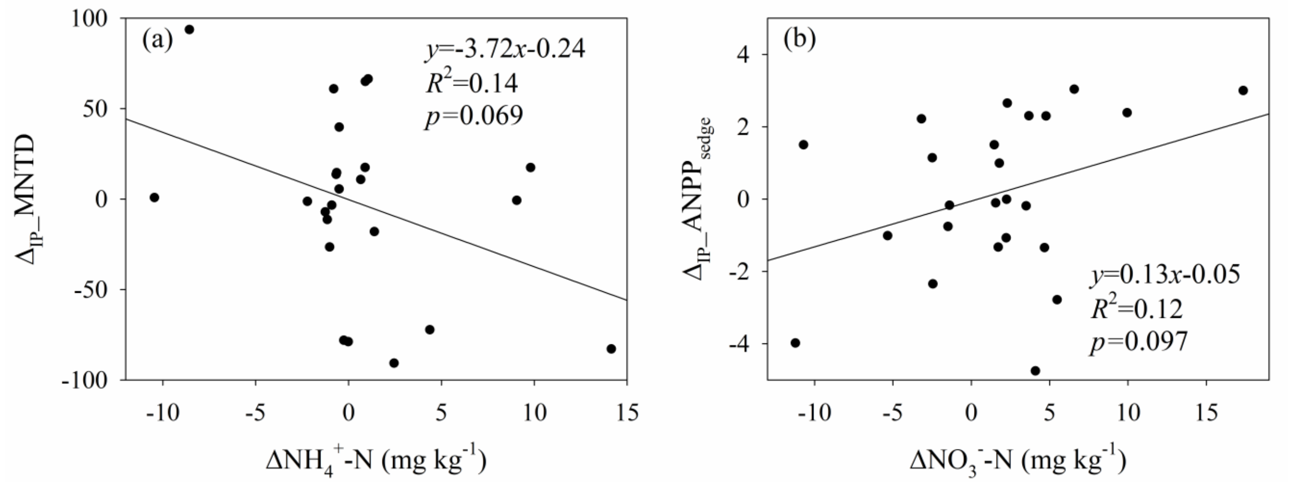

Figure S27 Relationships (a) between the change magnitude of mean nearest taxon distance caused by increased precipitation (Δ_{IP}_MNTD) and the change magnitude of ammonium nitrogen caused by increased precipitation (Δ_{IP}_NH_4^+-N); and (b) between the change magnitude of sedge aboveground net primary production caused by increased precipitation (Δ_{IP}_$ANPP_{sedge}$) and the change magnitude of nitrate nitrogen caused by increased precipitation (Δ_{IP}_NO_3^--N).

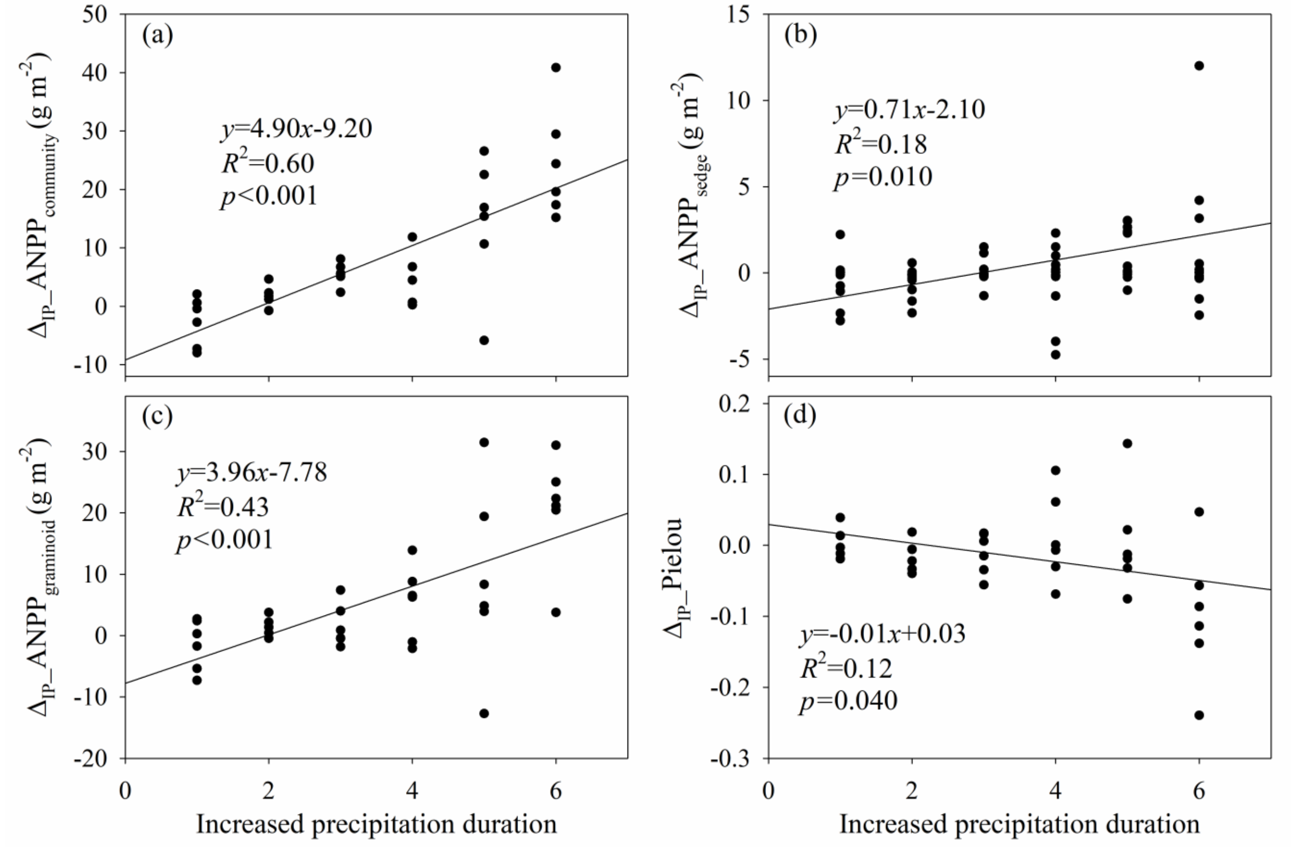

Figure S28 Relationships (a) between the change magnitude of community aboveground net primary production caused by increased precipitation (Δ_{IP}_$ANPP_{community}$) and increased precipitation duration; (b) between the change magnitude of sedge aboveground net primary production caused by increased precipitation (Δ_{IP}_$ANPP_{sedge}$) and increased precipitation duration; (c) between the change magnitude of graminoid aboveground net primary production caused by increased precipitation (Δ_{IP}_$ANPP_{graminoid}$) and increased precipitation duration; and (d) between the change magnitude of Pielou caused by increased precipitation (Δ_{IP}_Pielou) and increased precipitation duration.

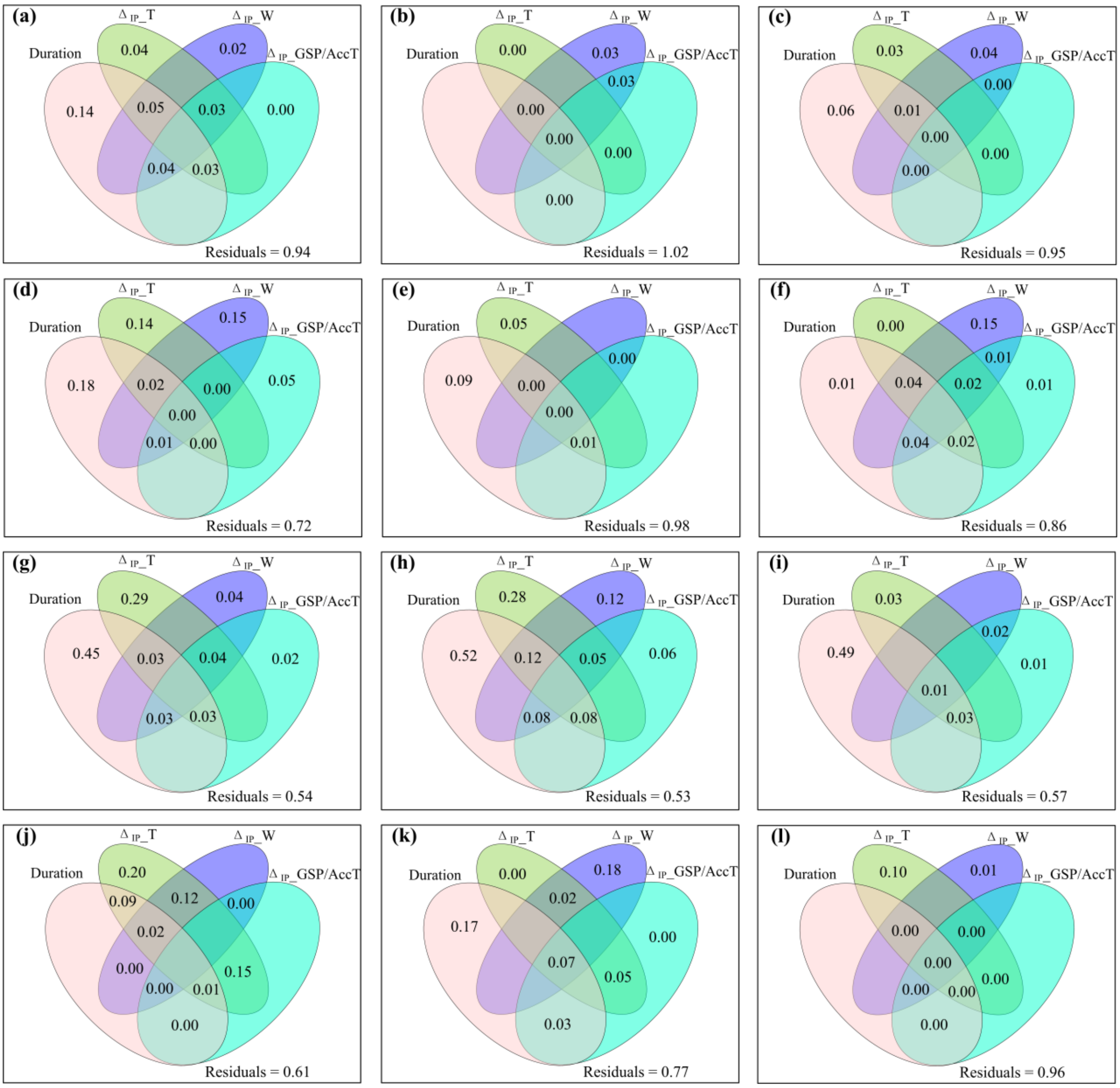

Figure S29 Venn plots of variation partitioning analysis, showing the shared and exclusive effects of increased precipitation duration, Δ$_{IP}$_T (i.e. the change magnitude of air and/or soil temperature caused by increased precipitation), Δ$_{IP}$_W (i.e. the change magnitude of growing season precipitation, soil moisture and/or vapor pressure deficit caused by increased precipitation) and Δ$_{IP}$_GSP/AccT (i.e. the change magnitude of the ratio of growing season precipitation to accumulated >=5 °C daily air temperature caused by increased precipitation) on (a) the response ratio of species richness to increased precipitation (R_{IP}_SR), (b) the response ratio of Shannon to increased precipitation (R_{IP}_Shannon), (c) the response ratio of Simpson to increased precipitation (R_{IP}_Simpson), (d) the response ratio of Pielou to increased precipitation (R_{IP}_Pielou), (e) the response ratio of Faith's phylogenetic diversity to increased precipitation (R_{IP}_PD), (f) the response ratio of mean nearest taxon distance to increased precipitation (R_{IP}_MNTD), (g) species β-diversity ($\beta Bray_{IP}$) between the increased precipitation and non-increased precipitation conditions, (h) phylogenetic β-diversity ($\beta MNTD_{IP}$) between the increased precipitation and non-increased precipitation conditions, (i) the response ratio of community aboveground net primary production to increased precipitation (R_{IP}_ANPP$_{community}$), (j) the response ratio of sedge aboveground net primary production to increased precipitation (R_{IP}_ANPP$_{sedge}$), (k) the response ratio of graminoid aboveground net primary production to increased precipitation (R_{IP}_ANPP$_{graminoid}$), and (l) the response ratio of forb aboveground net primary production to increased precipitation (R_{IP}_ANPP$_{forb}$).

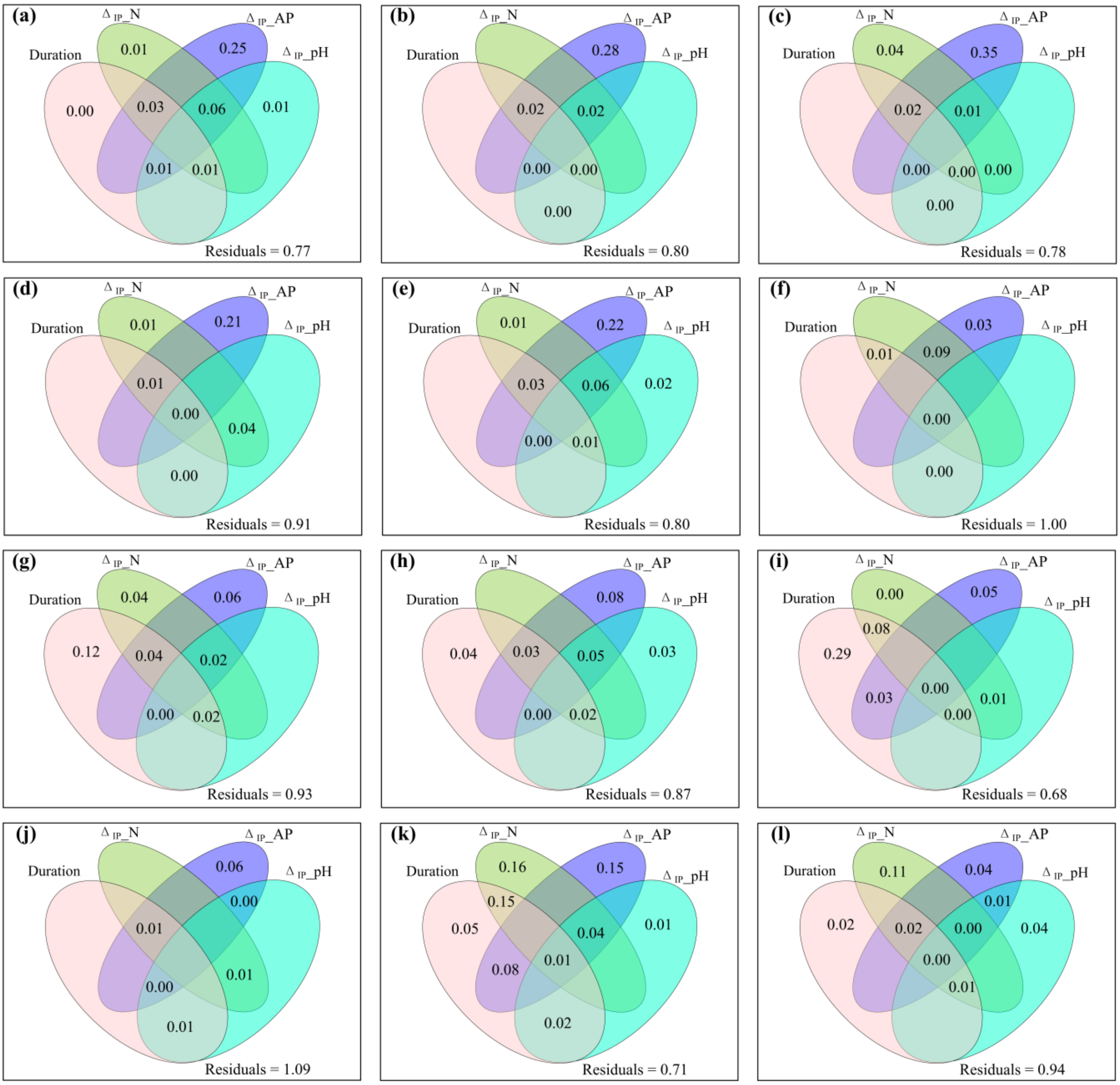

Figure S30 Venn plots of variation partitioning analysis, showing the shared and exclusive effects of increased precipitation duration, Δ_{IP}_N (i.e. the change magnitude of ammonium nitrogen and/or nitrate nitrogen caused by increased precipitation), Δ_{IP}_AP (i.e. the change magnitude of soil available phosphorus caused by increased precipitation) and Δ_{IP}_pH (i.e. the change magnitude of soil pH caused by increased precipitation) on (a) the response ratio of species richness to increased precipitation (R_{IP}_SR), (b) the response ratio of Shannon to increased precipitation (R_{IP}_Shannon), (c) the response ratio of Simpson to increased precipitation (R_{IP}_Simpson), (d) the response ratio of Pielou to increased precipitation (R_{IP}_Pielou), (e) the response ratio of Faith's phylogenetic diversity to increased precipitation (R_{IP}_PD), (f) the response ratio of mean nearest taxon distance to increased precipitation (R_{IP}_MNTD), (g) species β-diversity ($\beta Bray_{IP}$) between the increased precipitation and non-increased precipitation conditions, (h) phylogenetic β-diversity ($\beta MNTD_{IP}$) between the increased precipitation and non-increased precipitation conditions, (i) the response ratio of community aboveground net primary production to increased precipitation (R_{IP}_ANPP$_{community}$), (j) the response ratio of sedge aboveground net primary production to increased precipitation (R_{IP}_ANPP$_{sedge}$), (k) the response ratio of graminoid aboveground net primary production to increased precipitation (R_{IP}_ANPP$_{graminoid}$), and (l) the response ratio of forb aboveground net primary production to increased precipitation (R_{IP}_ANPP$_{forb}$).

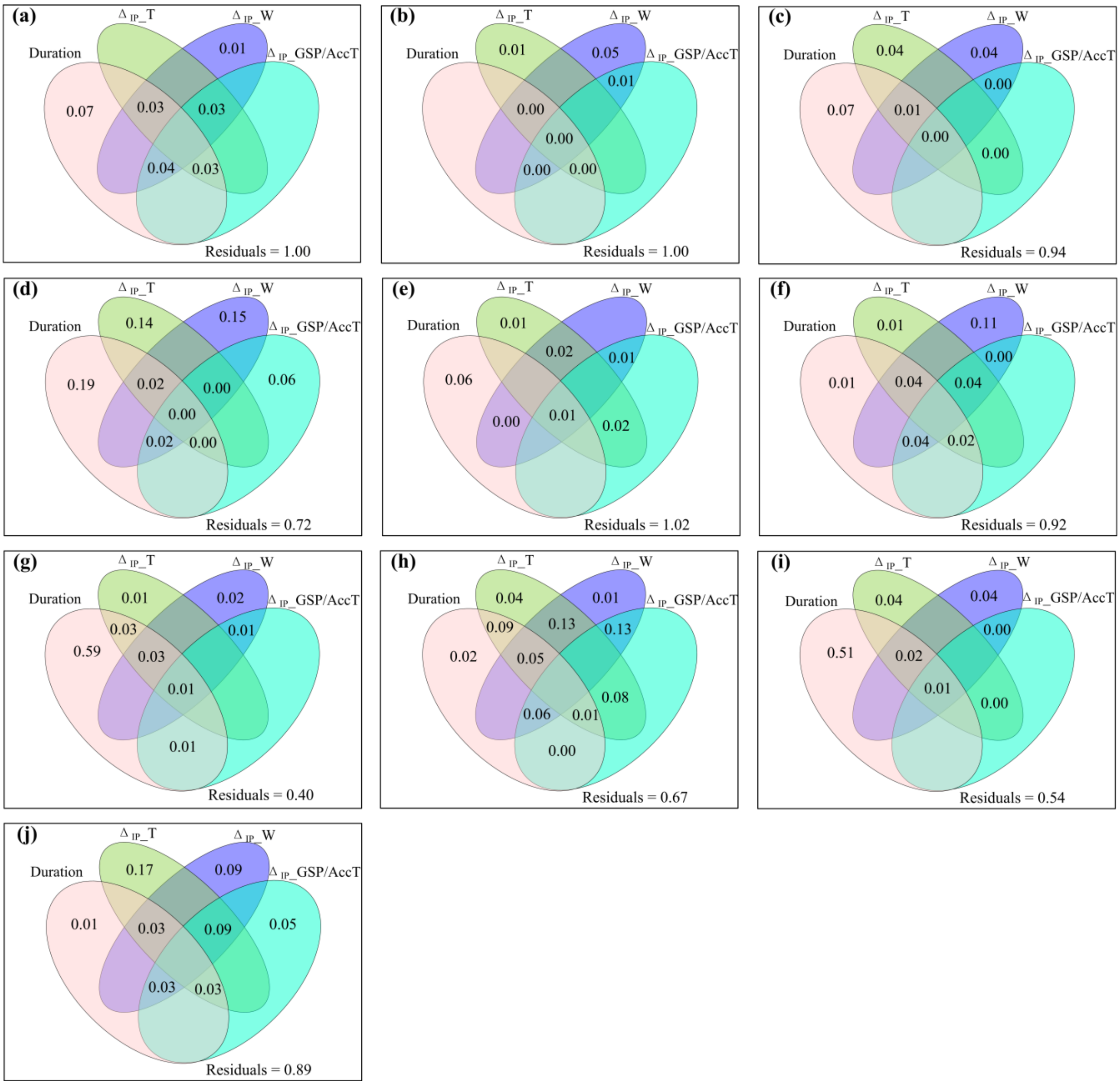

Figure S31 Venn plots of variation partitioning analysis, showing the shared and exclusive effects of increased precipitation duration, Δ_{IP}_T (i.e. the change magnitude of air and/or soil temperature caused by increased precipitation), Δ_{IP}_W (i.e. the change magnitude of growing season precipitation, soil moisture and/or vapor pressure deficit caused by increased precipitation) and Δ_{IP}_GSP/AccT (i.e. the change magnitude of the ratio of growing season precipitation to accumulated >=5 ^{o}C daily air temperature caused by increased precipitation) on (a) the change magnitude of species richness caused by increased precipitation (Δ_{IP}_SR), (b) the change magnitude of Shannon caused by increased precipitation (Δ_{IP}_Shannon), (c) the change magnitude of Simpson caused by increased precipitation (Δ_{IP}_Simpson), (d) the change magnitude of Pielou caused by increased precipitation (Δ_{IP}_Pielou), (e) the change magnitude of Faith's phylogenetic diversity caused by increased precipitation (Δ_{IP}_PD), (f) the change magnitude of mean nearest taxon distance caused by increased precipitation (Δ_{IP}_MNTD), (g) the change magnitude of community aboveground net primary production caused by increased precipitation (Δ_{IP}_ANPP$_{community}$), (h) the change magnitude of sedge aboveground net primary production caused by increased precipitation (Δ_{IP}_ANPP$_{sedge}$), (i) the change magnitude of graminoid aboveground net primary production caused by increased precipitation (Δ_{IP}_ANPP$_{graminoid}$), and (j) the change magnitude of forb aboveground net primary production caused by increased precipitation (Δ_{IP}_ANPP$_{forb}$).

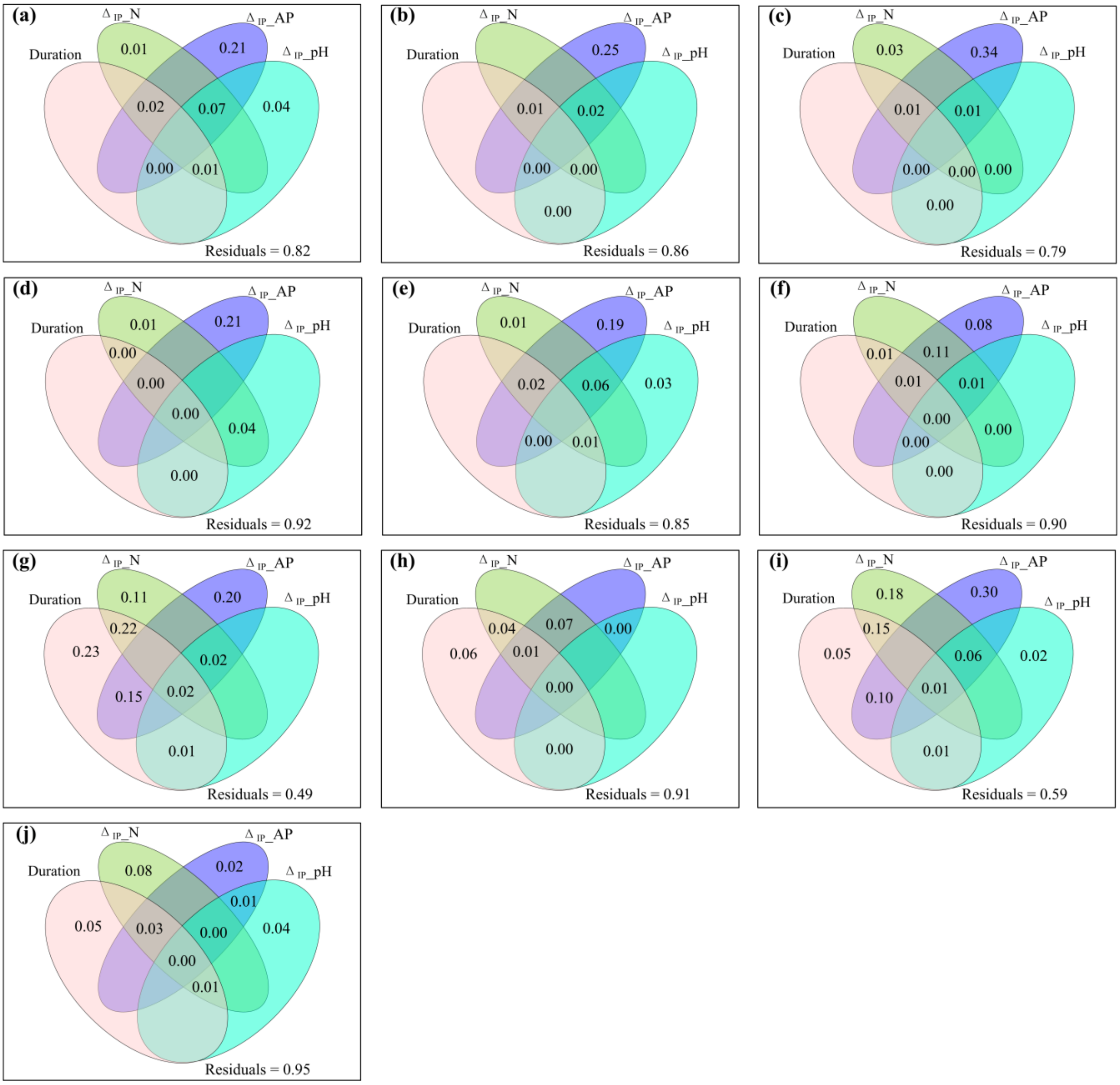

Figure S32 Venn plots of variation partitioning analysis, showing the shared and exclusive effects of increased precipitation duration, Δ_{IP}_N (i.e. the change magnitude of ammonium nitrogen and/or nitrate nitrogen caused by increased precipitation), Δ_{IP}_AP (i.e. the change magnitude of soil available phosphorus caused by increased precipitation) and Δ_{IP}_pH (i.e. the change magnitude of soil pH caused by increased precipitation) on (a) the change magnitude of species richness caused by increased precipitation (Δ_{IP}_SR), (b) the change magnitude of Shannon caused by increased precipitation (Δ_{IP}_Shannon), (c) the change magnitude of Simpson caused by increased precipitation (Δ_{IP}_Simpson), (d) the change magnitude of Pielou caused by increased precipitation (Δ_{IP}_Pielou), (e) the change magnitude of Faith's phylogenetic diversity caused by increased precipitation (Δ_{IP}_PD), (f) the change magnitude of mean nearest taxon distance caused by increased precipitation (Δ_{IP}_MNTD), (g) the change magnitude of community aboveground net primary production caused by increased precipitation (Δ_{IP}_ANPP$_{community}$), (h) the change magnitude of sedge aboveground net primary production caused by increased precipitation (Δ_{IP}_ANPP$_{sedge}$), (i) the change magnitude of graminoid aboveground net primary production caused by increased precipitation (Δ_{IP}_ANPP$_{graminoid}$), and (j) the change magnitude of forb aboveground net primary production caused by increased precipitation (Δ_{IP}_ANPP$_{forb}$).

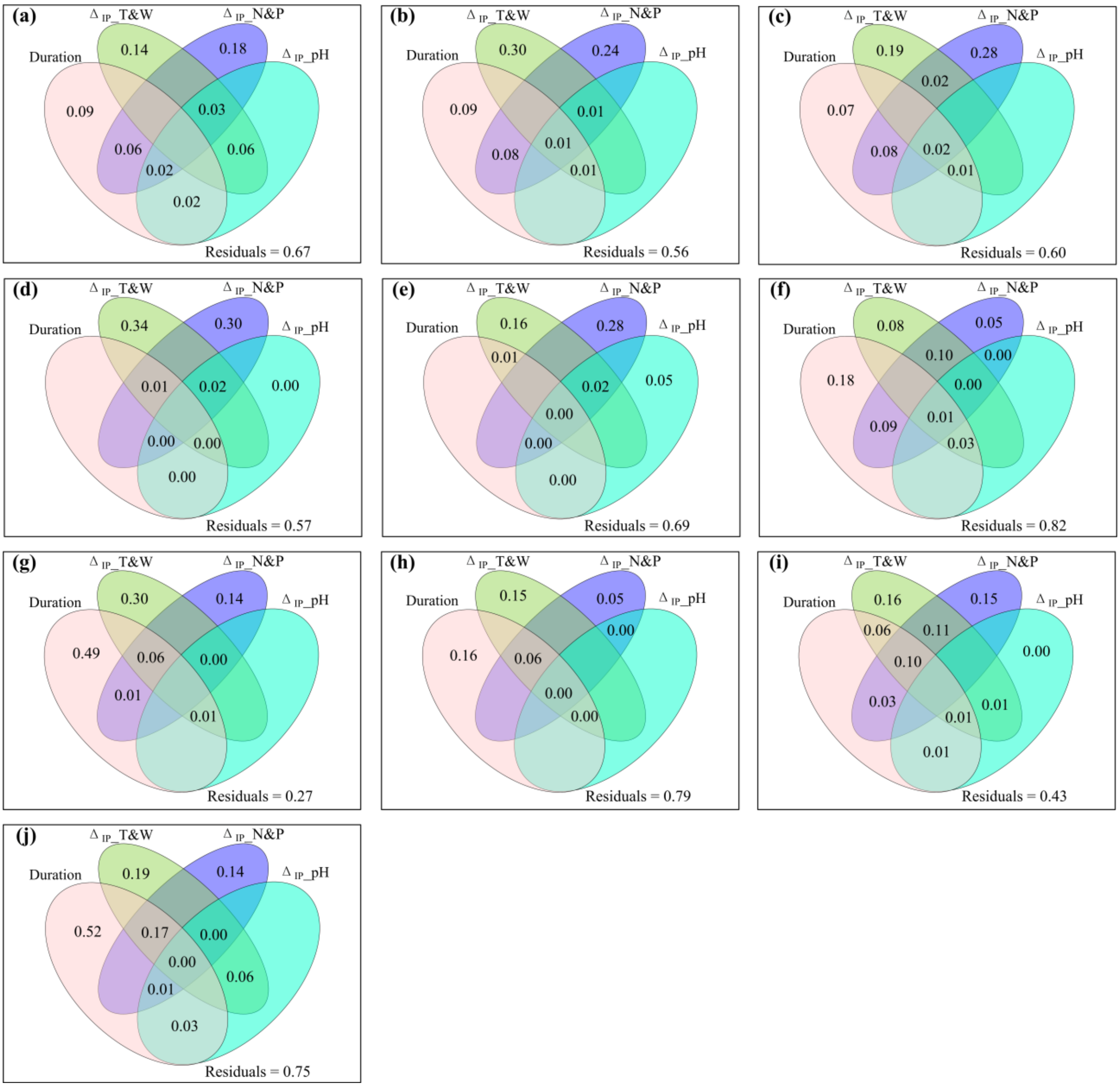

Figure S33 Venn plots of variation partitioning analysis, showing the shared and exclusive effects of increased precipitation duration, Δ_{IP}_T&W (i.e. the change magnitude of air and/or soil temperature, soil moisture, vapor pressure deficit and/or the ratio of growing season precipitation to accumulated >=5 ^{o}C daily air temperature caused by increased precipitation), Δ_{IP}_N&P (i.e. the change magnitude of ammonium nitrogen, nitrate nitrogen, and/or available phosphorus caused by increased precipitation) and Δ_{IP}_pH (i.e. the change magnitude of soil pH caused by increased precipitation) on (a) the change magnitude of species richness caused by increased precipitation (Δ_{IP}_SR), (b) the change magnitude of Shannon caused by increased precipitation (Δ_{IP}_Shannon), (c) the change magnitude of Simpson caused by increased precipitation (Δ_{IP}_Simpson), (d) the change magnitude of Pielou caused by increased precipitation (Δ_{IP}_Pielou), (e) the change magnitude of Faith's phylogenetic diversity caused by increased precipitation (Δ_{IP}_PD), (f) the change magnitude of mean nearest taxon distance caused by increased precipitation (Δ_{IP}_MNTD), (g) the change magnitude of community aboveground net primary production caused by increased precipitation (Δ_{IP}_ANPP$_{community}$), (h) the change magnitude of sedge aboveground net primary production caused by increased precipitation (Δ_{IP}_ANPP$_{sedge}$), (i) the change magnitude of graminoid aboveground net primary production caused by increased precipitation (Δ_{IP}_ANPP$_{graminoid}$), and (j) the change magnitude of forb aboveground net primary production caused by increased precipitation (Δ_{IP}_ANPP$_{forb}$).

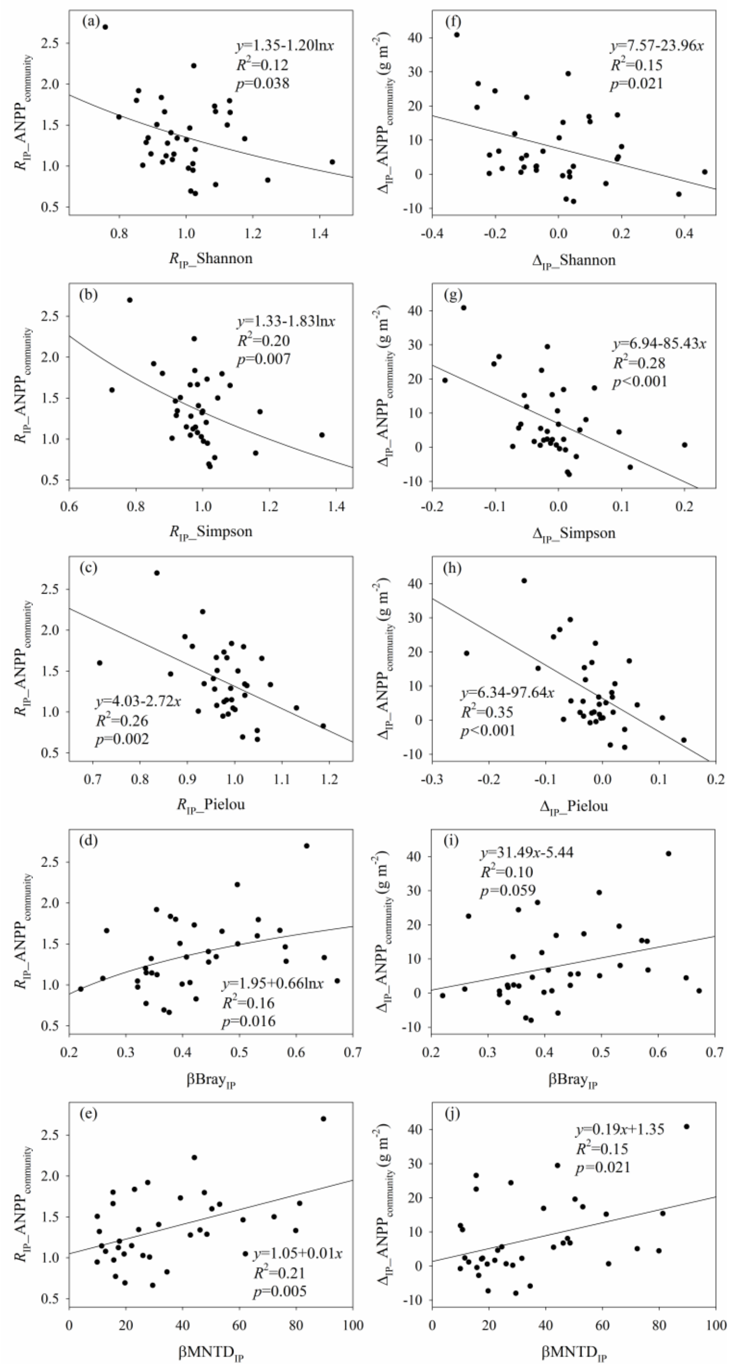

Figure S34 Relationships (a) between the response ratio of community aboveground net primary production to increased precipitation (R_{IP}_ANPP$_{community}$) and the response ratio of Shannon to increased precipitation (R_{IP}_Shannon); (b) between the R_{IP}_ANPP$_{community}$ and the response ratio of Simpson to increased precipitation (R_{IP}_Simpson); (c) between the R_{IP}_ANPP$_{community}$ and the response ratio of Pielou to increased precipitation (R_{IP}_Pielou); (d) between the R_{IP}_ANPP$_{community}$ and the species β-diversity of increased precipitation versus non-increased precipitation conditions (βBray$_{IP}$); (e) between the R_{IP}_ANPP$_{community}$ and the phylogenetic β-diversity of increased precipitation versus non-increased precipitation conditions (βMNTD$_{IP}$); (f) between the change magnitude of community aboveground net primary production caused by increased precipitation (Δ_{IP}_ANPP$_{community}$) and the change magnitude of Shannon caused by increased precipitation (Δ_{IP}_Shannon); (g) between the Δ_{IP}_ANPP$_{community}$ and the change magnitude of Simpson caused by increased precipitation (Δ_{IP}_Simpson); (h) between the Δ_{IP}_ANPP$_{community}$ and the change magnitude of Pielou caused by increased precipitation (Δ_{IP}_Pielou); (i) between the Δ_{IP}_ANPP$_{community}$ and βBray$_{IP}$; and (j) between the Δ_{IP}_ANPP$_{community}$ and βMNTD$_{IP}$.

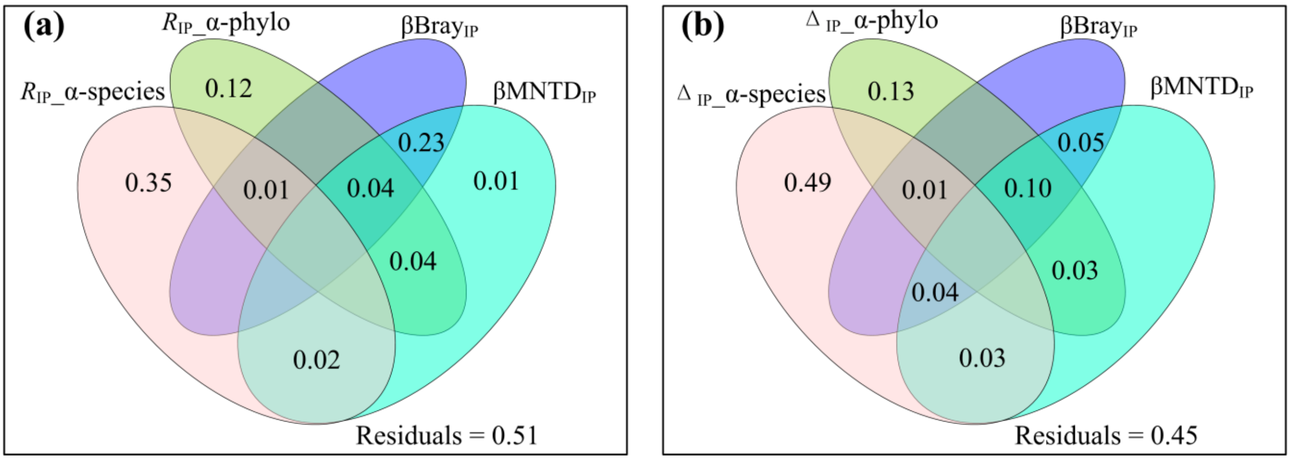

Figure S35 Venn plots of variation partitioning analysis, showing the shared and exclusive effects of (a) R_{IP}_α-species (i.e. the response ratio of species richness, Shannon, Simpson and/or Pielou to increased precipitation), R_{IP}_α-phylo (i.e. the response ratio of Faith's phylogenetic diversity and/or mean nearest taxon distance to increased precipitation), βBray (i.e. species β-diversity of increased precipitation versus non-increased precipitation conditions) and βMNTD (i.e. phylogenetic β-diversity of increased precipitation versus non-increased precipitation conditions) on the response ratio of community aboveground net primary production to increased precipitation (R_{IP}_$ANPP_{community}$); and (b) Δ_{IP}_α-species (i.e. the change magnitude of species richness, Shannon, Simpson and/or Pielou caused by increased precipitation), Δ_{IP}_α-phylo (i.e. the change magnitude of Faith's phylogenetic diversity and/or mean nearest taxon distance caused by increased precipitation) , βBray (i.e. species β-diversity of increased precipitation versus non-increased precipitation conditions) and βMNTD (i.e. phylogenetic β-diversity of increased precipitation versus non-increased precipitation conditions) on the change magnitude of community aboveground net primary production caused by increased precipitation (Δ_{IP}_$ANPP_{community}$).
